# Overcoming PEG Antigenicity: Statistical PEG Isomers Reduce Antibody Binding

**DOI:** 10.1002/advs.202521061

**Published:** 2025-11-19

**Authors:** Mareike Deuker, Dominik Schulz, Kaloian Koynov, Svenja Morsbach, Holger Frey, Katharina Landfester

**Affiliations:** ^1^ Max Planck Institute for Polymer Research Ackermannweg 10 55128 Mainz Germany; ^2^ Department of Chemistry Johannes Gutenberg University Mainz Duesbergweg 10‐14 55128 Mainz Germany

**Keywords:** anti‐PEG antibodies, copolymerization, poly(ethylene glycol), randomized PEG, ring‐opening polymerization

## Abstract

Poly(ethylene glycol) (PEG) is widely used in bioconjugation and nanomedicine due to its advantageous properties including solubility, biocompatibility, and pharmacokinetic benefits. However, PEG exposure can elicit anti‐PEG antibodies (APAs), resulting in accelerated blood clearance and immune responses. To address this, “randomized PEG” (rPEG) is synthesized by incorporating the comonomer glycidyl methyl ether into PEG via anionic ring‐opening polymerization. This structural isomer introduces “synthetic point mutations”, impeding APA recognition. The interaction between rPEGs and APAs is systematically investigated in buffer solution using microscale thermophoresis and fluorescence correlation spectroscopy. Increasing comonomer content drastically reduced antibody binding, with rPEGs containing 52 mol% comonomer effectively preventing APA interaction. Monte Carlo simulations confirmed a reduction in consecutive ethylene glycol sequences to less than 16 units crucial for APA recognition. These results establish rPEG as a promising PEG alternative, mitigating immune recognition while retaining key properties. This is of key relevance for next‐generation stealth polymers with improved biocompatibility for biomedical applications.

## Graphical abstract

Conventional poly(ethylene glycol) (PEG) is commonly applied in biomedicine, but is now known to induce antibody formation. Here, it is shown that the development of “randomized PEG” (rPEG) featuring glycidyl methyl ether (GME) comonomers decreased antibody binding. Most importantly, antibody interaction is completely suppressed at a fraction of ≥52 mol% GME, enabling the application of rPEGs as a viable PEG alternative.

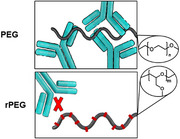

## 1 Introduction

Poly(ethylene glycol) (PEG) has been the gold‐standard polymer in the fields of bioconjugation and nanomedicine for several decades, as it enhances the pharmacokinetic properties of a wide range of drugs. PEG is the most widely used polyether in pharmaceutical applications and cosmetics, comprising repeating ethylene glycol units (‐CH_2_‐CH_2_‐O‐). Pharma‐grade PEG is produced on an industrial scale via anionic ring‐opening polymerization (AROP) of ethylene oxide (EO).^[^ ^1^, ^2^ ^]^ Its excellent water solubility at physiologically relevant temperatures^[^ ^2^ ^]^ and excellent biocompatibility^[^ ^3^, ^4^, ^5^ ^]^ have made it widely applicable for pharmaceutical, medical and cosmetic applications. In the human body, PEG is primarily excreted via renal clearance up to a molecular weight of 30 kDa, while for larger PEGs, hepatic clearance or pinocytosis/phagocytosis is favored.^[^ ^6^, ^7^ ^]^ However, due to the high chemical stability of ether bonds, degradation of PEG has not been observed to any medically significant extent.^[^ ^8^ ^]^ Additionally, no pattern of adverse events indicative of potential PEG toxicity was detected in the past, even at exposure to ultrahigh doses of PEG in preclinical toxicology studies.^[^ ^8^, ^9^ ^]^ Therefore, PEG is classified as “non‐toxic” by the US Food and Drug Administration (FDA).^[^ ^10^ ^]^ The so‐called “PEGylation” strategy involves the covalent attachment of PEG to proteins, peptides, nanoparticles, and other molecules.^[^ ^11^ ^]^ It is widely employed to improve solubility, stability, and supposedly reduce the immunogenicity of the conjugated molecules. This so‐called “stealth effect” of PEG considerably prolongs the retention time of active pharmaceutical ingredients and nanocarriers in the bloodstream and has been fundamental in the development of numerous FDA‐approved therapies.^[^ ^12^, ^13^, ^14^ ^]^

However, recent literature has indicated that exposure to PEGylated pharmaceutical ingredients induces the formation of anti‐PEG antibodies (APAs), which have also been detected in healthy individuals with no previous medication including PEGylated drugs.^[^ ^15^, ^16^, ^17^, ^18^ ^]^ The presence of these antibodies can lead to accelerated blood clearance of PEGylated drugs, resulting in reduced drug efficacy, which is contrary to the intended purpose of PEGylation.^[^ ^19^, ^20^ ^]^ Furthermore, immune responses can trigger hypersensitivity reactions and severe side effects in a fraction of patients.^[^ ^21^, ^22^, ^23^, ^24^ ^]^ To counteract the potentially increasing issue of immunogenic reactions and other unclear side effects of PEG, it is crucial to develop alternatives for PEGylation.^[^ ^25^ ^]^ Several polymers have been developed as a non‐immunogenic alternative to PEG. These include poly(oxazolines) (POx),^[^ ^26^ ^]^ poly(vinylpyrrolidone) (PVP)^[^ ^26^, ^27^ ^]^ and polysarcosine (PSar),^[^ ^28^ ^]^ which have been investigated in recent years. Other variations of PEG architectures like branched PEGs have also been investigated and shown to reduce accelerated blood clearance in mice.^[^ ^29^ ^]^ Reduced immune response in mice has also been shown for bottlebrush polymers like poly(oligo(ethylene glycol) methyl ether methacrylate) (POEGMA).^[^ ^30^ ^]^ However, these polymers possess regular PEG segments like the aforementioned PEG alternatives and can therefore be subjected to immune adaptation. Statistical copolymers of ethylene oxide and propylene oxide generally show a significantly lower hydrophilicity than PEG.^[^ ^31^ ^]^

We have recently developed an approach for the synthesis of well‐defined, isomerized PEG structures via AROP, consisting of the building blocks EO and glycidyl methyl ether (GME). The GME units are randomly distributed along the polyether chains resulting in PEGs with “synthetic point mutations” – so‐called randomized PEG (rPEG).^[^ ^32^ ^]^ Contrary to the homopolymers investigated as PEG alternatives, the random sequence of comonomers in rPEGs could make adaptation of the immune system more difficult and potentially provide a long‐term solution to PEG immunogenicity. Our polymers show excellent aqueous solubility and – like conventional PEG – exhibited no apparent cytotoxicity, as demonstrated with a variety of murine and human cell lines. As recent work by Lai et al.^[^ ^33^ ^]^ shows, binding between PEG and the Fab‐fragment of the backbone‐specific PEG‐antibody is driven by multiple van der Waals interactions and requires a regular chain of 16 consecutive EO units. In our previously published work, we found indications in competitive ELISA (enzyme‐linked immunosorbent assays) that the randomly distributed side chains impede the polymer‐antibody interaction. However, the correlation between GME fraction –, i.e., the frequency of “point mutations” in a single rPEG chain – and antibody binding in solution remains to be elucidated.

Accordingly, we investigated the interaction between rPEGs and backbone‐specific APAs (immunoglobulin G). For this purpose, the copolymers were functionalized with a fluorescent dye, and their antibody interaction was examined by microscale thermophoresis (MST) and fluorescence correlation spectroscopy (FCS). Relying on these methods, correlation of the polymer structure with antibody binding affinities can be achieved. Furthermore, we aim to establish a quantification of the minimal fraction of GME required to sufficiently suppress antibody recognition. Additionally, the significance of the presence of 16 EO unit segments for antibody binding can be tested.

## 2 Results and Discussion

The polymers utilized in the MST and FCS investigations were synthesized via AROP under conditions like those employed for the production of pharma‐grade PEG. The synthesis of high purity GME can be found in our previous work.^[^ ^32^ ^]^ A well‐established route for the functionalization of polyethers was used to attach the fluorescent dye to the rPEGs as displayed in Scheme S1 (Supporting Information). To this end, a mesylate moiety was attached to the hydroxyl group of the polymer chains. Subsequently, the mesylate group was replaced by an azide moiety, which was eventually reduced to the amine.^[^ ^34^ ^]^ Finally, reaction with fluorescein‐5‐isothiocyanate (5‐FITC) was employed to produce the dye‐functionalized polymer from the amine. Characterization data of the functionalized polymers are given in **Table** **1**. Throughout this manuscript and the Supporting Information, the FITC functionalized samples´ composition is described with ${\mathrm{rPEG}}_{D_{p}}^{f}\;$where *f* is the molar fraction of GME and *D_p_* is the degree of polymerization of the sample.

**Table 1:** Analytical data of the functionalized polymers.

Polymer	mol%^GME^ (target)	mol%^GME^ ^a)^	*D*_p_ (target)	*D*_p_ ^b)^	*M*_n_ (target)/g mol^−1^	*M*_n_ ^c)^/ g mol^−1^	*M*_n_ ^b)^/g mol^−1^	*Đ* ^c)^
PEG_114_‐FITC	0	0	114	114	5405	4860	5300	1.06
${\mathrm{rPEG}}_{98}^{0.17}$‐FITC	15	17	98	98	5372	4170	5300	1.05
${\;\mathrm{rPEG}}_{89}^{0.27}$‐FITC	25	27	91	89	5378	4110	5200	1.12
${\;\mathrm{rPEG}}_{91}^{0.42}$‐FITC	40	42	81	91	5391	4440	6000	1.04
${\;\mathrm{rPEG}}_{76}^{0.52}$‐FITC	50	52	75	76	5374	3520	5400	1.04

^a)^calculated based on ^1^H‐NMR spectroscopy measurements.

^b)^calculated based on MALDI‐ToF measurements.

^c)^calculated based on GPC measurements.

**Figure 1:**
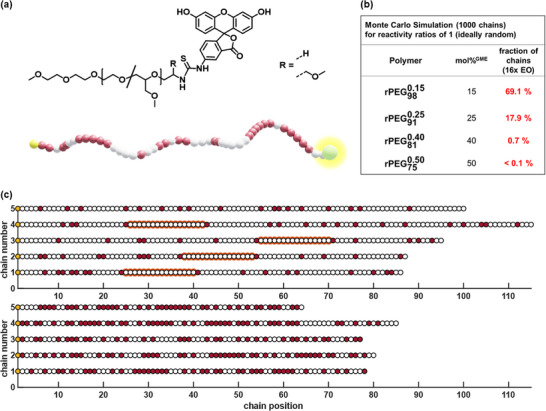
a) Structure and schematic depiction of the statistical EO/GME copolymers functionalized with FITC at the ω‐terminus. b) Evaluation (Monte‐Carlo Simulation) of the occurrence of the epitope consisting of 16 consecutive EO units in statistical copolymers of different compositions. c) Simulation of monomer sequences of copolymer chains containing 15 mol% GME (top) and 50 mol% GME (bottom), white = EO unit, red = GME unit, orange marking = sequences of ≥ 16 consecutive EO units.

Progress of the functionalization was monitored by ^1^H‐nuclear magnetic resonance (NMR) spectroscopy, gel permeation chromatography (GPC) and matrix‐assisted laser desorption ionization using time‐of‐flight (MALDI‐TOF) mass spectrometry. The general structure of the functionalized copolymers is illustrated in **Figure** **1**a. The molar amount of GME units in the copolymers can be determined via ^1^H‐NMR by calculating the relation between the signal of the methyl group of the GME side chain (3.30–3.35 ppm in CDCl_3_) and the signal of the methylene and methine groups of the backbone (3.37–4.00 ppm in CDCl_3_). Corresponding spectra as well as 2D diffusion‐ordered spectroscopy (DOSY)‐NMR spectra are shown in Figures s1–s31 (Supporting Information). To determine the average molar mass *M_n_*, MALDI‐TOF measurements were performed. Additionally, the mass of the polymer end group was determined using mass spectrometry. Mass spectra are provided in Figures S32–S56 (Supporting Information). This proved to be important to control the completion of the end group functionalization reactions, since in the ^1^H‐NMR spectrum characteristic signals of the end group partially overlapped with the backbone signal and therefore could not be integrated. The azide functionalization could only be monitored via ^1^H‐NMR by the disappearance of the mesylate signals of the precursor, since the signal of the azide‐attached methylene group overlaps completely with the polyether backbone signals. This observation aligns with reports in literature, which report the resonance at the same chemical shift as the signal of the GME methyl groups (3.30–3.35 ppm).^[^ ^35^, ^36^ ^]^ Finally, GPC was used to determine accurate values for the dispersities *Đ* of the polymer samples (see Figures S57–S61, Supporting Information).

As illustrated in Figure 1c, the results of the Monte Carlo simulation of copolymers with varying compositions reveal the reduced abundance of the epitope consisting of 16 consecutive EO units within the chains. This is evident in the simulation, which illustrates five random copolymer chains for molar GME contents of 15% and 50%. The data demonstrates a substantial decrease in the epitopal 16 EO units with increasing GME content, reaching a value below 0.1% at 50% GME content (Figure 1b,c).

**Figure 2:**
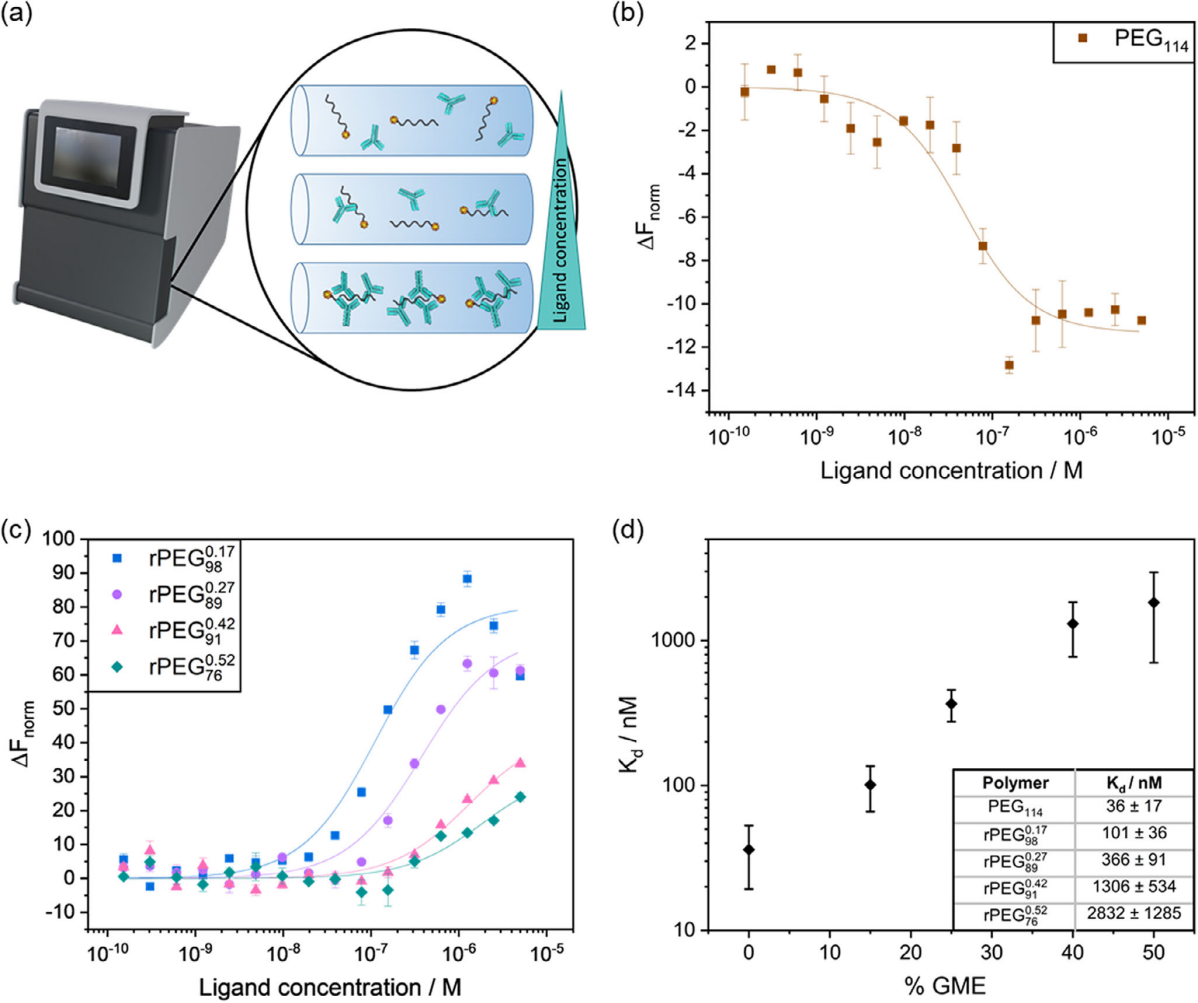
MST measurements of anti‐PEG IgG and PEG/rPEG. a) MST instrument and schematic representation of antibody binding in capillaries. b) Normalized change in fluorescence signal (Δ*F*_norm_) of anti‐PEG IgG binding to PEG together with the corresponding fit (mean ± S.D.). c) The normalized change in fluorescence signal of anti‐PEG IgG binding to rPEGs together with the corresponding fit (mean ± S.D.). d) Dissociation constant (*K*_d_) values for PEG/rPEGs plotted against increasing GME content in the PEG chain. Measurements were performed in triplicates.

MST measurements have been conducted to assess the interaction strength between APAs and free polymers. This technique relies on the temperature‐induced fluorescence change of a labeled target polymer to the concentration of a non‐fluorescent ligand. In this study, FITC‐conjugated rPEGs served as the target molecules. By systematically varying the GME amount in the PEG chain, it was possible to ascertain how the chain composition influences the binding affinity of the APAs, as reflected by the dissociation constant (*K_d_*). To ensure the validity of the experimental results, we utilized a FITC‐conjugated PEG with a respective molecular weight of 5000 g mol^−1^. The obtained results are displayed in **Figure** **2**.

The MST instrument's setup is illustrated in Figure 2a, and a schematic of the interaction of antibodies with the target polymer within the capillary is provided. The normalized change in fluorescence signal of APA binding to PEG (shown in Figure 2b) serves as a reference for comparison with modified rPEG derivatives displayed in Figure 2c showing the normalized change in fluorescence signal of APA binding to rPEGs containing varying concentrations of GME. As measurements were performed in triplicates, the average of n = 3 and corresponding standard deviation is shown (for individual measurements see Figure S62, Supporting Information).

The data demonstrate a decrease in the change in fluorescence with increasing GME content, along with a shift of the inflection points toward higher ligand concentrations, showing the correlation between higher GME content and reduced binding affinity. It is important to note that different target‐ligand complexes can produce thermophoretic responses in opposite directions, which is the reason for Δ*F*_norm_ < 0 in case of PEG and Δ*F*_norm_ > 0 in case of rPEGs. The amplitude and direction of the response depend on the specific molecular changes occurring upon binding, including modifications to size, charge, and hydration. This directional variability in thermophoretic response does not affect validity of binding strength comparisons between different target‐ligand pairs.

The graph in Figure 2d displays the obtained *K*_d_ values for PEG and rPEGs with different GME contents, revealing a strong increase in *K*_d_ values with higher GME content, which translates to a decrease in binding strength. For ${\mathrm{rPEG}}_{76}^{0.52}$ the substantial error bars and the nearly undetectable binding suggest minimal or no effective, specific binding interaction. For an effective binding detection, the signal‐to‐noise ratio needs to be above a threshold of 5 and ideally over 12, which was not achieved for all measurements with ${\mathrm{rPEG}}_{76}^{0.52}$. From Δ*F*_norm_ also the fraction of bound polymer can be plotted, which is shown in Figure S63 (Supporting Information) and supports the values obtained for *K*_d_. This underscores the impact of elevated GME content on the elimination of APA binding efficiency.

**Figure 3:**
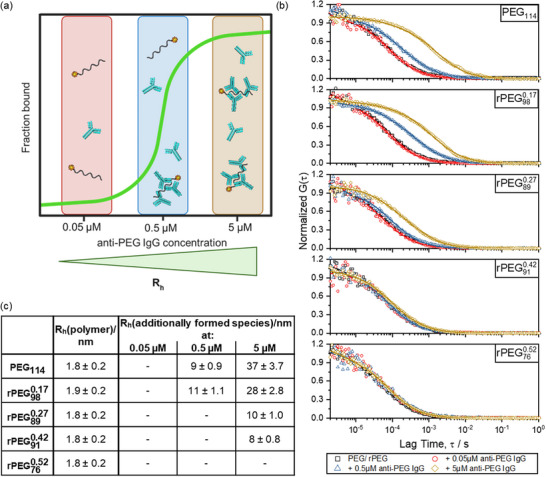
FCS measurements of anti‐PEG IgG and PEG/rPEG. a) Scheme of concentrations selected for the experiment and corresponding binding conditions according to the results obtained from the MST measurement. b) Autocorrelation functions (ACFs) derived from FCS measurements, shown together with the corresponding fits (single‐ or double‐component diffusion models). Black = individual PEG/rPEG polymers without anti‐PEG IgG addition, red = PEG/rPEG + 0.05 µm anti‐PEG IgG, blue = PEG/rPEG + 0.5 µm anti‐PEG IgG, yellow = PEG/rPEG + 5 µm anti‐PEG IgG. c) Table of hydrodynamic radii of detected diffusing species obtained from the fits shown in b). Error refers to the typical experimental error of ± 10% for FCS. Each FCS experiment included ten technical replicates per sample, acquired at 10s intervals.

The interaction between APAs and rPEG copolymers was further studied with FCS in order to obtain information about complex size, aggregation behavior, and interaction with other species.^[^ ^37^ ^]^ According to the results of the MST measurements, three concentrations of APAs were selected to represent unbound (0.05 µM), medium bound (0.5 µM), and fully bound (5 µM) conditions, as depicted in **Figure** **3**a. Typical FCS autocorrelation functions (ACFs) measured for fluorescently labeled PEG and rPEG copolymers in solutions with APAs with increasing concentrations are shown in Figure 3b.

ACFs acquired in the absence of antibodies are also included for reference. A shift of the ACF toward elevated lag times translates to an increase in the hydrodynamic radius (*R*_h_) of the fluorescent species, thereby reflecting enhanced interactions between APAs and polymers. The table in Figure 3c presents the hydrodynamic radii of the diffusing fluorescence species observed at different APA concentrations as obtained from fitting the corresponding ACF with single‐ or double‐component diffusion model (Equation 1 with *m* = 1 or 2). Consistently, the size of the non‐bound polymers without antibody presence was determined to be ≈1.8 nm, independent of the respective polymer's GME content. After mixing with anti‐PEG IgG, negligible interactions were observed at an antibody concentration of 0.05 µm for all polymers, as no size increase could be detected in any of the samples. This is expected, since this concentration is well below *K*_d_ for most of the polymers. At an anti‐PEG IgG concentration of 0.5 µm, PEG started to form aggregates (*R*_h_ = 9 nm) in addition to the presence of individual, free polymer chains that are still visible. Raising the concentration of antibodies to 5 µm, where the saturation of interaction should be reached, the aggregate size increased to 37 nm. A similar interaction pattern was observed for ${\mathrm{rPEG}}_{98}^{0.17}$. It is noteworthy that both PEG and ${\mathrm{rPEG}}_{98}^{0.17}$ exhibited substantial interactions at 0.5 and 5 µM, with a consistent increase in *R*_h_ with increasing antibody concentration. This observation aligns with the determined binding affinities, as a concentration of 0.5 µM is already above *K*_d_ for both polymers. However, for ${\mathrm{rPEG}}_{89}^{0.27}$, the anti‐PEG antibody binding effect was found to be reduced. At an anti‐PEG IgG concentration of 0.5 µM, negligible aggregate formation was observed (*R*_h_ = 2 nm), while at an anti‐PEG IgG concentration of 5 µM, an increase in aggregate formation is evident (*R*_h_ = 10 nm). This finding aligns with the observation that 0.5 µM anti‐PEG IgG falls within the *K_d_* range for ${\mathrm{rPEG}}_{89}^{0.27}$. In the case of ${\mathrm{rPEG}}_{91}^{0.42}$, negligible aggregation was observed, implying a lack of effective binding. Similarly, ${\mathrm{rPEG}}_{76}^{0.52}$ exhibited no measurable alterations in *R*_h_ even at high anti‐PEG IgG concentrations, indicating no discernible binding effects for ${\mathrm{rPEG}}_{91}^{0.42}$ and ${\mathrm{rPEG}}_{76}^{0.52}$.

These results collectively highlight the critical role of polymer composition in dictating antibody binding behavior. In particular, the lack of interaction seen with polymers ${\mathrm{rPEG}}_{91}^{0.42}$ and ${\mathrm{rPEG}}_{76}^{0.52}$ suggests that the high GME content within the polymer backbone substantially hinders antibody recognition and binding. To further verify compatibility with a physiological setting, FCS measurements of all polymers in human citrate plasma with average anti‐PEG IgG concentration (*c* = 9.6 ± 0.8 µg mL^−1^ corresponding to 0.064 µm, as reported by Deuker et al.^[^ ^16^ ^]^) were performed. The results are shown in Figure S64 (Supporting Information). For all polymers, no size increase was observed, indicating that no significant interactions with plasma components took place. However, it has to be noted that the detection of aggregates with anti‐PEG IgG was not expected at this APA concentration following the results reported in Figure 3, but might rather occur in samples collected from individuals with ≈ 10 times higher APA levels. Still, we can exclude any other undesired effects, suggesting safe application in biological environment. In the future, interactions need to be investigated under physiological conditions also at higher clinically relevant APA levels.

## 3 Conclusion

The binding of APAs to PEGylated drugs can present a significant problem, as it can induce the ABC effect or trigger immune reactions in patients. Consequently, alternatives to PEG are intensely discussed. In this study, we investigated the interaction between anti‐PEG IgG antibodies and a series of random copolymers of EO and GME (rPEG) in relation to the respective molar fraction of GME. It should be emphasized that any copolymer of EO and GME is a structural isomer of PEG, irrespective of its composition. The MST analysis revealed a decrease in the normalized fluorescence signal (*ΔF_norm_*) with increasing GME content in the rPEGs. Accordingly, the dissociation constants *K*_d_ of the copolymers increased analogously, revealing a decrease in the binding affinity of anti‐PEG IgG. Further studies relying on FCS demonstrated that polymer‐antibody aggregate formation depended on the GME content in the polymer. At an antibody concentration of 0.05 µM, no significant interaction was detected. At higher concentrations, an increase in the hydrodynamic radii *R_h_* was observed for all polymers, except for ${\mathrm{rPEG}}_{76}^{0.52}$. The extent of aggregation decreased as the GME content increased, confirming that GME units in the polymer chain impede antibody binding and suppress aggregate formation. Lai et al.^[^ ^33^ ^]^ found 16 consecutive EO units to be a prerequisite for backbone‐specific APA binding to the polymer. Our simulations demonstrate that this type of chain segment manifests less frequently in chains with higher molar GME content and is virtually absent in polymers exceeding 50 mol% GME. The experimental data aligns closely with the simulation outcomes, as evidenced by an analogous decrease in the interaction between APAs and rPEGs during MST measurements. Consequently, our results prove that EO‐GME copolymers possess the capacity to mitigate backbone‐specific APA interactions and that incorporating 50 mol% GME into the polymer chains effectively prevents the binding of anti‐PEG IgG antibodies. Since key properties such as aqueous solubility and cell viability of the polymer remain unaltered,^[^ ^32^ ^]^ rPEG can be regarded as a superior alternative to PEG. Furthermore, we postulate that due to the substantial variation in the statistical composition of the copolymer chains, the adaptation of the immune system and its interaction with rPEG could be significantly impeded compared to PEG. Of course, this hypothesis has to be examined through in vivo studies by repeated injections to investigate the effect of long‐term exposure. This will be the subject of future research. By understanding the polymer‐antibody interactions, we aim to contribute to the development of more effective and safer PEG alternatives, albeit based on PEG‐derived structures.

## 4 Experimental Section

### Materials

Ethylene oxide was purchased from *Air Liquide Deutschland*. All solvents were obtained from *Thermo Fisher Scientific*. Compounds were purchased from *TCI Chemicals, Sigma‐Aldrich, and Thermo Fisher Scientific*. All materials were used as received. Dulbecco's phosphate buffered saline (PBS, Thermo Fisher Scientific, USA). Chimeric human anti‐PEG IgG (clone no. c3.3 IgG, product no. 3.3‐PABG‐A) was purchased from IBMS Academia Sinica (Taipei, Taiwan) and used without further purification.

To obtain pooled plasma, blood was taken from 10 healthy donors after obtaining informed consent and pooled subsequently. All experiments containing human blood plasma from these donors were approved by the ethics committee of the Landesärztekammer Rheinland‐Pfalz, Mainz, Approval No. 837.439.12 (8540‐F). Accordingly, all experiments involving human material were performed in compliance with all relevant laws and guidelines.

After collection, the citrate plasma was centrifuged at 20,000 g for 1 h at room temperature to remove residual protein precipitates and stored at ‐80 °C until further use.

### Proton Nuclear Magnetic Resonance Spectroscopy (^1^H NMR)

^1^H NMR was conducted on a Bruker Avance II HD 400 (400 MHz, 25 °C) and analyzed using *MestReNova v14.2* by *Mestrelab Research SL*. Deuterated chloroform and deuterated water were purchased from *Deutero GmbH*.

### Gel permeation Chromatography (GPC)

Measurements were conducted using an Agilent 1100 series HPLC system, which included a degasser, isocratic pump (G1310A), autosampler (G1313A), column oven (G1316A), and detectors for refractive index (RI) (G1310A) and variable wavelength (VWD) (G1314A). Separations were carried out employing a four‐column set‐up (*MZ‐Analysentechnik GmbH*) connected sequentially:

- HEMA‐40 guard column (40 Å pore size, 10 µm particle size, 50 x 8.0 mm)
- HEMA‐40 analytical column (40 Å pore size, 10 µm particle size, 300 x 8.0 mm)
- HEMA‐100 analytical column (100 Å pore size, 10 µm particle size, 300 x 8.0 mm)
- HEMA‐300 analytical column (300 Å pore size, 10 µm particle size, 300 x 8.0 mm)

The eluent consisted of DMF (Fisher Chemical) with 1 mg mL^−1^ anhydrous LiBr (Acros Organics), using a flow rate of 1 mL min^−^^1^. Both the column oven and RI detector cell were maintained at 50 °C. Calibration was performed using well‐defined poly(ethylene glycol)s from PSS (PSS Standards Kit) with molar mass values (*M*_p_) ranging from 106 to 42 700 g mol^−1^. Samples were dissolved in DMF (with 1 mg mL^−1^ anhydrous LiBr) at a concentration of 1 mg mL^−1^ with the addition of 1 drop of toluene. The injection of 100 µL of the stock solutions was carried out via the autosampler, with a measurement duration of 45 min. Elution times were referenced using toluene as an internal standard. RI traces were analyzed using the *PSS WinGPC Unichrom V8.31* software.

### Matrix‐Assisted Laser Desorption Ionization Using Time‐of‐Flight Detection (MALDI‐TOF)

MALDI‐TOF MS measurements were carried out at *a Bruker autoflex maX MALDI‐TOF/TOF* using a Smartbeam‐II solid‐state laser with a wavelength of 337 nm. Spectra were recorded using the software *Bruker flexControl 3.4* and analyzed using *Bruker flexAnalysis 3.4* and *Bruker polytools 1.31*. The potassium salt of triflouroacetic acid (KTFA) and trans‐2‐[3‐(4‐tert‐Butylphenyl)‐2‐methyl‐2‐propenylidene]malononitrile (DCTB) were utilized as ionization salt and matrix, respectively. For sample preparation, the polymers were dissolved in chloroform at 10 mg mL^−1^. 20 µL of this solution was combined with 20 µL of a 10 mg mL^−1^ solution of the matrix in chloroform. 5 µL of a 0.1 m solution of the salt in methanol were added and 1 µL of the resulting mixture was spotted onto an *MTP 384 ground steel target plate*. The solvents were allowed to evaporate completely before the measurement.

In some cases, fragmentation reactions were observed in the MALDI‐TOF spectra. In the mass spectra of the azide‐functionalized polymers (Figures S34,S39,S44,S49, and S54, Supporting Information), fragmentation of nitrogen can be observed. This reaction during the measurement is also reported in literature.^[^ ^38^ ^]^ A so far not determined fragmentation reaction could be observed in particular spectra of the FITC‐functionalized polymers (Figures S41 and S51, Supporting Information). Although the exact reaction remains unknown, the functionalization of all polymers could be verified via 2D DOSY‐NMR (Figures S27–S31, Supporting Information) and the GPC curves of the FITC‐functionalized polymers (Figure S61, Supporting Information) showed no increase in dispersity. Despite several purification steps – including extraction, dialysis, and preparative column chromatography – trace amounts of unreacted FITC could be observed in the 2D DOSY‐NMR spectra (Figures S27–S31) and confirmed via FCS measurements. However, because of the significant difference in diffusion coefficients compared to the polymers, unreacted FITC was determined to not impede the FCS and MST measurements in any way.

### Microscale Thermophoresis (MST)

Solutions of labeled polymers (PEG‐FITC and rPEG‐FITC) were prepared with a concentration of 40 nm in PBS buffer. The stock solution of the anti‐PEG antibody (anti‐PEG IgG c3.3, *c* = 10 µM in PBS) was to produce a series of 16 1:1 dilutions with PBS buffer, producing antibody concentrations ranging from 0.306 nm to 10 µM. For the measurement, each ligand dilution was mixed with an equal volume of labeled polymer solution, resulting in a final polymer concentration of 20 nm and final ligand concentrations ranging from 0.153 nM to 5 µM. After 10 min incubation time, the samples were loaded into Monolith NT.115 premium‐coated capillaries (NanoTemper Technologies). MST was measured using a Monolith NT.115 instrument (NanoTemper Technologies) at room temperature, with a blue excitation laser (488 nm) and medium MST power. Data from three independent measurements were analyzed using the MO. Affinity Analysis Software version 2.2.4, utilizing the signal from an MST‐on time of 10 s for rPEG‐FITC and 1.5 s for PEG‐FITC. Dose‐response curves were generated by fitting the averaged normalized fluorescence change (Δ*F*_*norm*_) to yield the dissociation constants (*K*_d_ values).

### Fluorescence Correlation Spectroscopy (FCS)

The stock solution of the anti‐PEG antibody was diluted to three different concentrations with PBS buffer: 0.1 µm, 1 µm and undiluted. 20 µL of the labeled polymers (PEG‐FITC and rPEG‐FITC, 40 nM) were mixed with 20 µL of each anti‐PEG antibody concentration. For measurements in human plasma, the labeled polymers (FITC‐PEG and FITC‐rPEG) were dissolved at a concentration of 200 nM in PBS. These polymer solutions were then mixed with human citrate plasma in a 1:10 ratio, resulting in a final polymer concentration of 20 nM.

FCS experiments were performed on a commercial device, LSM 880 (Carl Zeiss, Jena, Germany). The excitation was done with the 488 nm line of an Argon laser focused into the studied samples through a C‐Apochromat 40×/1.2 W water immersion objective (Carl Zeiss, Jena, Germany). The emission light was collected with the same objective and after passing through a confocal pinhole, directed to a spectral detection unit (Quasar, Carl Zeiss) in which a detection range of 500–550 nm was selected. Eight‐well polystyrene chambered cover glasses (Nunc™ Lab‐Tek™, Thermo Fisher Scientific, Waltham, MA, USA) were used as sample cells for the studied solutions. The confocal volume was positioned 20 µm above the cover glass. Each FCS experiment included ten technical replicates per sample, acquired at 10s intervals. All replicates were successful and showed consistent diffusion times and hydrodynamic radius values.

The recorded autocorrelation functions were fitted with the analytical expression for an ensemble of *m* different types of freely diffusing fluorescence species:^[^ ^39^ ^]^

(1) $$G\left(\tau \right)=1+\left[1+\frac{f_{T}}{1-f_{T}}e^{-\tau /\tau_{T}}\right]\frac{1}{N}\sum_{i=1}^{m}\frac{f_{i}}{\left[1+\frac{\tau }{\tau_{Di}}\right]\sqrt{1+\frac{\tau }{S^{2}\tau_{Di}}}}$$

Here, *N* is the average number of diffusing fluorescence species in the observation volume, *f_T_* and *τ_T_* are the fraction and the decay time of the triplet state, *τ_Di_* is the diffusion time of the *i‐th* type of species, *f_i_* is the fraction of component *i*, and *S* is the so‐called structure parameter, *S* = *z*_0_/*r*_0_, where *z*_0_ and *r*_0_ represent the axial and radial dimensions of the confocal volume, respectively. Furthermore, the diffusion time, *τ_Di_*, is related to the respective diffusion coefficient, *D_i_*, through: $\tau_{Di}=\frac{r_{0}^{2}}{4D_{i}}$. The fits yielded the corresponding diffusion times, and subsequently the diffusion coefficients of the fluorescent species. As the value of *r*_0_ depends strongly on the specific characteristics of the optical setup, calibration experiments were performed using a fluorescent tracer with a known diffusion coefficient, i.e., Alexa Fluor 488 in water. Finally, the hydrodynamic radii of the studied fluorescent species were evaluated from the respective diffusion coefficients using the Stokes‐Einstein (SE) relation:

(2) $$R_{h}=\frac{k_{B}T}{6\pi \eta D}$$

Here, *k_B_* is Boltzmann's constant, *T* is the temperature and *η* is the viscosity of water.

Analysis of microstructure based on simulations: Simulation of the comonomer sequence is based on the code written by Philip Dreier for our previous work.^[^ ^32^ ^]^ There, polymers of constant chain lengths were investigated. To accommodate for the varying chain lengths of the polymers investigated in this work, calculations were adapted accordingly.

In brief, the rationale of a living anionic polymerization was taken as a foundation which implies the absence of chain termination or chain transfer reactions and a Poisson distribution of the chain lengths. The copolymerization reaction can be described by the four rate constants: The homo‐propagation rate constants *k_11_* and *k_22_* as well as the cross‐propagation rate constants *k_12_* and *k_21_*. The copolymerization kinetics is known to be perfectly random,^[^ ^32^ ^]^ implying

(3) $$k_{11}=k_{22}=k_{12}=k_{21}$$

As a result, it was assumed in the simulations that EO and GME monomers possess the same probability to be incorporated into the chain. For each polymerization, 10^4^ chains were simulated. The simulations were run with a targeted quantitative conversion and an overall degree of polymerization of 114. Molar ratios of 15 up to 50% of GME with a stepwise increase were presumed. In Figure 1b of the main manuscript the four simulated polymerizations and the respective proportional occurrence of 16 consecutive EO units are listed. To visualize the results of the simulations, 5 exemplary chains each for GME contents of 15 mol% and 50 mol% are shown in Figure 1c of the main manuscript. All simulations were performed using *MathWorks Matlab R2019a*.

### Synthesis of Random Copolymers of Ethylene Oxide (EO) and Glycidyl Methyl Ether (GME)

The statistical copolymerization of EO and GME was performed as described in the literature.^[^ ^32^ ^]^ The synthesis is given exemplary for ${\mathrm{rPEG}}_{89}^{0.27}$ with a molecular weight of 5 kDa. All other polymer synthesis were performed identically with varying monomer equivalents. GME was freshly dried before every polymerization by stirring over calcium hydride overnight in a flame‐dried flask and subsequently cryo‐transferred immediately before use. Diethylene glycol monomethyl ether (34.1 mg, 280 µmol, 1 eq) was dissolved in benzene (5 mL) and transferred via syringe into a flame‐dried flask equipped with a stirrer bar, stop cock, and septum. Potassium *tert*‐butoxide (28.7 mg, 260 µmol, 0.9 eq) was dissolved in freshly distilled THF (3 mL) and added to the solution in the flask via syringe. The mixture was stirred for 20 min before the solvents were slowly removed under vacuum. The resulting initiator salt was dried at 60 °C under high vacuum overnight. The flask was sealed and the initiator salt was dissolved in dry DMSO (5 mL). The flask was subsequently cooled to −90 °C with an ethanol/nitrogen cooling bath. GME (555 mg, 6.30 mmol, 23 eq) was added via syringe into the cooled flask. Ethylene oxide (800 µL, 18.9 mmol, 67 eq) was condensed into a graduated ampule at −90 °C and then transferred to the flask. After sealing the flask, the cooling bath was removed and the mixture was allowed to reach room temperature. After stirring for 48 h at room temperature the polymerization was terminated by flushing the flask with argon and adding a mixture of 1 m HCl and methanol (1:1, 1 mL). The reaction mixture was taken up in chloroform (20 mL) and extracted twice with deionized water and subsequently with brine. The organic phase was dried with magnesium sulfate, filtered, and the solvent was removed under reduced pressure. The polymer was dialyzed against methanol (regenerated cellulose, MWCO = 2000 g/mol) for 24 h, affording a pale‐yellow viscous liquid (836 mg, 60%).

### Mesylation

The synthesized copolymer (800 mg, 160 µmol, 1 eq) was dissolved in benzene (5 mL) and transferred into a flame‐dried Schlenk flask under argon. Benzene was slowly removed under reduced pressure to azeotropically dry the polymer. Subsequently, the polymer was further dried at 60 °C under high vacuum overnight. After the flask was cooled down to room temperature the polymer was dissolved in dry dichloromethane (DCM) (10 mL) under an argon atmosphere. Triethylamine (130 mg, 177 µL, 1.28 mmol, 8 eq) was added and the solution was stirred for 10 min before methyl sulfonyl chloride (147 mg, 99.0 µL, 1.28 mmol, 8 eq) was added to the solution. The reaction mixture was stirred under argon atmosphere for 72 h at room temperature. Subsequently, the mixture was transferred to a separating funnel and washed two times with deionized water and once with brine, dried over magnesium sulfate, and filtered. After removal of the solvent 649 mg (81%) of mesylated polymer was isolated.

### Azidation

The mesylated polymer (600 mg, 120 µmol, 1 eq) was dissolved in benzene (5 mL) and transferred into a flame‐dried Schlenk flask under argon. Benzene was slowly removed under reduced pressure, and the polymer was dried at 60 °C under high vacuum overnight. After removing the heating bath, the polymer was dissolved in 5 mL of dry DMF under argon atmosphere. Sodium azide (62.4 mg, 960 µmol, 8 eq) was added under argon flow, and the mixture was stirred at 65 °C for 72 h under argon atmosphere. Subsequently, DMF was removed under reduced pressure, the residue taken up in DCM (5 mL), and the precipitate was removed by filtration. The remaining solution of the polymer in DCM was washed once with deionized water and once with brine, dried over magnesium sulfate, and the solvent was removed under vacuum to obtain 390 mg (64%) of polymer azide.

### Reduction of the Azide

The polymer azide (300 mg, 60 µmol, 1 eq) was dissolved in ethanol (5 mL) and transferred into a Schlenk flask. 10 w% Pd/C (39.4 mg, 40 µmol Pd, 0.66 eq) were suspended in 2 mL of ethanol and added to the polymer solution. The flask was sealed and a vacuum was applied until the solvent started to evaporate. The flask was flooded with argon again and this procedure was repeated two more times. Afterward, a balloon filled with hydrogen gas was connected to the flask, and a vacuum was applied again until the solvent started to evaporate. This time the flask was flooded with hydrogen from the balloon. This procedure was also repeated two more times so the atmosphere in the flask was saturated with hydrogen gas. The mixture was stirred under hydrogen atmosphere for 48 h and the hydrogen balloon was refilled after 24 h as needed. The reaction mixture was subsequently filtered over Celite, the filter was washed twice with ethanol, and the solvent was removed under reduced pressure to afford 277 mg (92%) of polymer amine.

### Functionalization with FITC

A carbonate‐buffer solution with a concentration of 0.1 mol L^−1^ at pH10 was prepared by dissolving sodium bicarbonate (3.88 g, 46 mmol) and sodium carbonate (5.71 g, 54 mmol) in Millipore water (1000 mL). The polymer amine (100 mg, 20 µmol, 1 eq) and fluorescein isothiocyanate (5‐FITC, 15.6 mg, 40 µmol, 2 eq) were dissolved separately in the buffer solution (1.5 mL). Both solutions were combined and kept at 4 °C for 120 h under exclusion of light. Afterward, the reaction mixture was transferred into a tainted glass flask and water was removed under reduced pressure. The residue was taken up in DCM (5 mL) and the precipitate was filtered off. The solution was washed once with the buffer solution and then with brine, dried over magnesium sulfate, and the solvent was removed again. The resulting polymer was dialyzed against the carbonate buffer solution (regenerated cellulose, MWCO = 2000 g mol^−1^) under the exclusion of light. Finally, the dialyzed polymer solution was transferred to a tainted glass flask and the water was removed again under reduced pressure. The residue was taken up in chloroform (2 mL) and the precipitate was removed by filtration. The solution of the functionalized polymer in chloroform was purified via preparative GPC to yield 32 mg (30%) of FITC‐functionalized polymer.

## Conflict of Interest

The authors declare no conflict of interest.

## Supporting information



Supporting Information

## Data Availability

The data that support the findings of this study are available from the corresponding author upon reasonable request.
