# Supplementary material for: Overcoming PEG Antigenicity: Statistical PEG Isomers Reduce Antibody Binding
Source: Adv Sci (Weinh). 2025 Nov 19;13(7):e21061. doi: 10.1002/advs.202521061 (PMC12866791; doi:10.1002/advs.202521061)
Supplement: Supplementary file 1 — Supporting Information [file ADVS-13-e21061-s001.docx]

**Supporting Information**

**Overcoming PEG Antigenicity: Statistical PEG Isomers Reduce Antibody Binding**

Mareike Deuker^[a]^, Dominik Schulz^[b]^, Kaloian Koynov^[a]^, Svenja Morsbach*^[a]^, Holger Frey*^[b]^, Katharina Landfester*^[a]^

^[a]^ Max Planck Institute for Polymer Research, Ackermannweg 10, 55128 Mainz, Germany

^[b]^ Johannes Gutenberg-University, Department of Chemistry, Duesbergweg 10-14, 55128 Mainz, Germany

**Table of Content**

1. **Reaction sequence: copolymer functionalization p. 2**
2. **Proton nuclear magnetic resonance (^1^H-NMR) spectra p. 3 – 16**
3. **2D DOSY NMR spectra p. 16 – 18**
4. **Matrix-assisted laser desorption ionization using time-of-flight (MALDI-TOF) mass spectra p. 19 – 31**
5. **Gel permeation chromatography (GPC) curves p. 31 – 33**
6. **Microscale thermophoresis (MST) curves p. 34 – 35**
7. **Fluorescence Correlation Spectroscopy (FCS) measurements in human plasma p. 36**

**1. Reaction sequence: copolymer functionalization**

*Scheme S 1: Reaction sequence: functionalization of the copolymers.*

**n/m**

**n/m**

**n/m**

**n/m**

**n/m**

**2. Proton nuclear magnetic resonance (^1^H-NMR) spectra**

The last repeating unit of the polymer chain is statistically either an EO unit (R = -H) or a GME unit (R = -CH_2_-O-CH_3_). This difference is visible in the spectra of the mesylated rPEGs, where distinct signals for the terminal methylene group of the EO unit and the methine group of the GME unit can be observed. In all other cases the signal of the methine group overlaps with the backbone signal and therefore is not visible in the spectrum.

The samples´ composition is described with $\text{rPEG}_{\text{D}_{\text{p}}}^{\text{f}}$where *f* is the molar fraction of GME and *D_p_* is the overall degree of polymerization of the sample.

Figure S 1: ^1^H-NMR spectrum (CDCl_3_, 400 MHz) of glycidyl methyl ether (GME).


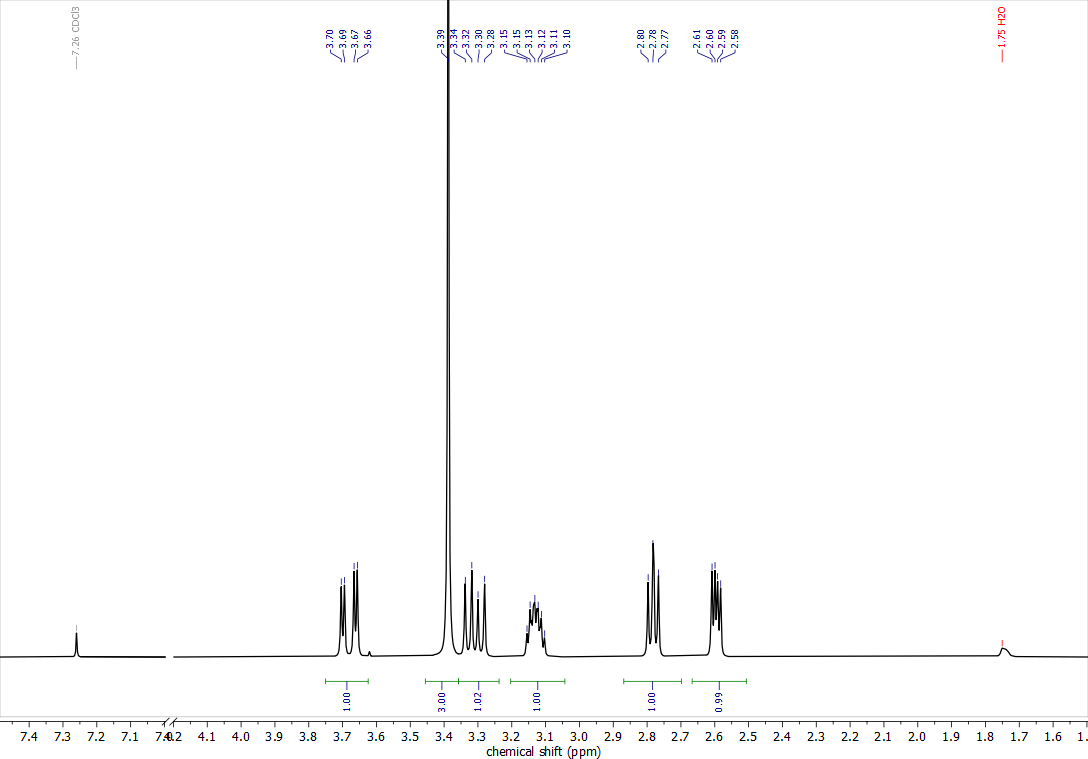

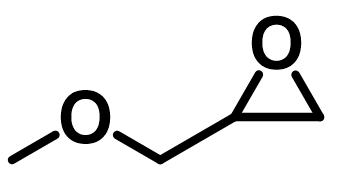


***b***

***a***

***a***

***c***

***d***

***b‘***

***c***

***d‘***

***d‘‘***

***b‘‘***


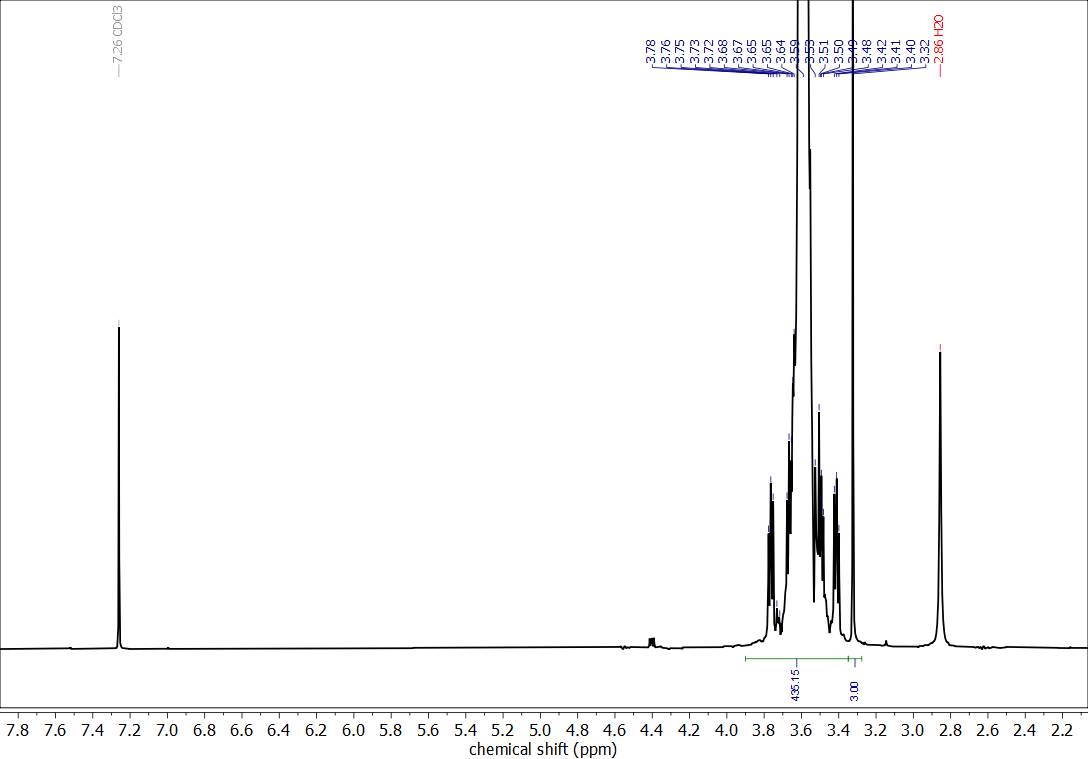


***b***

***a***

***a***

***b***

***b***

***b***

***b***

***b***

***b***

***b***

***b***

**n**

*Figure S 2: ^1^H-NMR spectrum (CDCl_3_, 400 MHz) of* $\text{mPEG}_{\text{114}}$*.*

*Figure S 3: ^1^H-NMR spectrum (CDCl_3_, 400 MHz) of* $\text{rPEG}_{\text{98}}^{\text{0.17}}$*.*


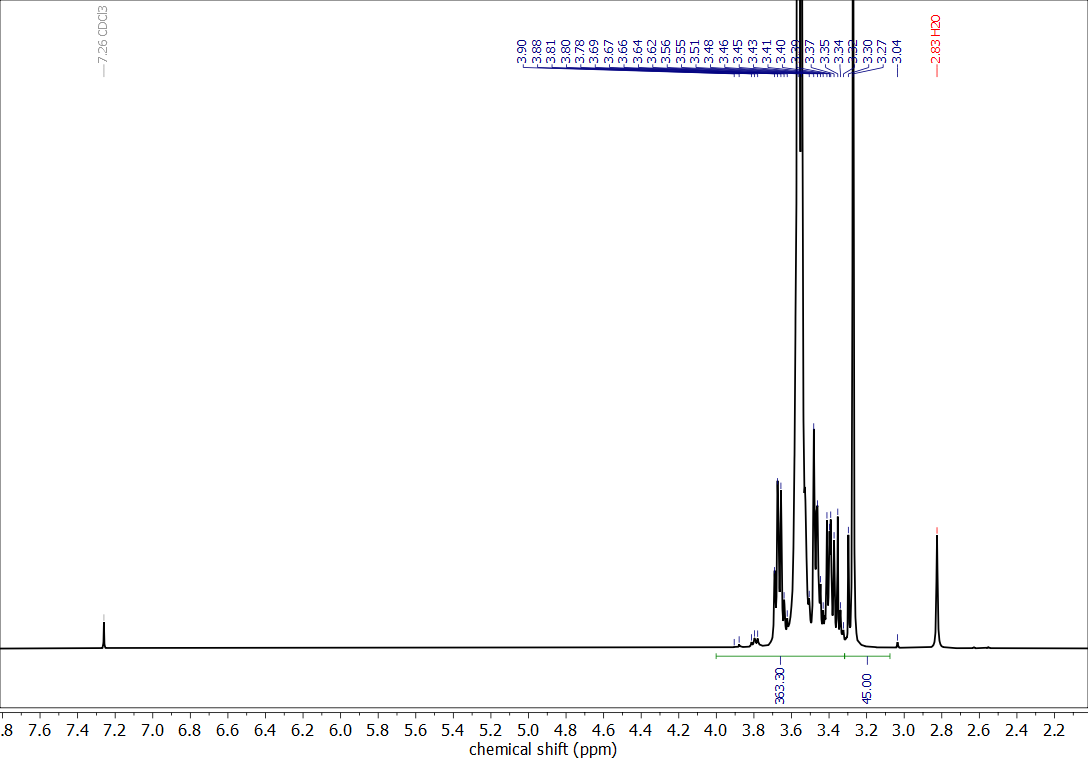


***b***

***a***

***b***

***a***

***b***

***b***

***b***

***b***

***b***

***b***

***b***

***b***

***b***

***a***

***b***

**n/m**

Figure S 4: ^1^H-NMR spectrum (CDCl_3_, 400 MHz) of $\text{rPEG}_{\text{89}}^{\text{0.27}}$.


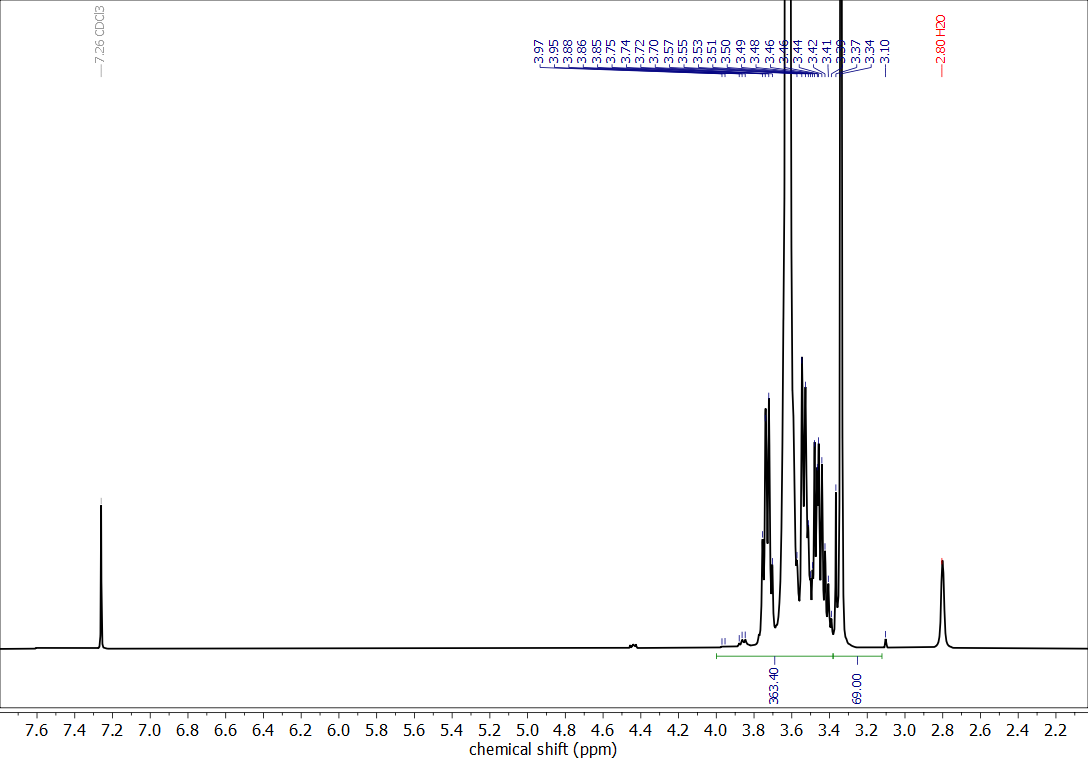


***b***

***a***

***b***

***a***

***b***

***b***

***b***

***b***

***b***

***b***

***b***

***b***

***b***

***a***

***b***

**n/m**

*Figure S 5: ^1^H-NMR spectrum (CDCl_3_, 400 MHz) of* $\text{rPEG}_{\text{91}}^{\text{0.42}}$*.*


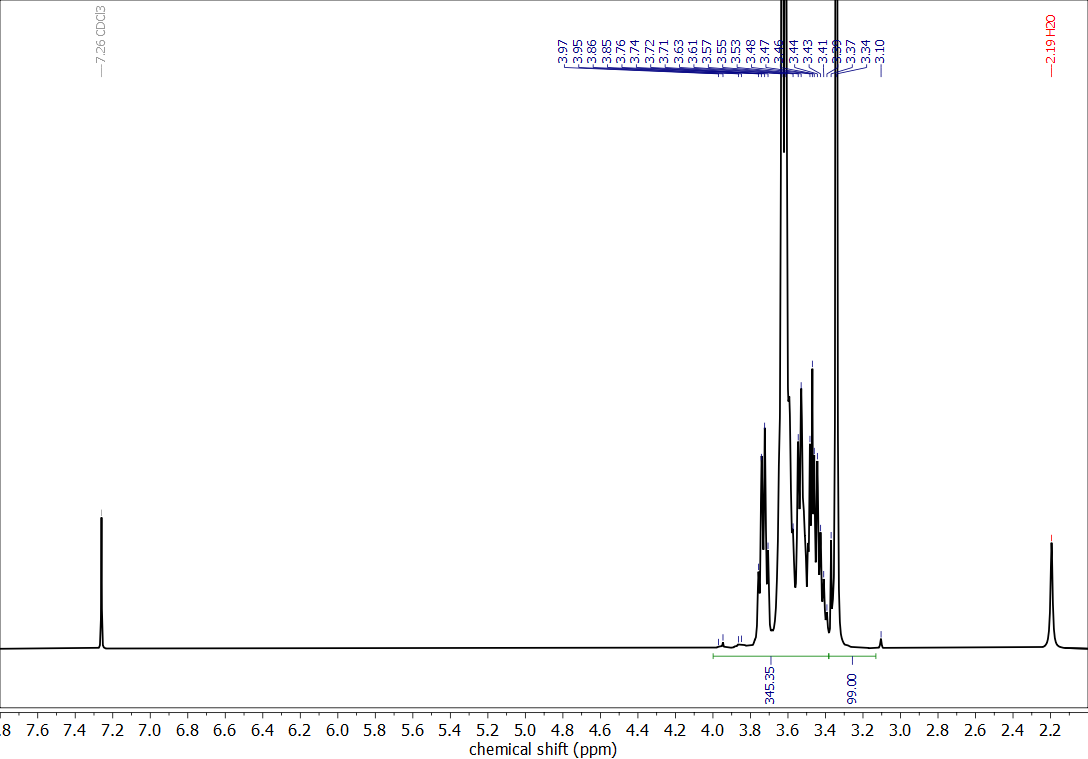


***b***

***a***

***b***

***a***

***b***

***b***

***b***

***b***

***b***

***b***

***b***

***b***

***b***

***a***

***b***

**n/m**

*Figure S 6: ^1^H-NMR spectrum (CDCl_3_, 400 MHz) of* $\text{rPEG}_{\text{76}}^{\text{0.52}}$*.*


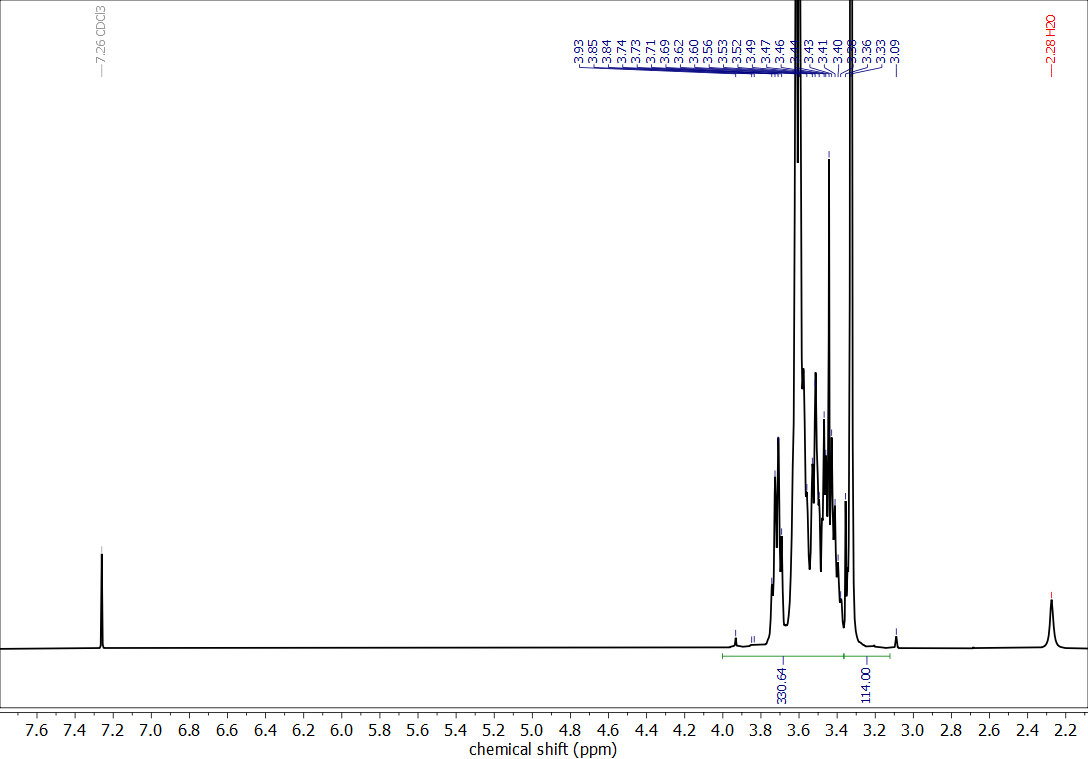


***b***

***a***

***b***

***a***

***b***

***b***

***b***

***b***

***b***

***b***

***b***

***b***

***b***

***a***

***b***

**n/m**

Figure S 7: ^1^H-NMR spectrum (CDCl_3_, 400 MHz) of $\text{mPEG}_{\text{114}}$-mesylate.


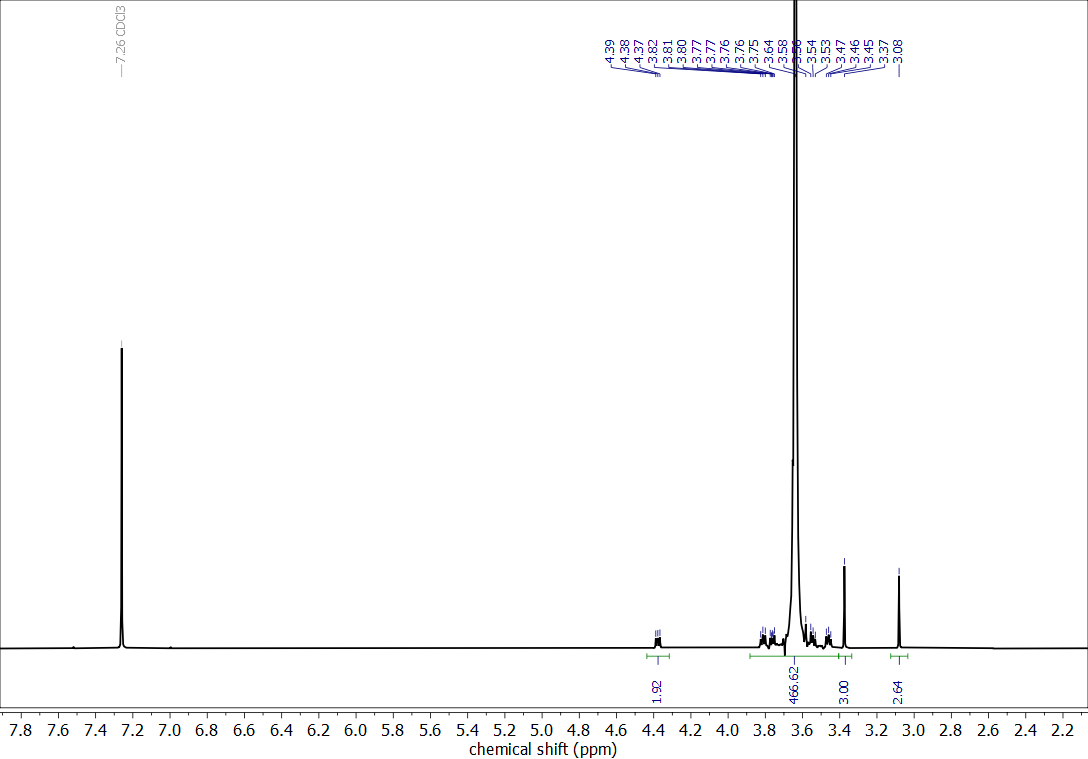


***b***

***a***

***c***

***d***

***a***

***b***

***b***

***b***

***b***

***b***

***b***

***d***

***b***

***c***

**n**

*Figure S 8: ^1^H-NMR spectrum (CDCl_3_, 400 MHz) of* $\text{rPEG}_{\text{98}}^{\text{0.17}}$*-mesylate*

*(c/d = terminal EO unit, c’/d’ = terminal GME unit).*


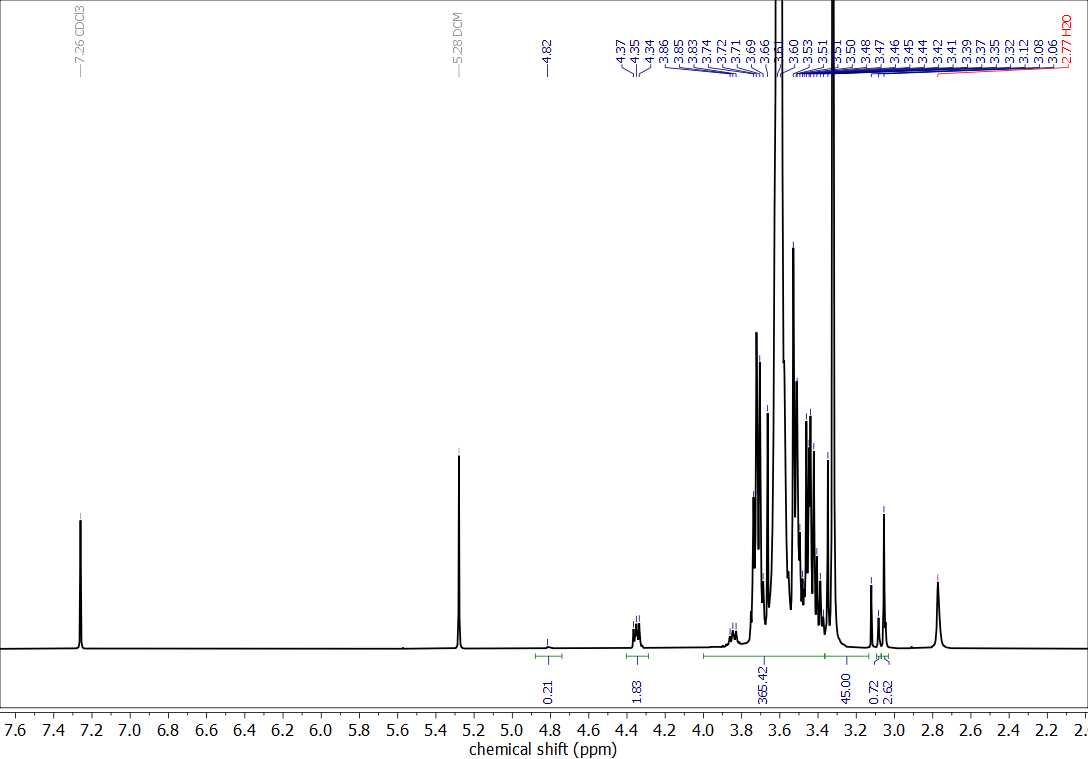


***b***

***a***

***b***

***b***

***b***

***b***

***b***

***b***

***c***

***b***

***b***

***a***

***b***

***d***

**n/m**

***b***

***a***

***c***

***d***

***d‘***

***c‘***

*Figure S 9: ^1^H-NMR spectrum (CDCl_3_, 400 MHz) of* $\text{rPEG}_{\text{89}}^{\text{0.27}}$*-mesylate*

*(c/d = terminal EO unit, c’/d’ = terminal GME unit).*


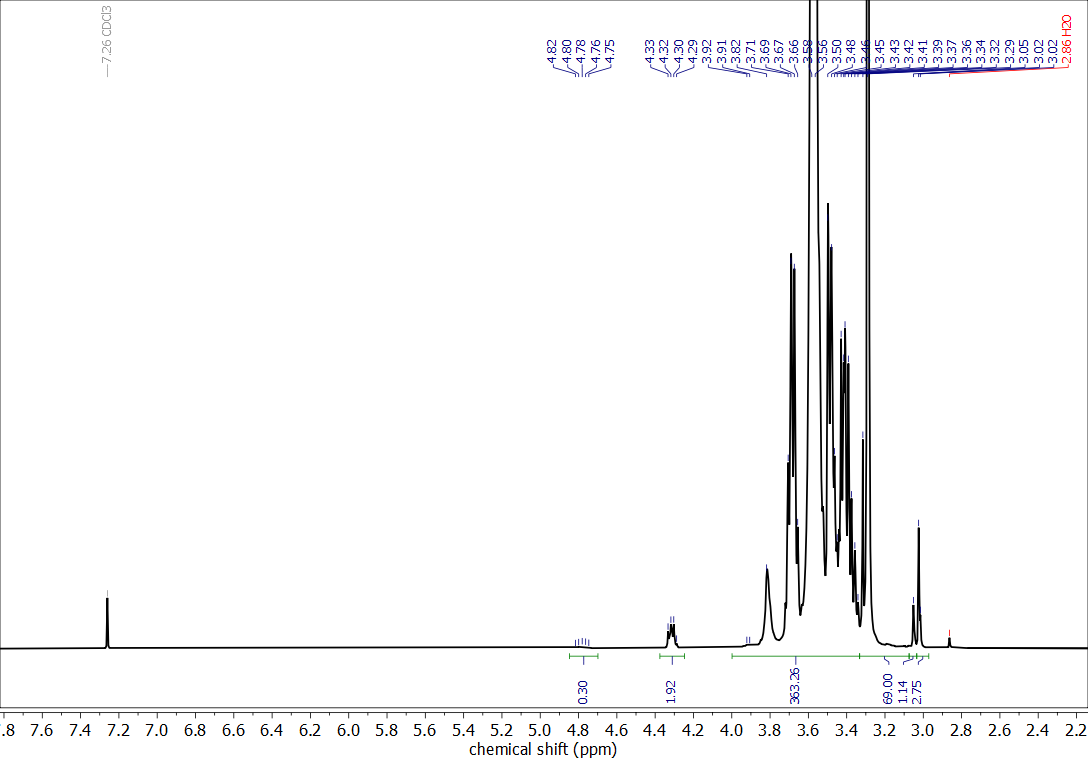


***b***

***a***

***b***

***b***

***b***

***b***

***b***

***b***

***c***

***b***

***b***

***a***

***b***

***d***

**n/m**

***b***

***a***

***c***

***d***

***d‘***

***c‘***

*Figure S 10: ^1^H-NMR spectrum (CDCl_3_, 400 MHz) of* $\text{rPEG}_{\text{91}}^{\text{0.42}}$*-mesylate*

*(c/d = terminal EO unit, c’/d’ = terminal GME unit).*


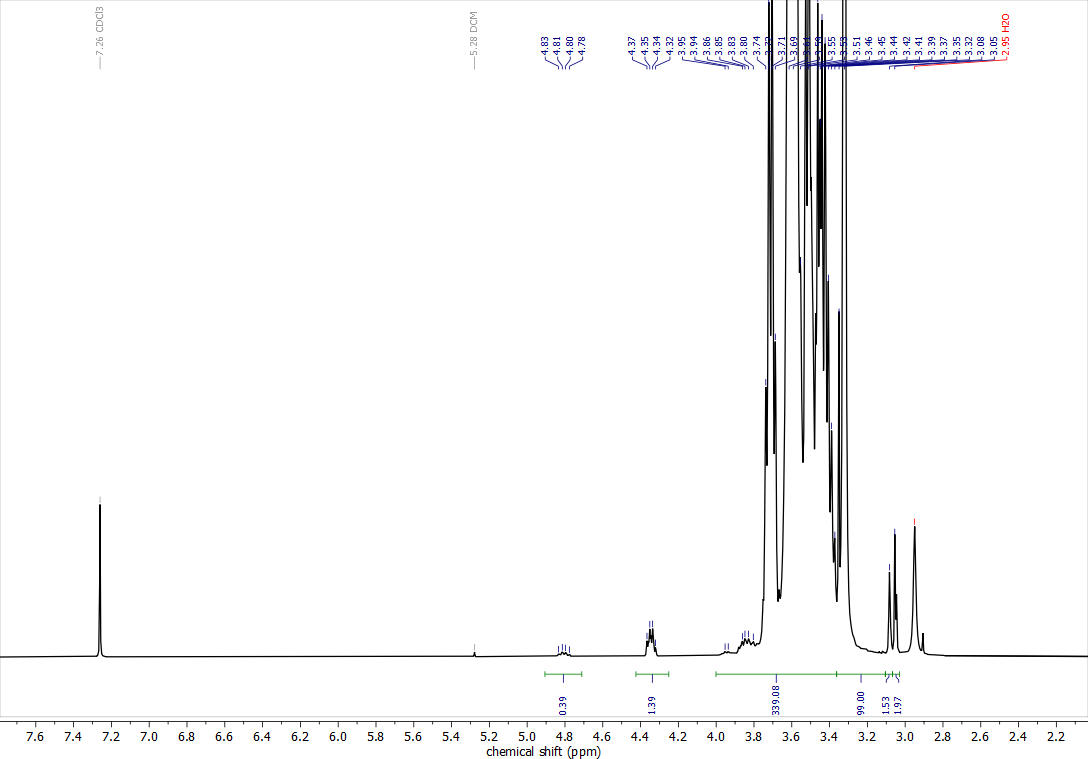


***b***

***a***

***c***

***d***

***d‘***

***c‘***

***b***

***a***

***b***

***b***

***b***

***b***

***b***

***b***

***c***

***b***

***b***

***a***

***b***

***d***

**n/m**

*Figure S 11: ^1^H-NMR spectrum (CDCl_3_, 400 MHz) of* $\text{rPEG}_{\text{76}}^{\text{0.52}}$*-mesylate*

*(c/d = terminal EO unit, c’/d’ = terminal GME unit).*


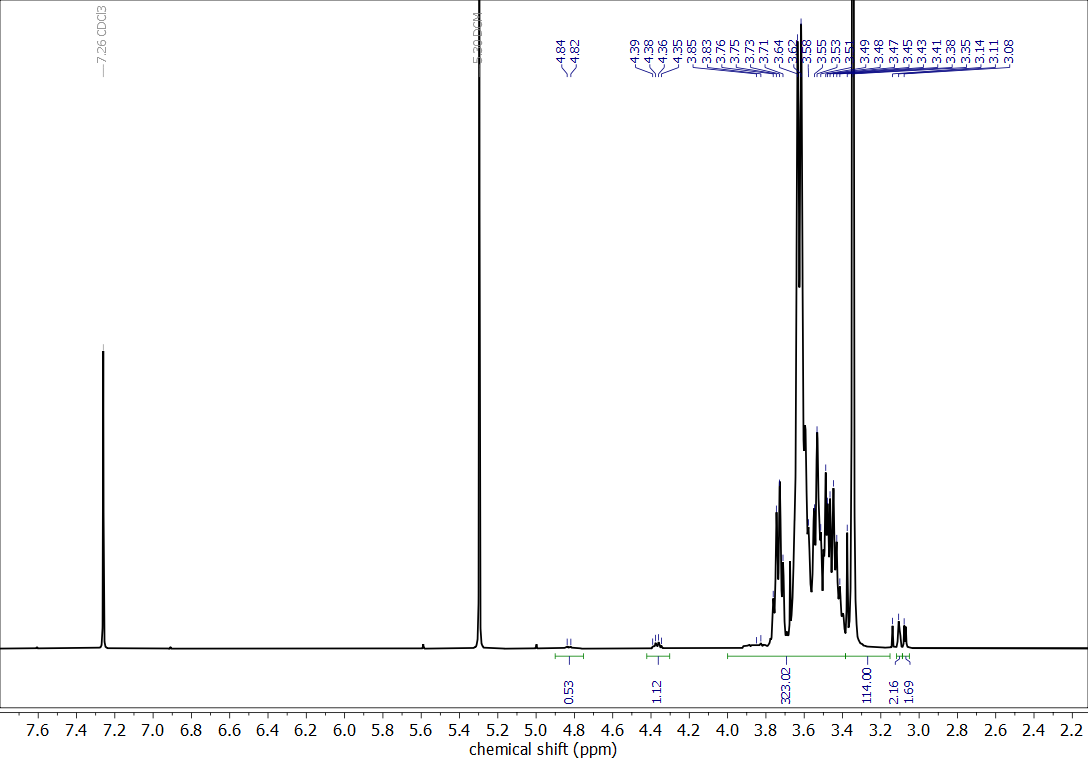


***b***

***a***

***c***

***d***

***d‘***

***c‘***

***b***

***a***

***b***

***b***

***b***

***b***

***b***

***b***

***c***

***b***

***b***

***a***

***b***

***d***

**n/m**

Figure S 12: ^1^H-NMR spectrum (CDCl_3_, 400 MHz) of $\text{mPEG}_{\text{114}}$-azide.


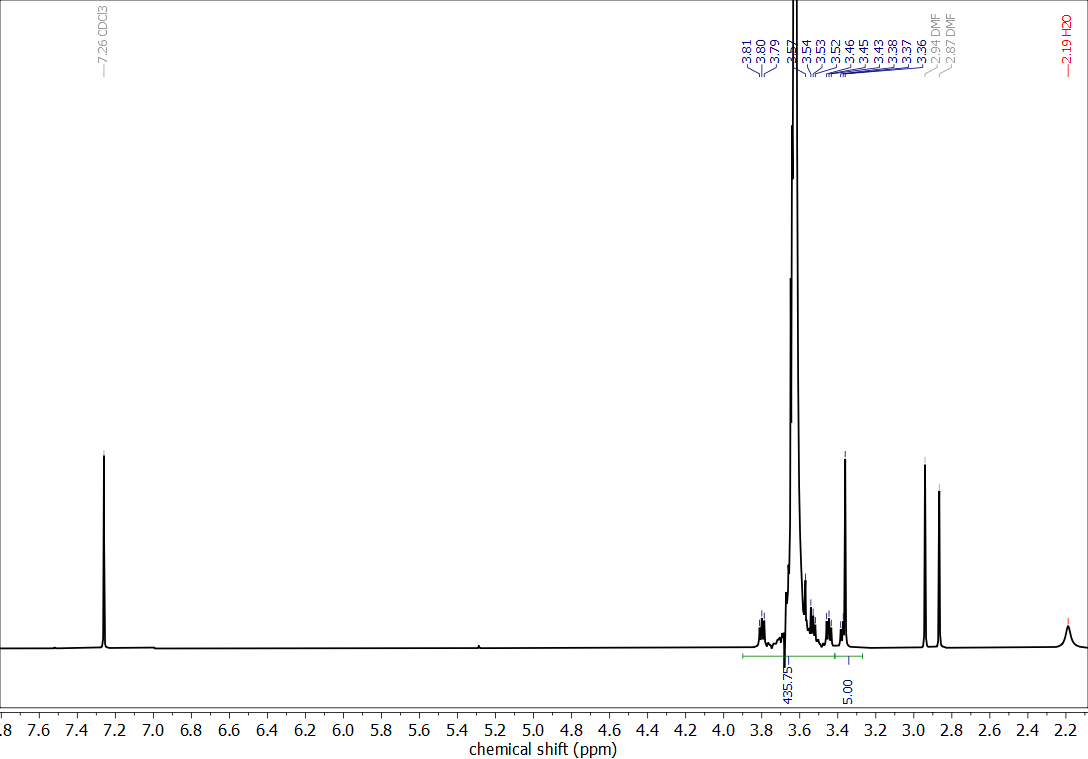


***a***

***b***

***b***

***b***

***b***

***b***

***b***

***b***

***c***

**n**

***b***

***a, c***

*Figure S 13: ^1^H-NMR spectrum (CDCl_3_, 400 MHz) of* $\text{rPEG}_{\text{98}}^{\text{0.17}}$*-azide*

*(c = terminal EO unit, c’ = terminal GME unit).*


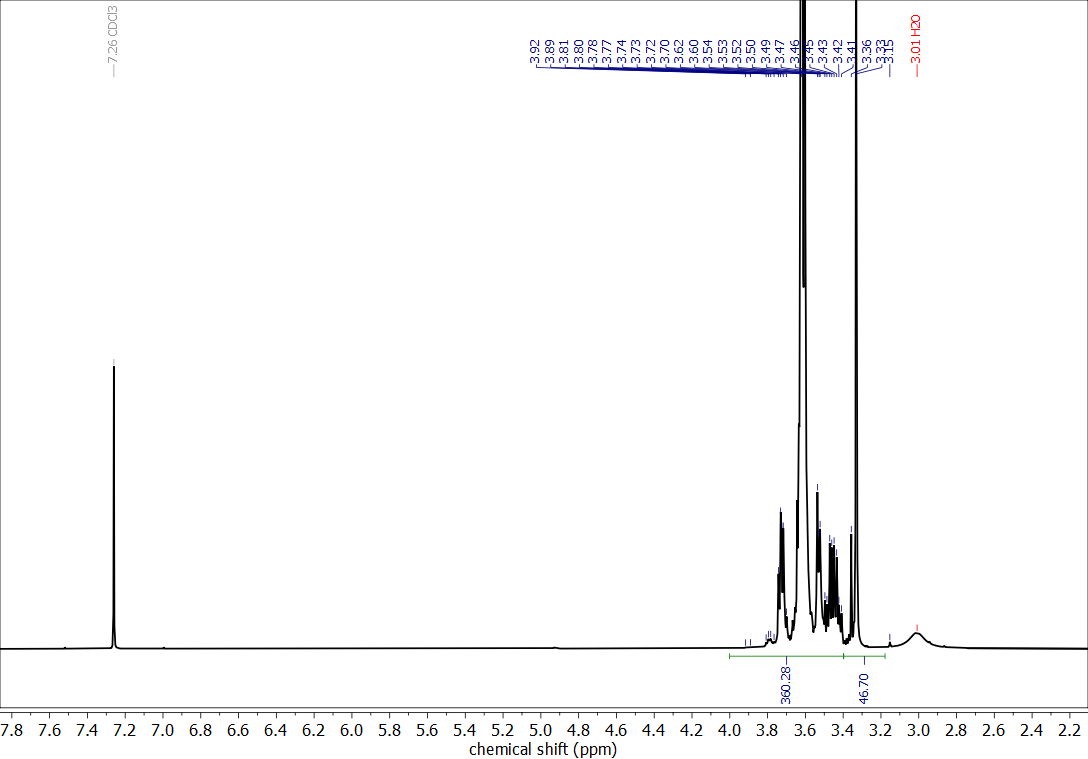


***b***

***a***

***b***

***b***

***b***

***b***

***b***

***b***

***c***

***b***

***b***

***a***

***b***

**n/m**

***b, c‘***

***a, c***

*Figure S 14: ^1^H-NMR spectrum (CDCl_3_, 400 MHz) of* $\text{rPEG}_{\text{89}}^{\text{0.27}}$*-azide*

*(c = terminal EO unit, c’ = terminal GME unit).*


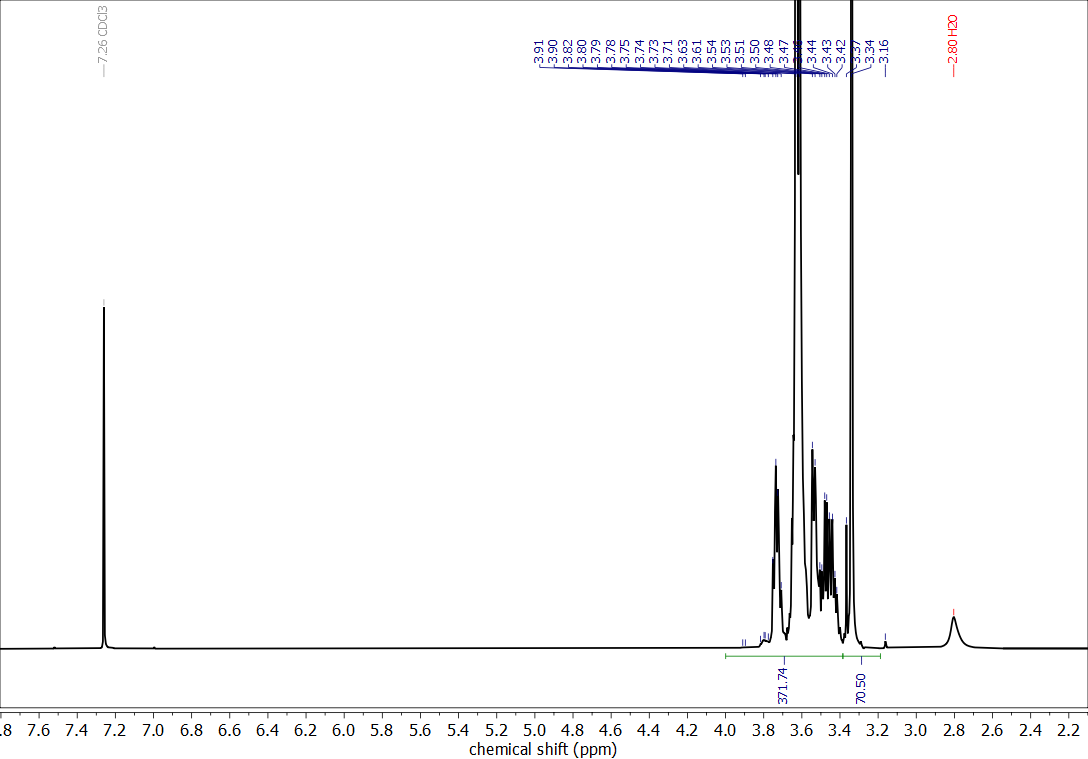


***b***

***a***

***b***

***b***

***b***

***b***

***b***

***b***

***c***

***b***

***b***

***a***

***b***

**n/m**

***b, c‘***

***a, c***

*Figure S 15: ^1^H-NMR spectrum (CDCl_3_, 400 MHz) of* $\text{rPEG}_{\text{91}}^{\text{0.42}}$*-azide*

*(c = terminal EO unit, c’ = terminal GME unit).*


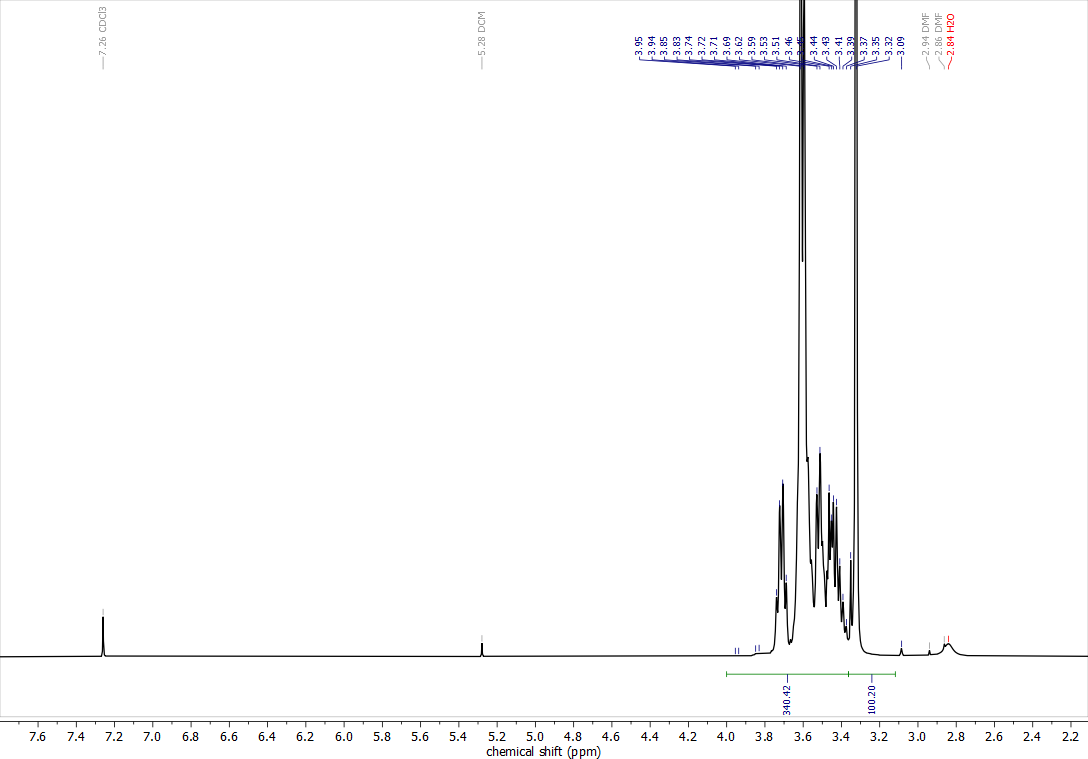


***b***

***a***

***b***

***b***

***b***

***b***

***b***

***b***

***c***

***b***

***b***

***a***

***b***

**n/m**

***b, c‘***

***a, c***

*Figure S 16: ^1^H-NMR spectrum (CDCl_3_, 400 MHz) of* $\text{rPEG}_{\text{76}}^{\text{0.52}}$*-azide*

*(c = terminal EO unit, c’ = terminal GME unit).*


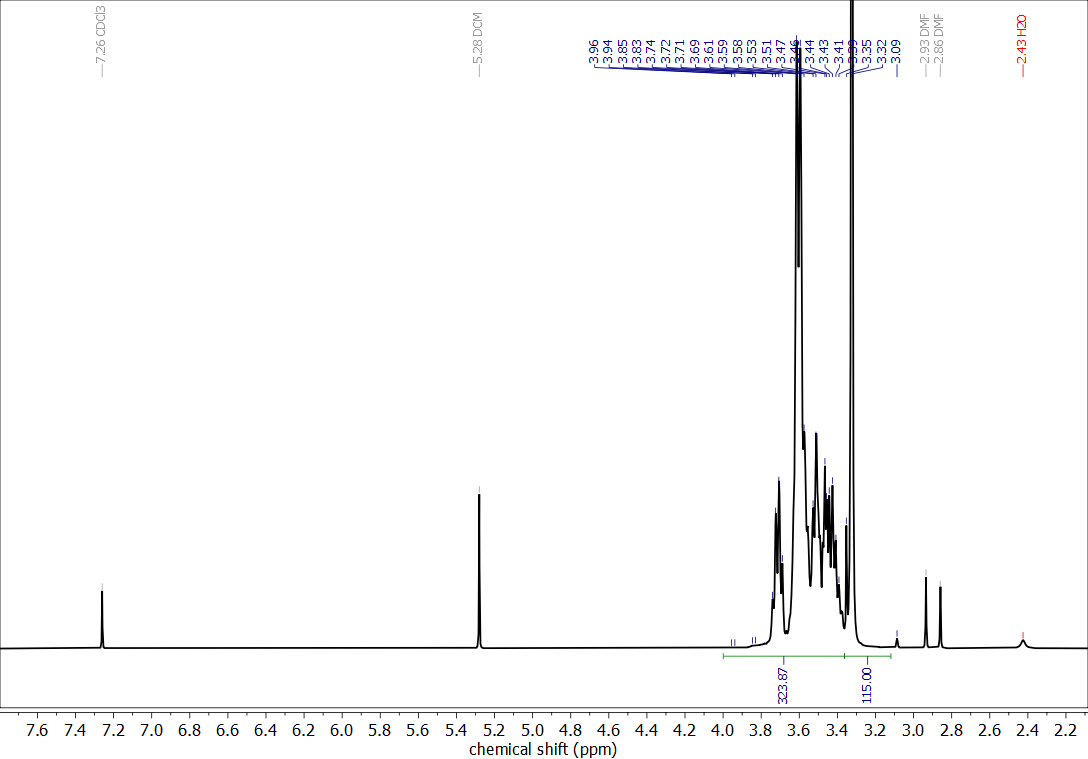


***b, c‘***

***a, c***

***b***

***a***

***b***

***b***

***b***

***b***

***b***

***b***

***c***

***b***

***b***

***a***

***b***

**n/m**

Figure S 17: ^1^H-NMR spectrum (D_2_O, 400 MHz) of $\text{mPEG}_{\text{114}}$-amine.


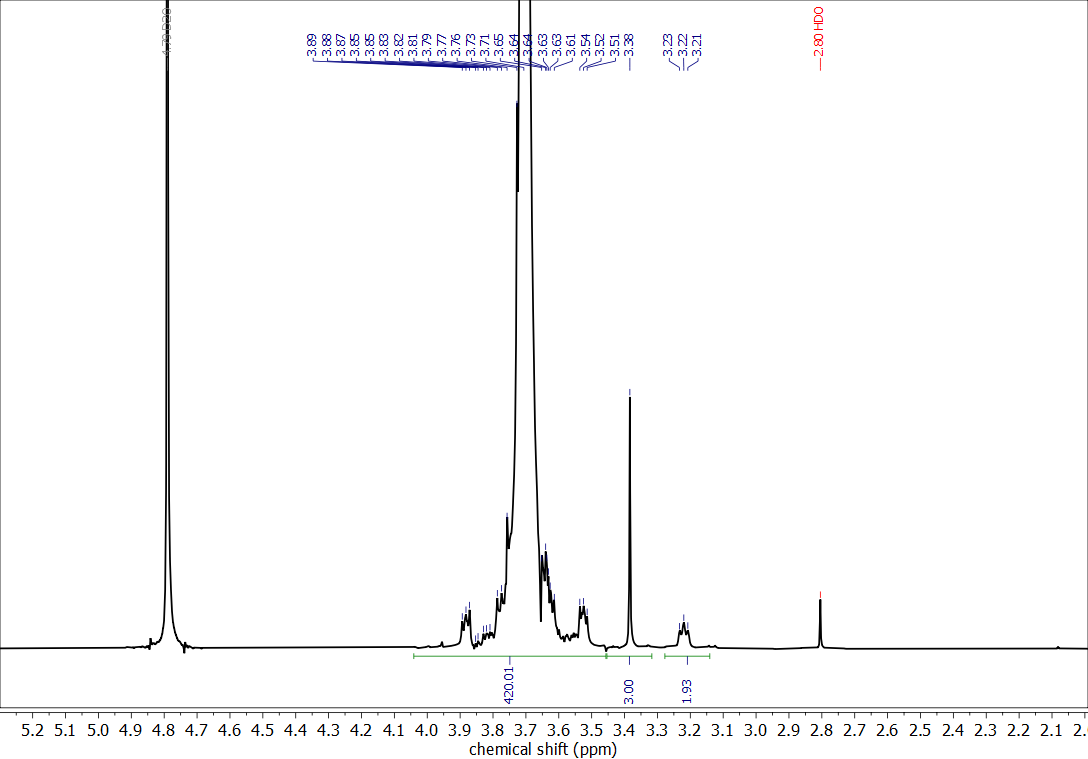


***b***

***a***

***c***

***a***

***b***

***b***

***b***

***b***

***b***

***b***

***b***

***c***

**n**

*Figure S 18: ^1^H-NMR spectrum (D_2_O, 400 MHz) of* $\text{rPEG}_{\text{98}}^{\text{0.17}}$*-amine*

*(c = terminal EO unit, c’ = terminal GME unit).*


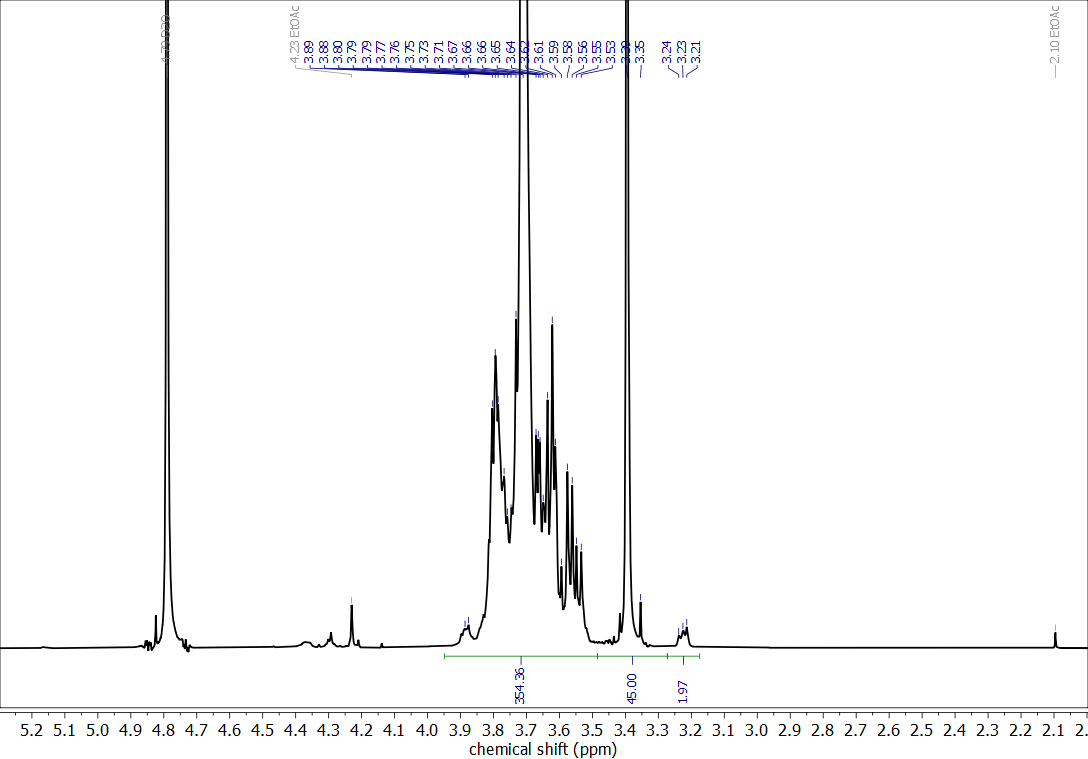


***a***

***b***

***b***

***b***

***b***

***b***

***b***

***b***

***c***

**n/m**

***b***

***b***

***b***

***a***

***b, c‘***

***a***

***c***

*Figure S 19: ^1^H-NMR spectrum (D_2_O, 400 MHz) of* $\text{rPEG}_{\text{89}}^{\text{0.27}}$*-amine*

*(c = terminal EO unit, c’ = terminal GME unit).*


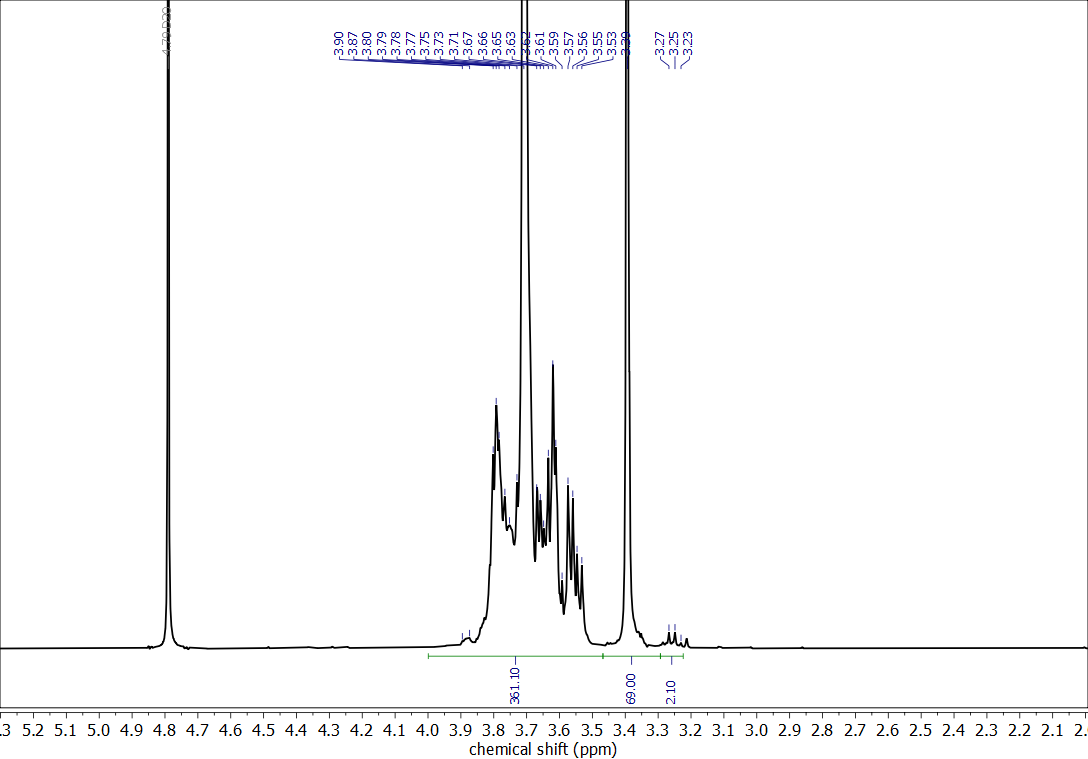


***a***

***b***

***b***

***b***

***b***

***b***

***b***

***b***

***c***

**n/m**

***b***

***b***

***b***

***a***

***b, c‘***

***a***

***c***

*Figure S 20: ^1^H-NMR spectrum (D_2_O, 400 MHz) of* $\text{rPEG}_{\text{91}}^{\text{0.42}}$*-amine*

*(c = terminal EO unit, c’ = terminal GME unit).*


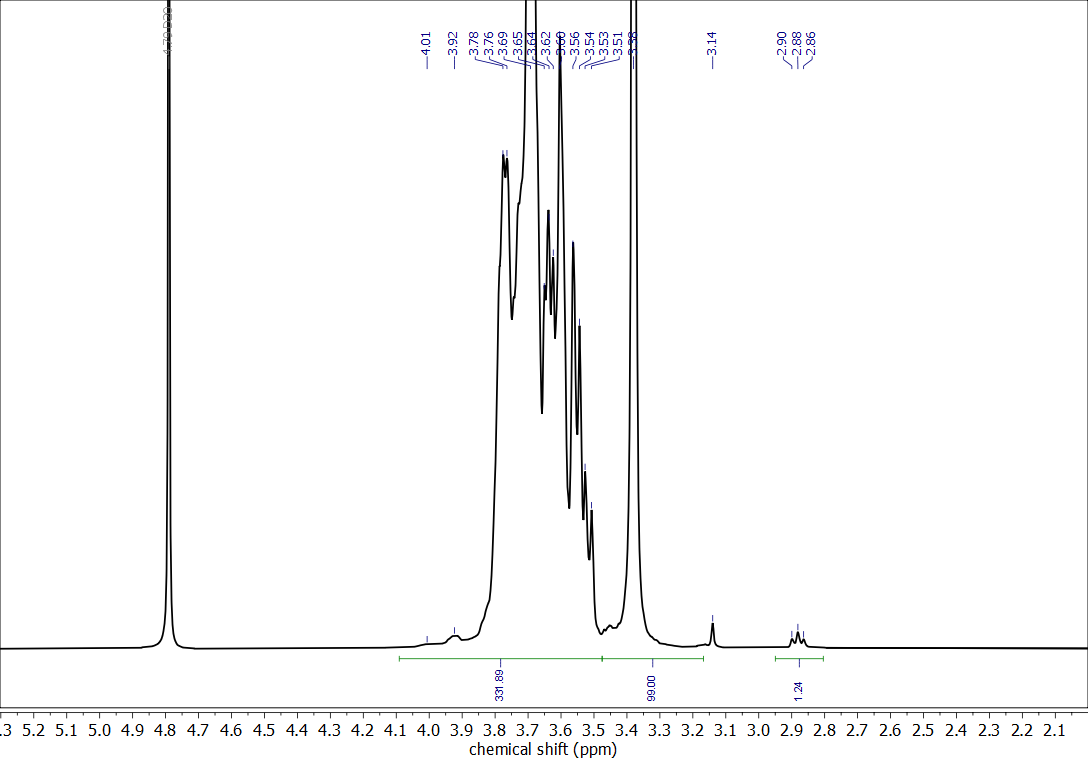


***a***

***b***

***b***

***b***

***b***

***b***

***b***

***b***

***c***

**n/m**

***b***

***b***

***b***

***a***

***b, c‘***

***a***

***c***

*Figure S 21: ^1^H-NMR spectrum (D_2_O, 400 MHz) of* $\text{rPEG}_{\text{76}}^{\text{0.52}}$*-amine*

*(c = terminal EO unit, c’ = terminal GME unit).*


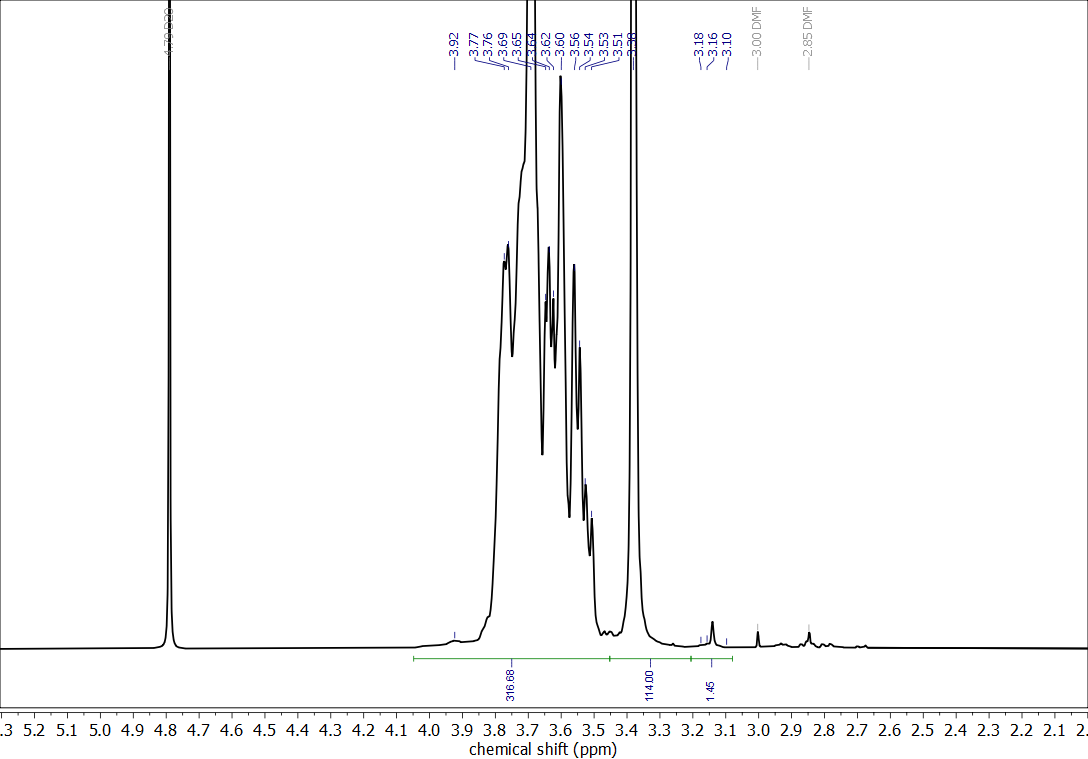


***a***

***b***

***b***

***b***

***b***

***b***

***b***

***b***

***c***

**n/m**

***b***

***b***

***b***

***a***

***b, c‘***

***a***

***c***

Figure S 22: ^1^H-NMR spectrum (D_2_O, 400 MHz) of $\text{mPEG}_{\text{114}}$-FITC.


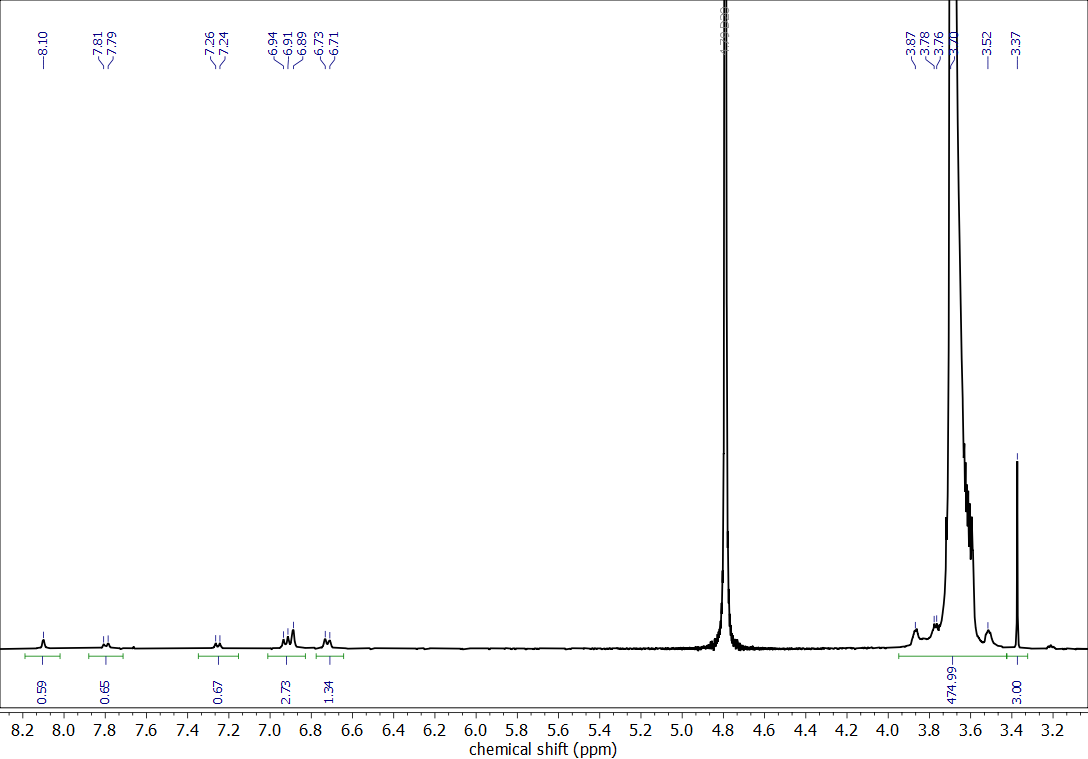


***a***

***b***

***b***

***b***

***b***

***b***

***c***

**n**

***b***

***b***

***d***

***e***

***f***

***g***

***h***

***h***

***h***

***h***

***g***

***b, c***

***a***

***d***

***e***

***f***

***h***

***g***

*Figure S 23: ^1^H-NMR spectrum (D_2_O, 400 MHz) of* $\text{rPEG}_{\text{98}}^{\text{0.17}}$*-FITC*

*(c = terminal EO unit, c’ = terminal GME unit).*


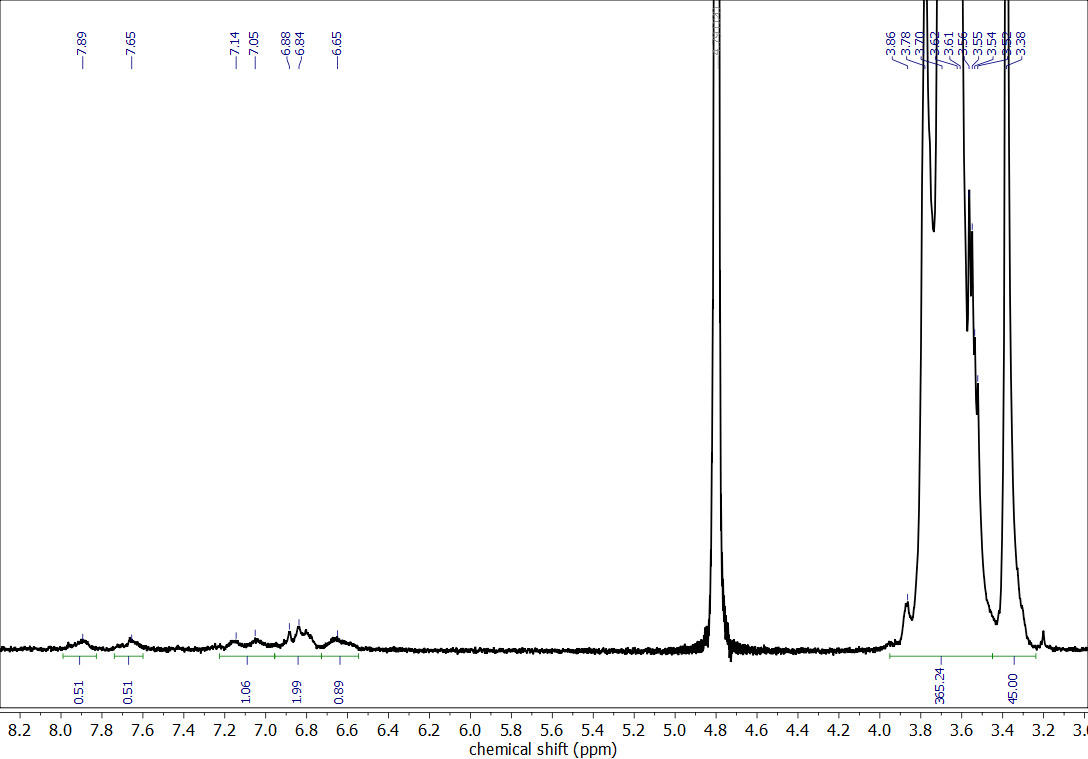


***b, c, c‘***

***a***

***d***

***e***

***f***

***h***

***g***

***a***

***b***

***b***

***b***

***b***

***b***

***b***

***b***

***c***

**n/m**

***b***

***b***

***b***

***a***

***d***

***e***

***f***

***g***

***h***

***h***

***h***

***h***

***g***

*Figure S 24: ^1^H-NMR spectrum (D_2_O, 400 MHz) of* $\text{rPEG}_{\text{89}}^{\text{0.27}}$*-FITC*

*(c = terminal EO unit, c’ = terminal GME unit).*


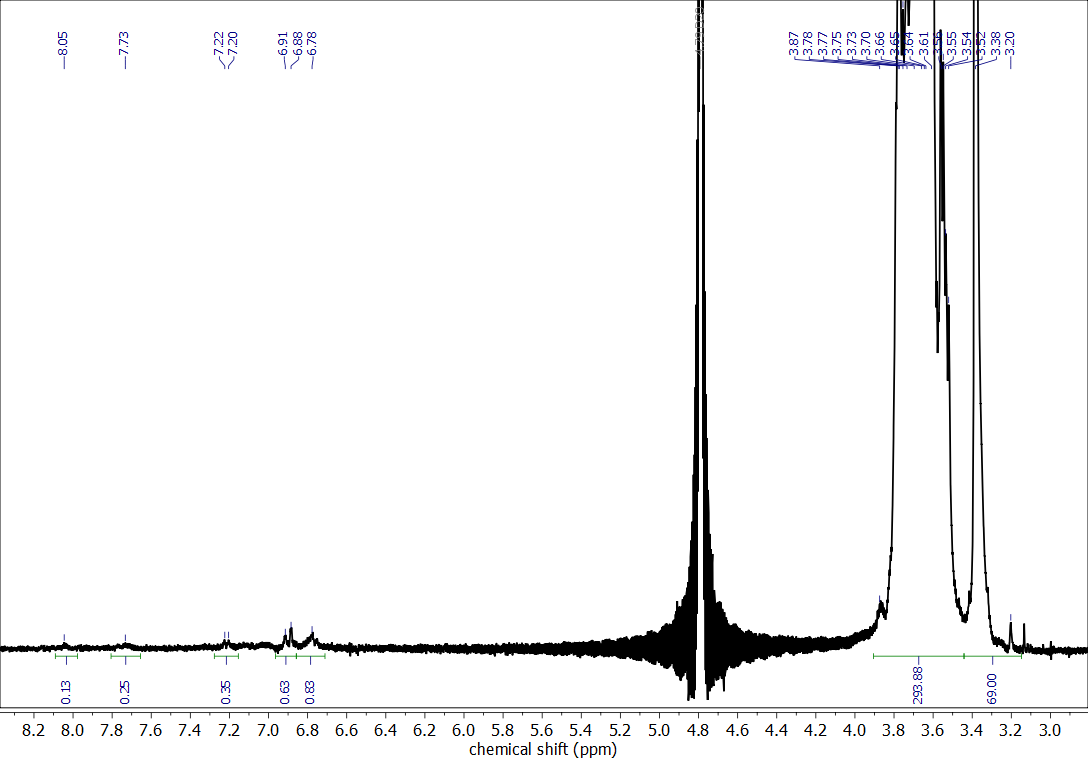


***a***

***b***

***b***

***b***

***b***

***b***

***b***

***b***

***c***

**n/m**

***b***

***b***

***b***

***a***

***d***

***e***

***f***

***g***

***h***

***h***

***h***

***h***

***g***

***b, c, c‘***

***a***

***d***

***e***

***f***

***g, h***

*Figure S 25: ^1^H-NMR spectrum (D_2_O, 400 MHz) of* $\text{rPEG}_{\text{91}}^{\text{0.42}}$*-FITC*

*(c = terminal EO unit, c’ = terminal GME unit).*


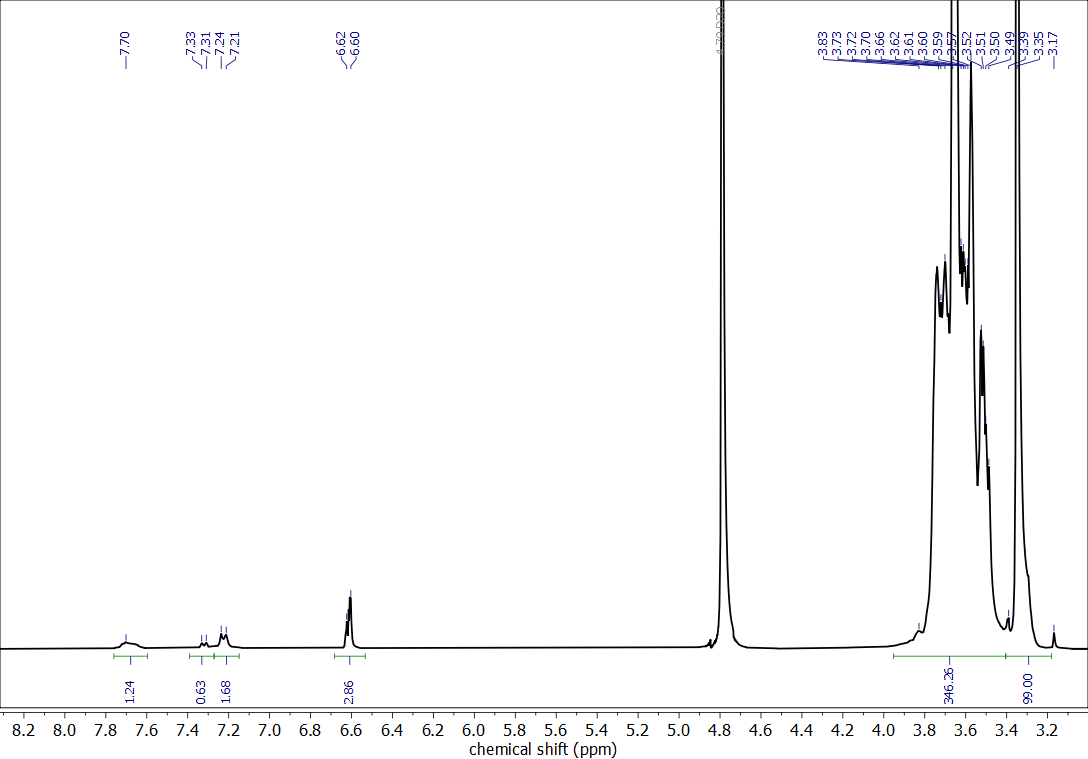


***a***

***b***

***b***

***b***

***b***

***b***

***b***

***b***

***c***

**n/m**

***b***

***b***

***b***

***a***

***d***

***e***

***f***

***g***

***h***

***h***

***h***

***h***

***g***

***b, c, c‘***

***a***

***d, e***

***g***

***h***

***f***

*Figure S 26: ^1^H-NMR spectrum (D_2_O, 400 MHz) of* $\text{rPEG}_{\text{76}}^{\text{0.52}}$*-FITC*

*(c = terminal EO unit, c’ = terminal GME unit).*


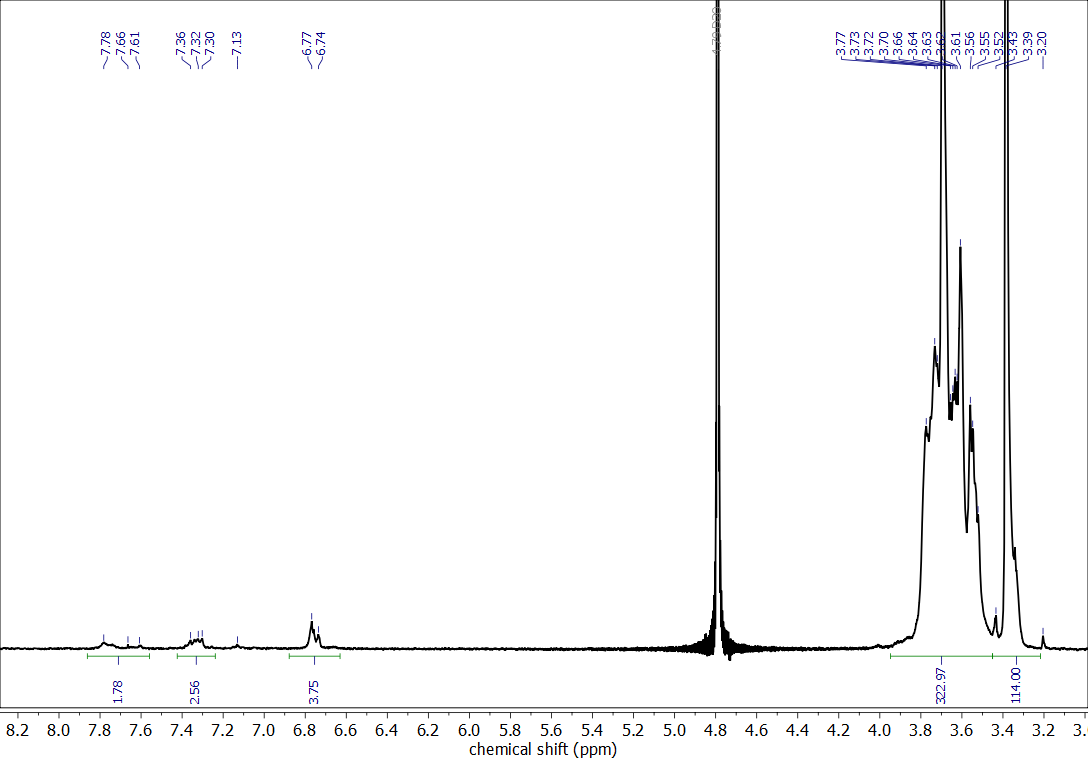


***a***

***b***

***b***

***b***

***b***

***b***

***b***

***b***

***c***

**n/m**

***b***

***b***

***b***

***a***

***d***

***e***

***f***

***g***

***h***

***h***

***h***

***h***

***g***

***b, c, c‘***

***a***

***d, e***

***h***

***f, g***

**3. 2D DOSY-NMR spectra**


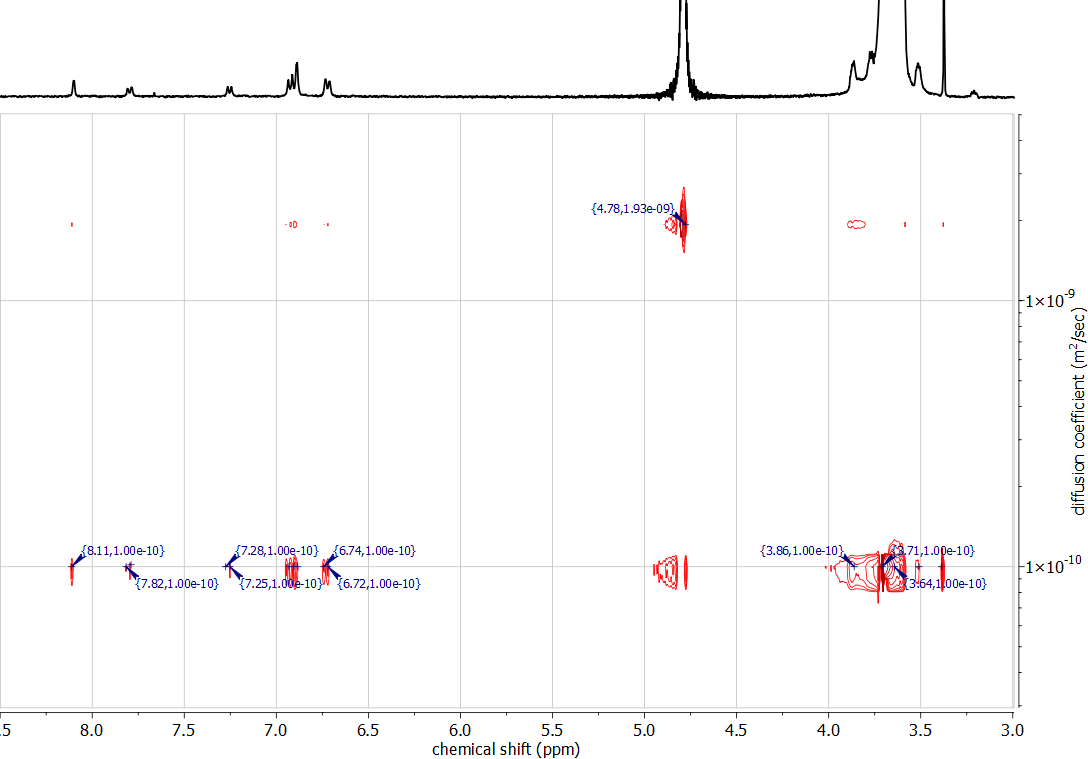


Figure S 27: 2D DOSY-NMR spectrum (D_2_O, 400 MHz) of $\text{mPEG}_{\text{114}}$-FITC.


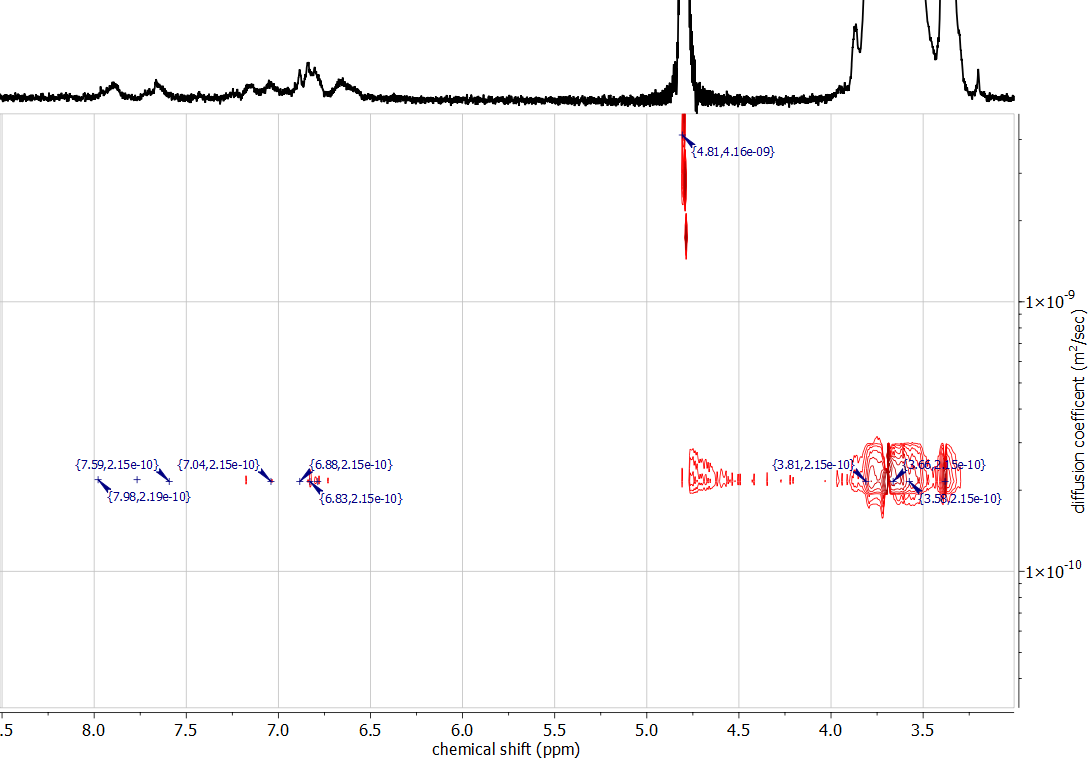


Figure S 28: 2D DOSY-NMR spectrum (D_2_O, 400 MHz) of $\text{rPEG}_{\text{98}}^{\text{0.17}}$-FITC.


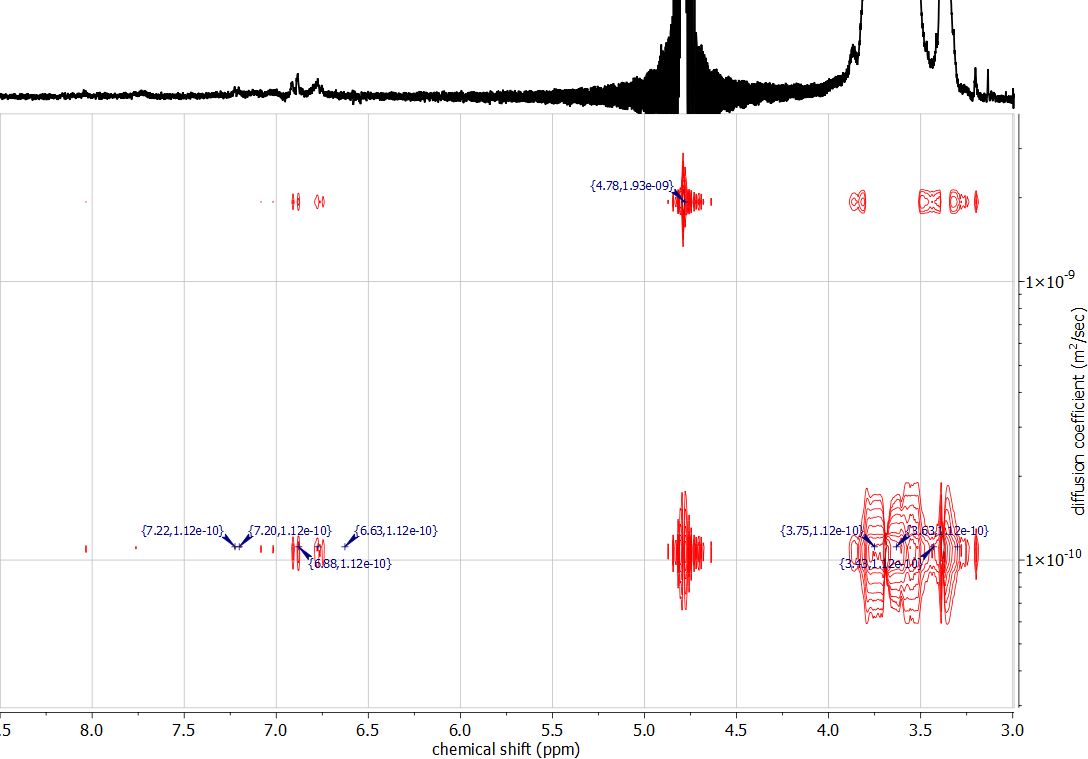


Figure S 29: 2D DOSY-NMR spectrum (D_2_O, 400 MHz) of $\text{rPEG}_{\text{89}}^{\text{0.27}}$-FITC.


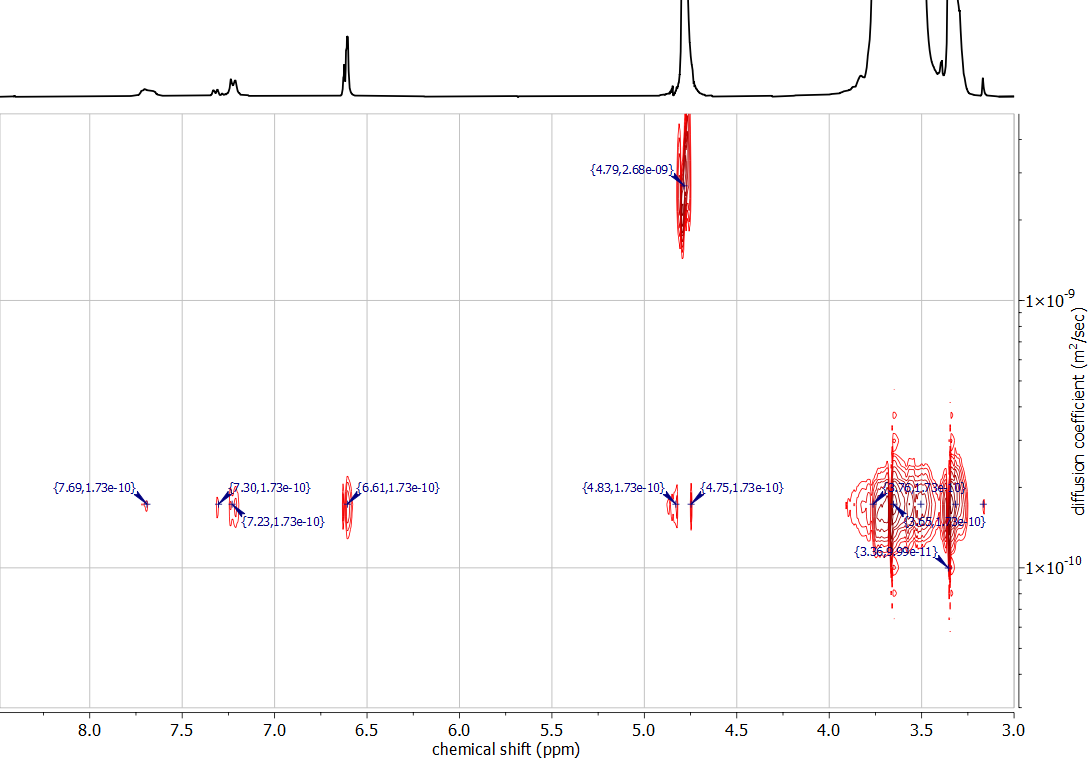


Figure S 30: 2D DOSY-NMR spectrum (D_2_O, 400 MHz) of $\text{rPEG}_{\text{91}}^{\text{0.42}}$-FITC.


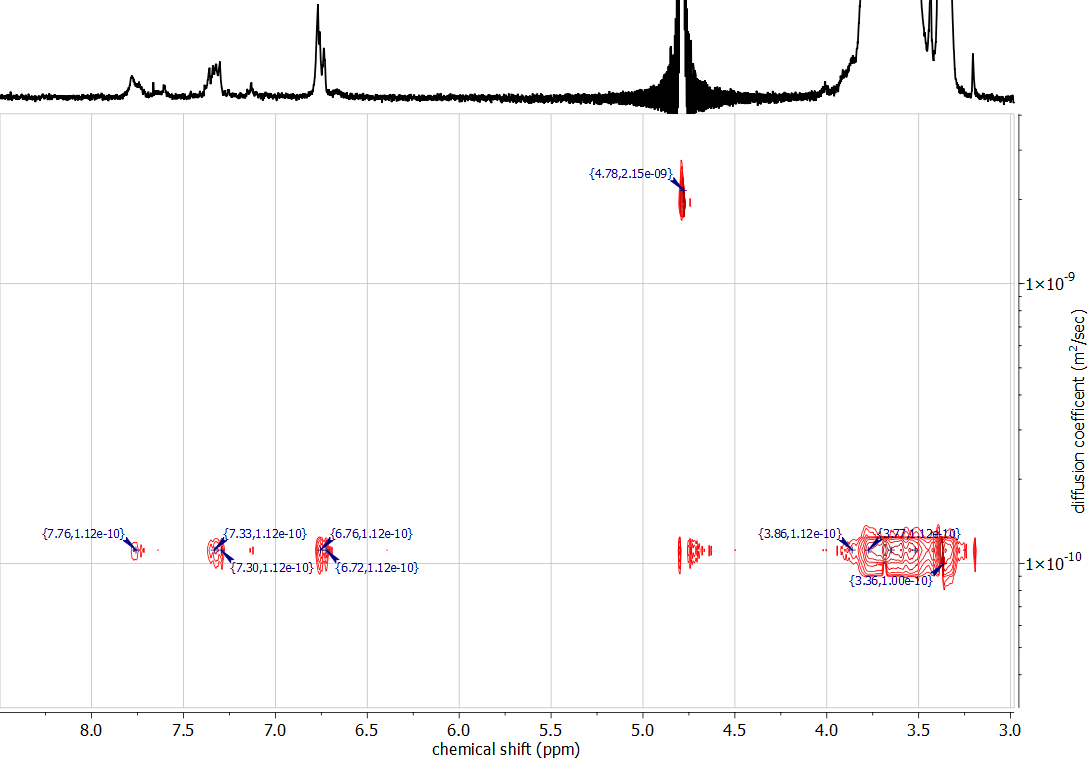


Figure S 31: 2D DOSY-NMR spectrum (D_2_O, 400 MHz) of $\text{rPEG}_{\text{76}}^{\text{0.52}}$-FITC.

**4. Matrix-assisted laser desorption ionization using time-of-flight (MALDI-TOF) mass spectrometry**

Figure S 32: MALDI-TOF mass spectrum of $\text{mPEG}_{\text{114}}$ .


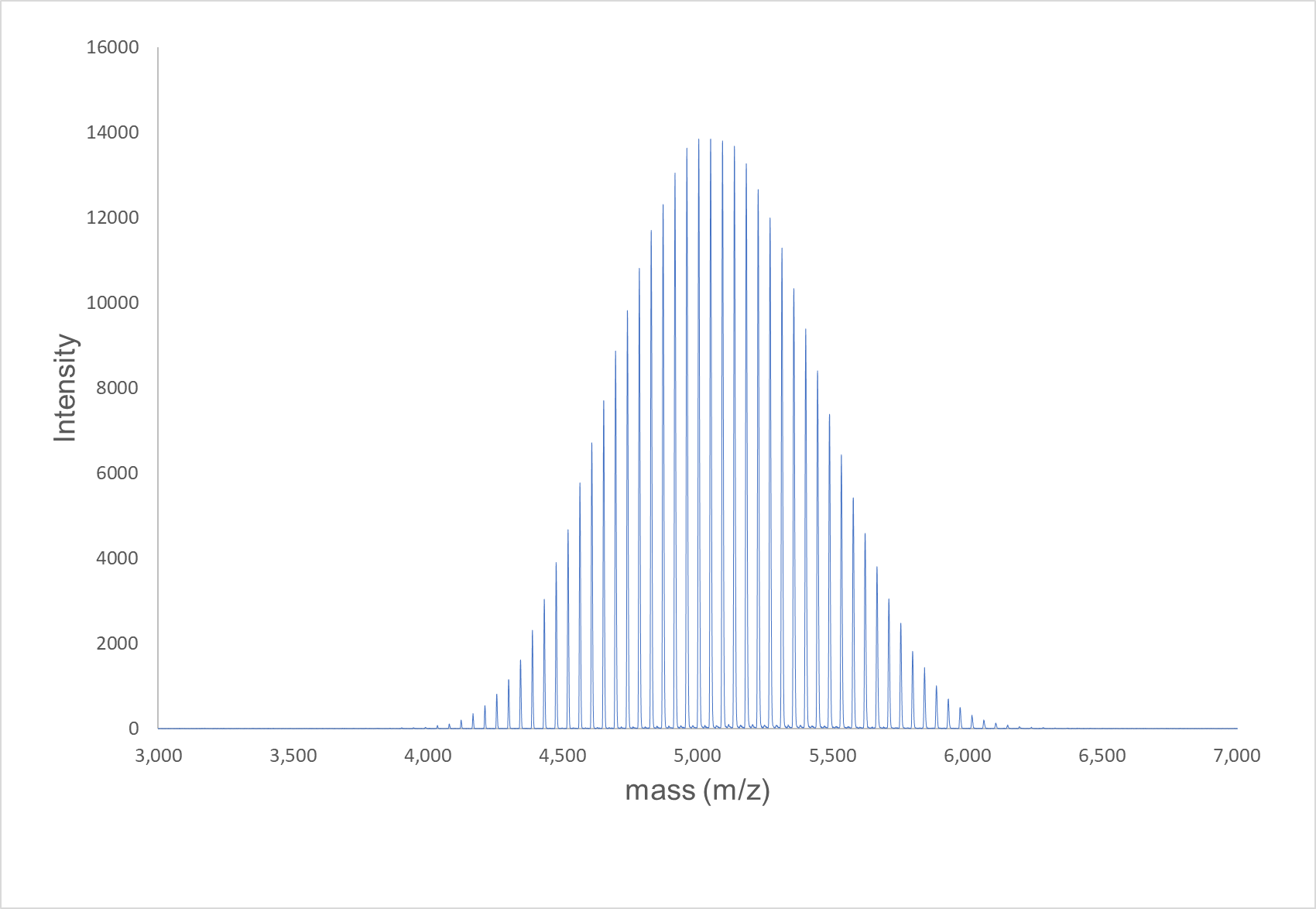

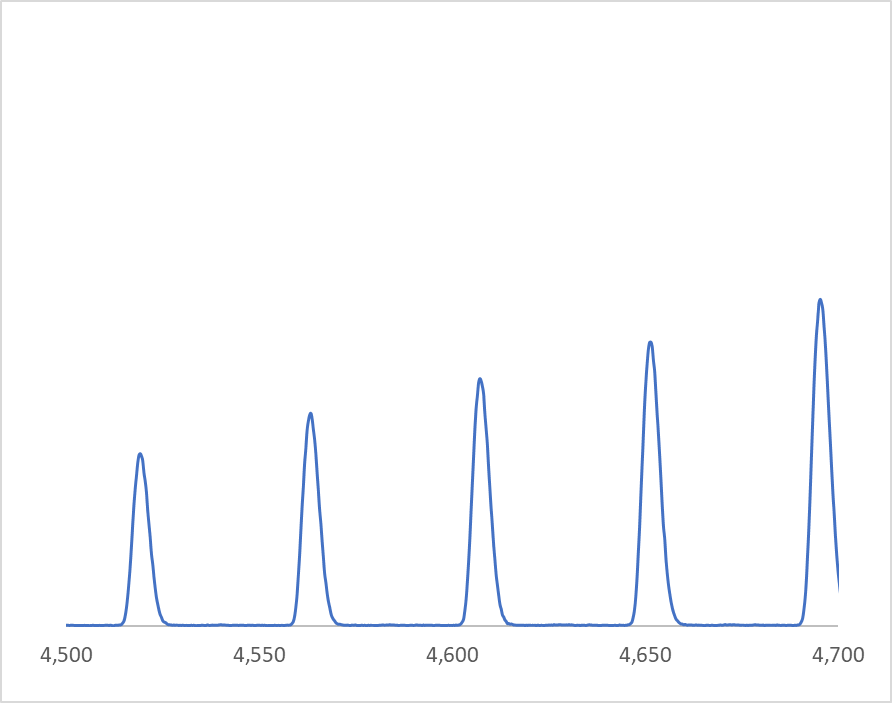

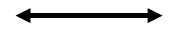


4563.32 [M + K]

4607.42 [M + K]

44.1 Da

Figure S 33: MALDI-TOF mass spectrum of $\text{mPEG}_{\text{114}}$-mesylate.


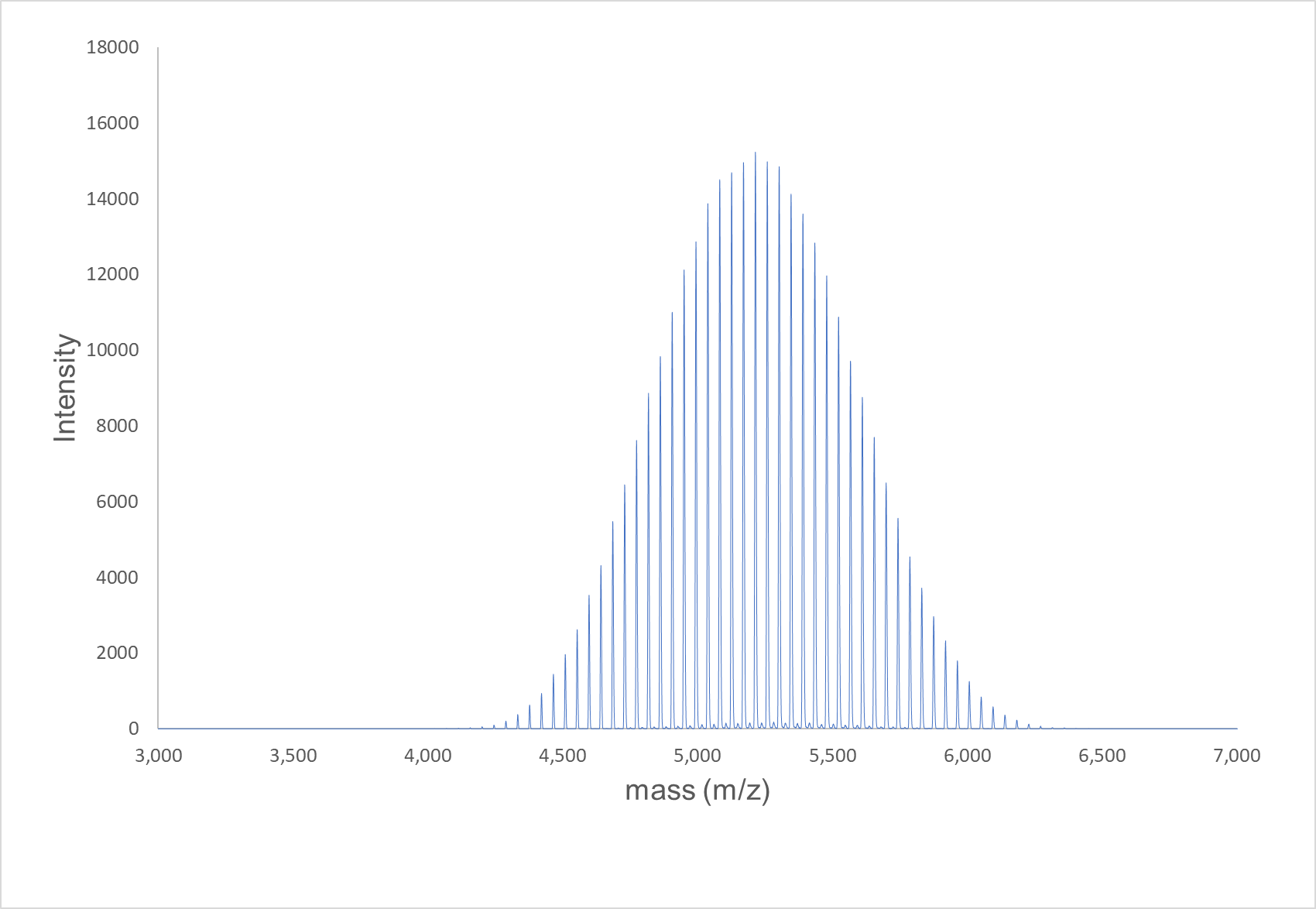

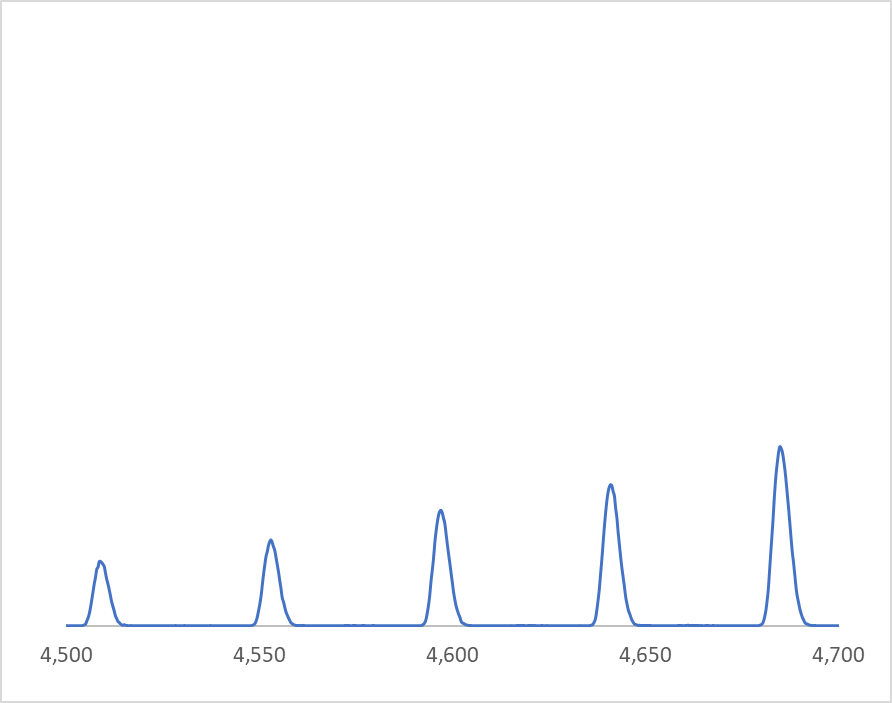

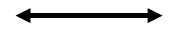


4552.97 [M + K]

4597.02 [M + K]

44.1 Da

Figure S 34: MALDI- TOF mass spectrum of $\text{mPEG}_{\text{114}}$-azide.


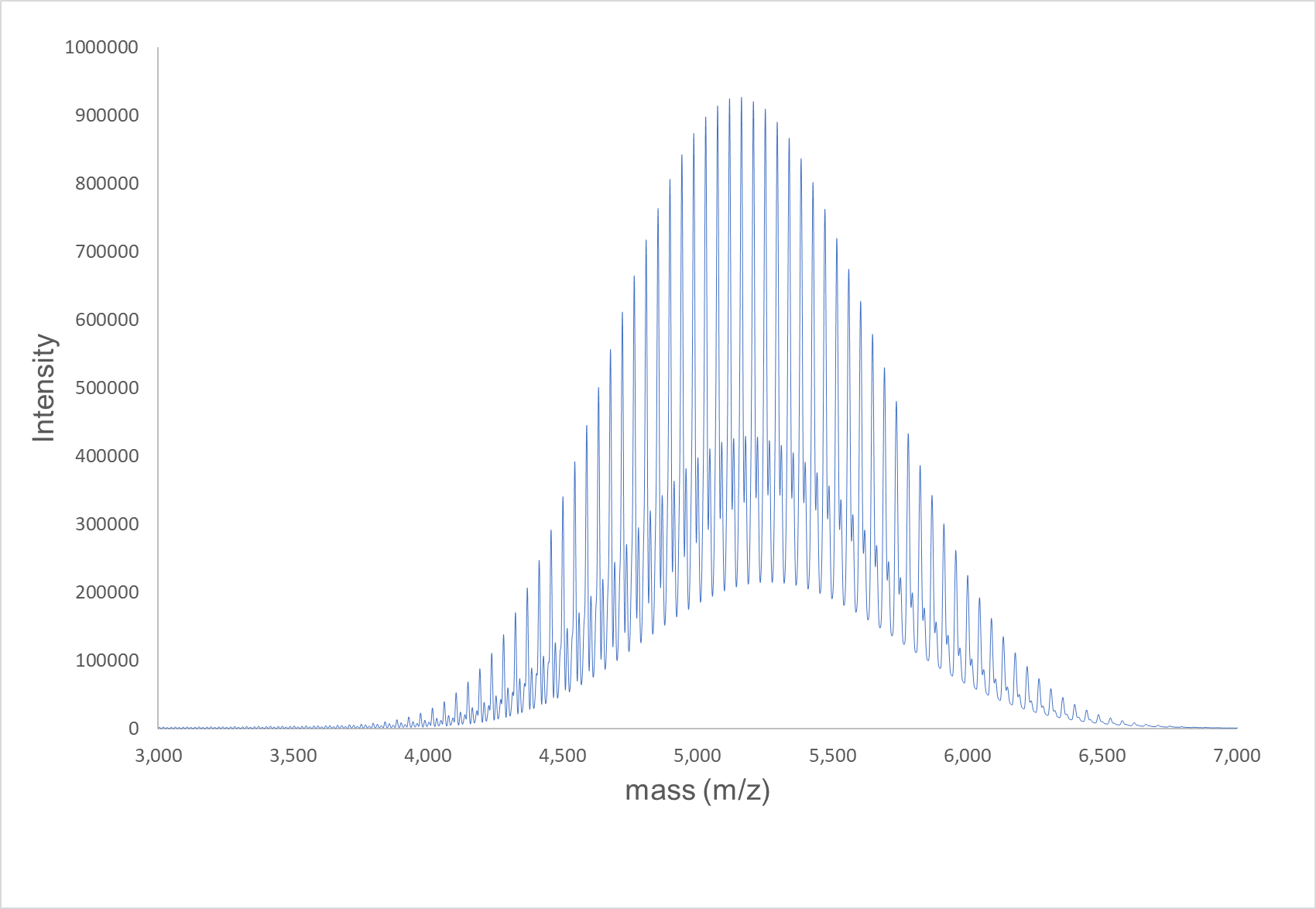

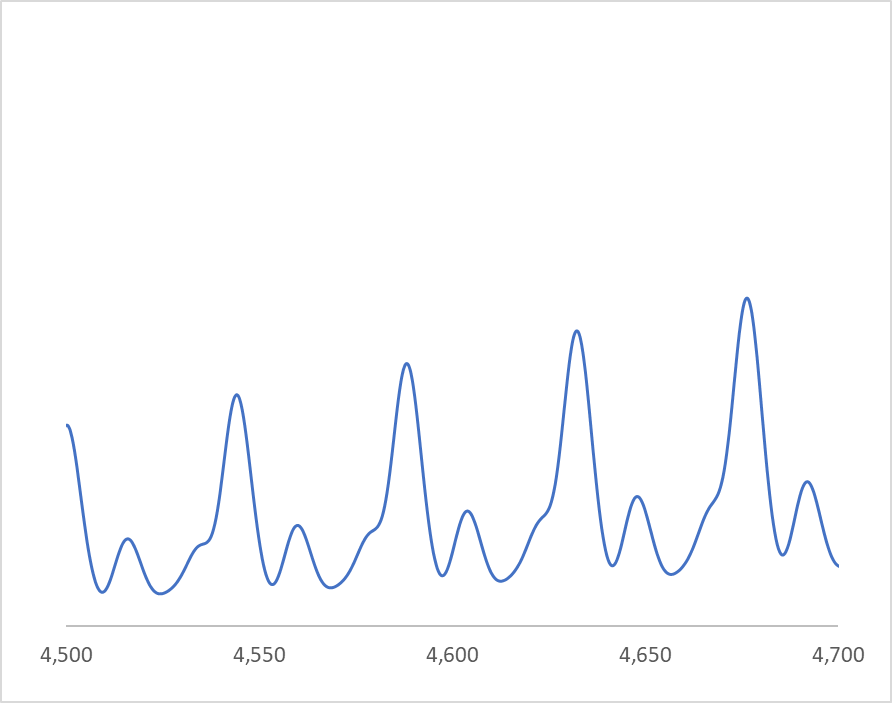

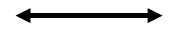


4544.25 [M + K]

4588.26 [M + K]

44.0 Da


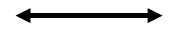


4647.82
[M - N_2_ + Na]

4692.00
[M - N_2_ + Na]

44.2 Da

Figure S 35: MALDI-TOF mass spectrum of $\text{mPEG}_{\text{114}}$-amine.


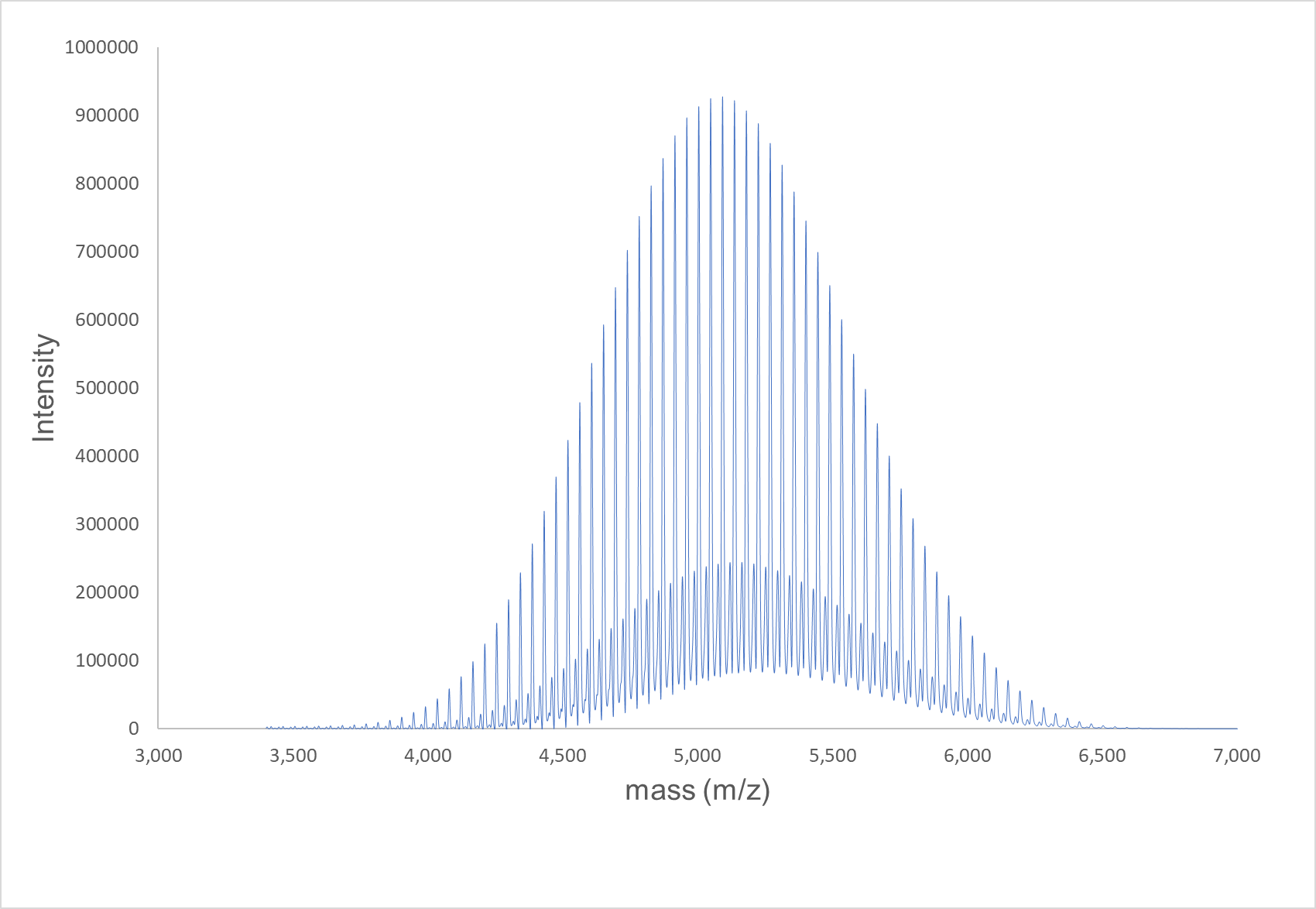

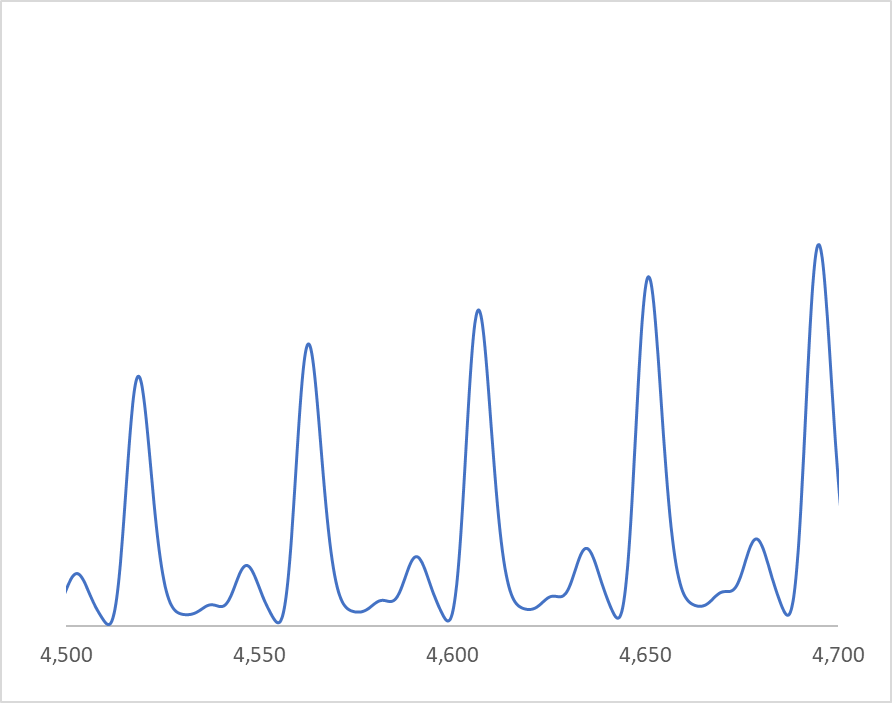

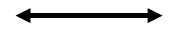


4518.77 [M + K]

4562.69 [M + K]

43.9 Da


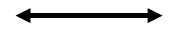


4634.77 [M + Na]

4678.89 [M + Na]

44.1 Da

Figure S 36: MALDI-TOF mass spectrum of $\text{mPEG}_{\text{114}}$-FITC.


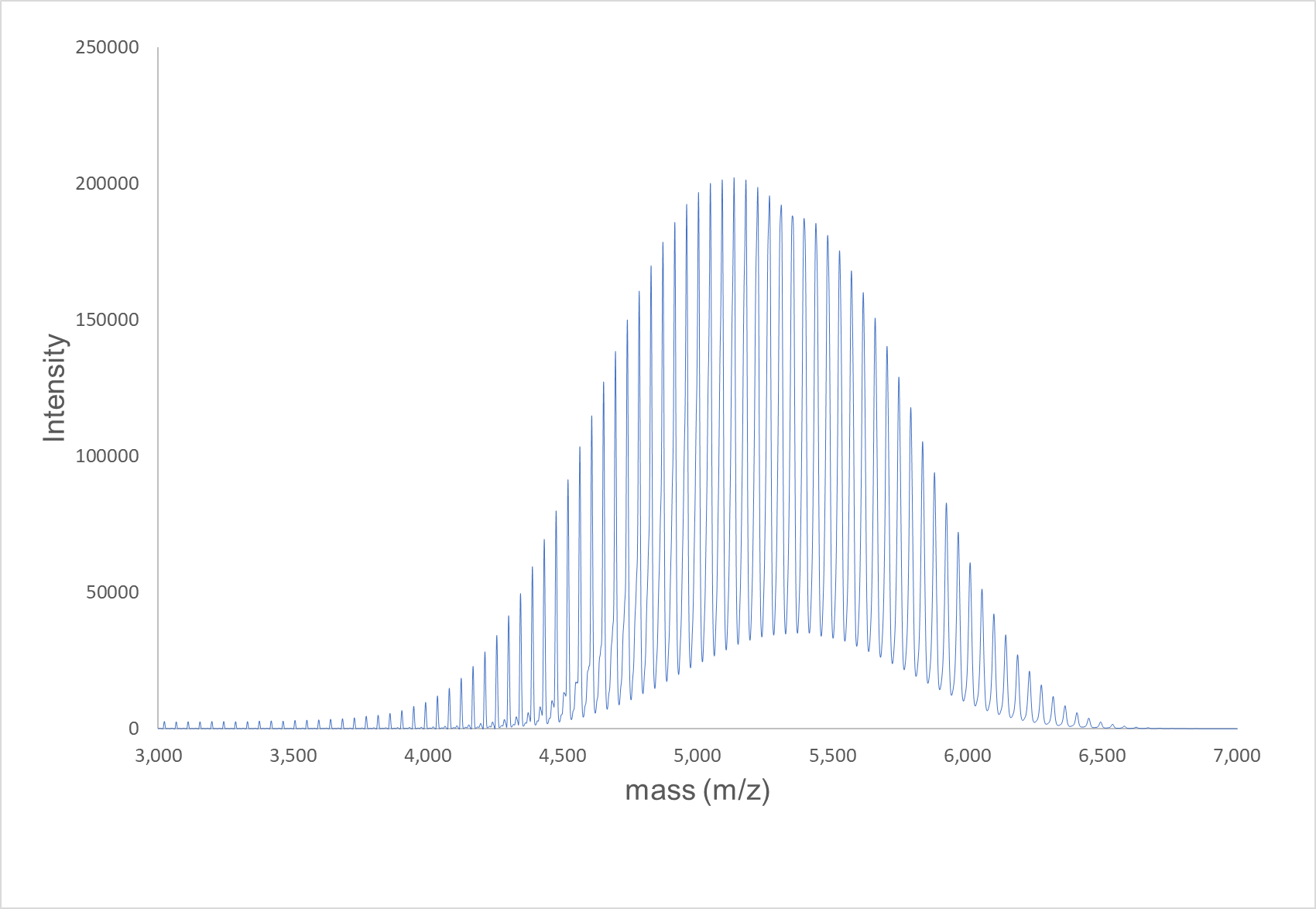

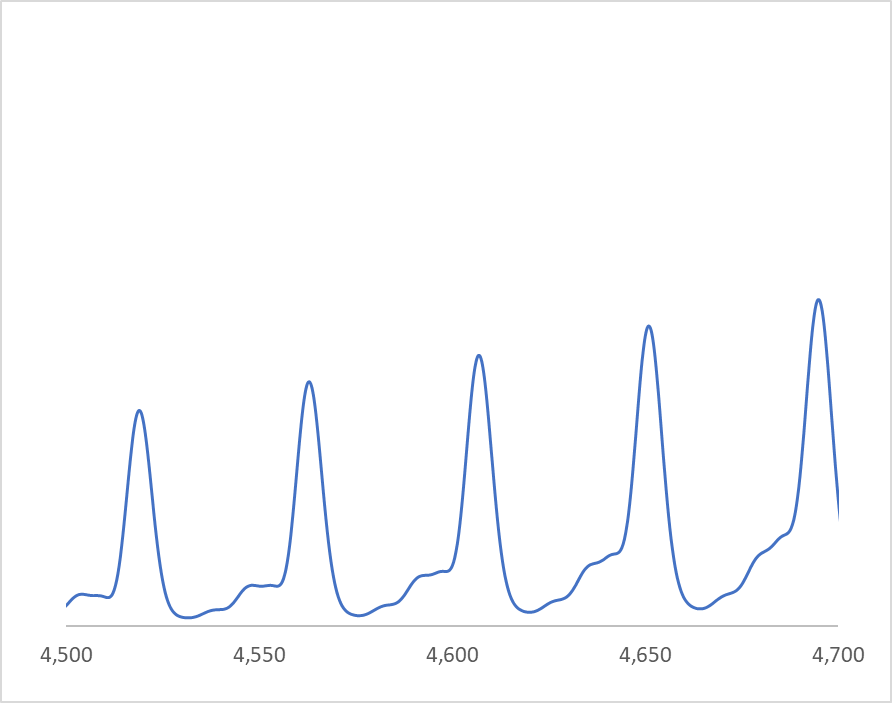

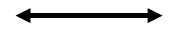


4562.35 [M + H]

4606.45 [M + H]

44.1 Da

Figure S 37: MALDI-TOF mass spectrum of $\text{rPEG}_{\text{98}}^{\text{0.17}}$.


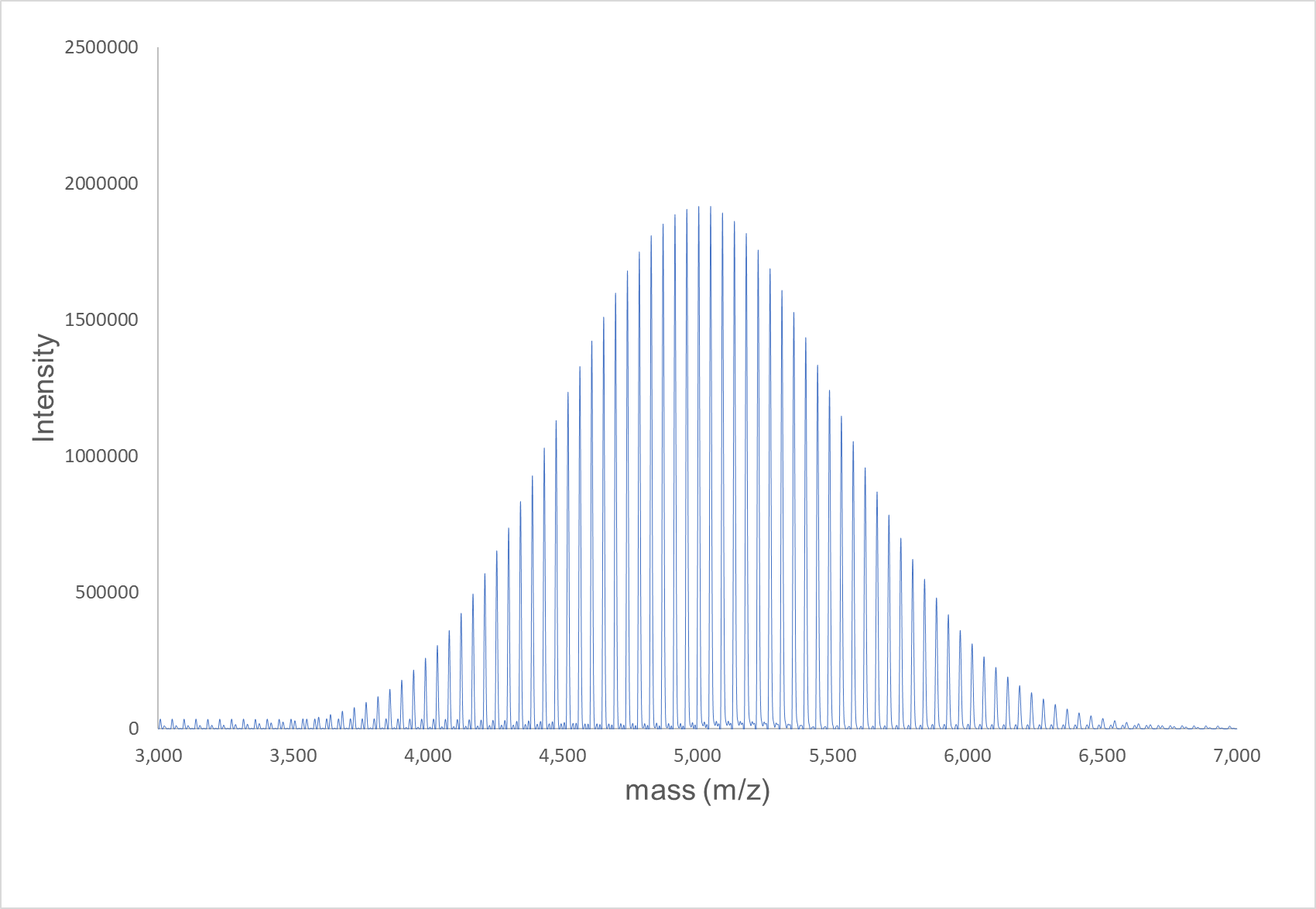

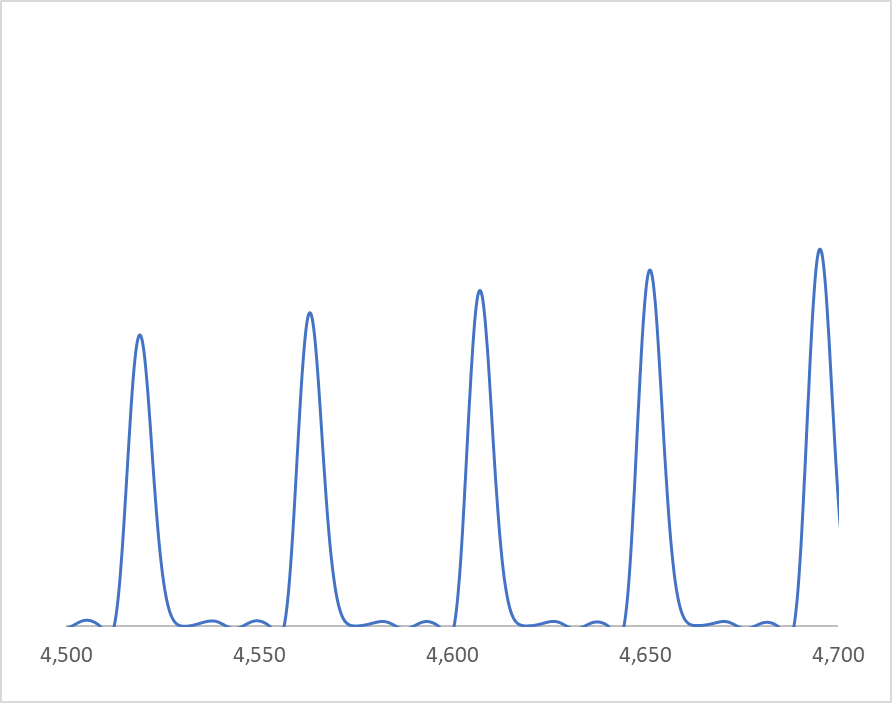

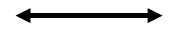


4563.02 [M + K]

4607.26 [M + K]

44.2 Da

Figure S 38: MALDI-TOF mass spectrum of $\text{rPEG}_{\text{98}}^{\text{0.17}}$-mesylate.


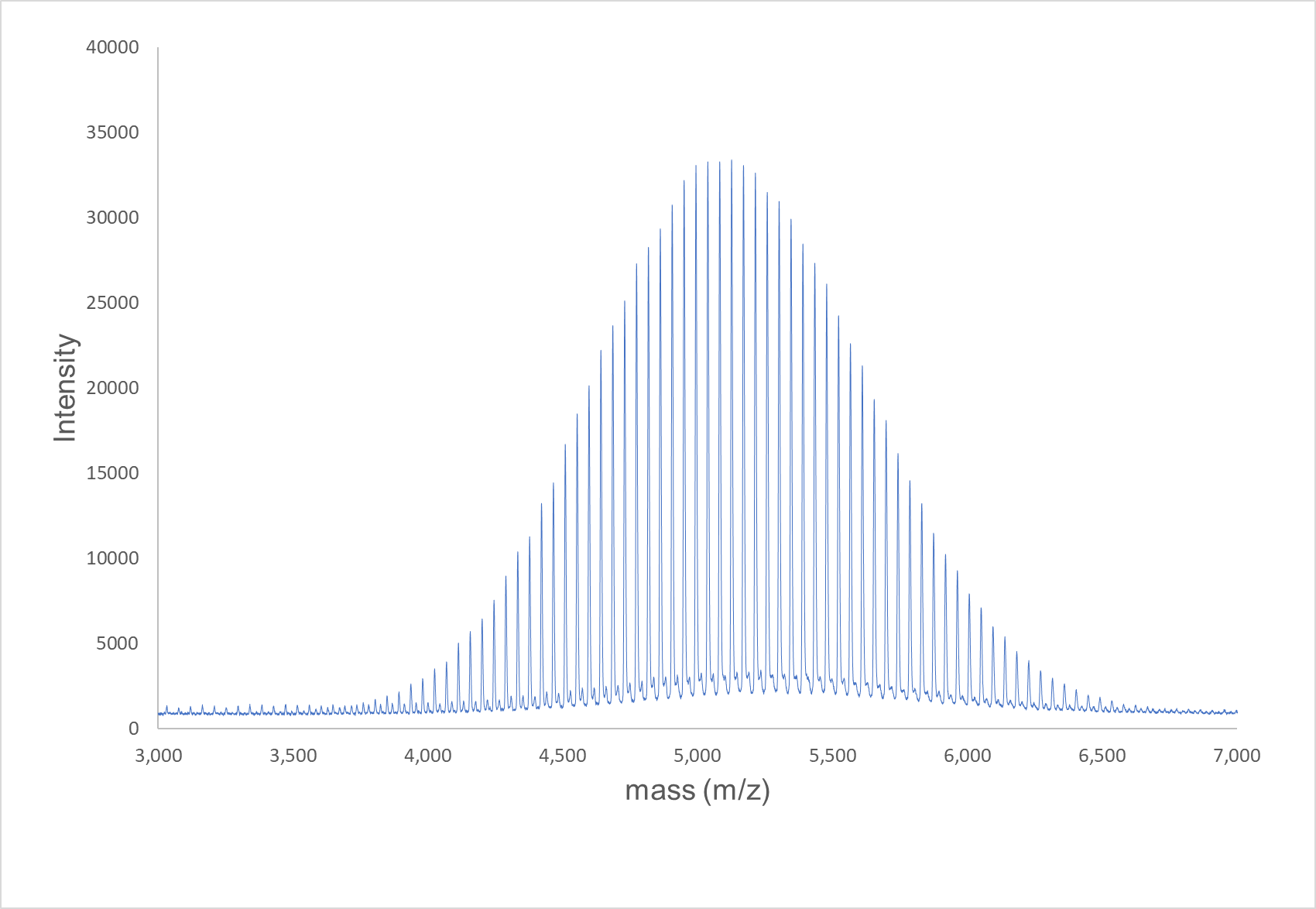

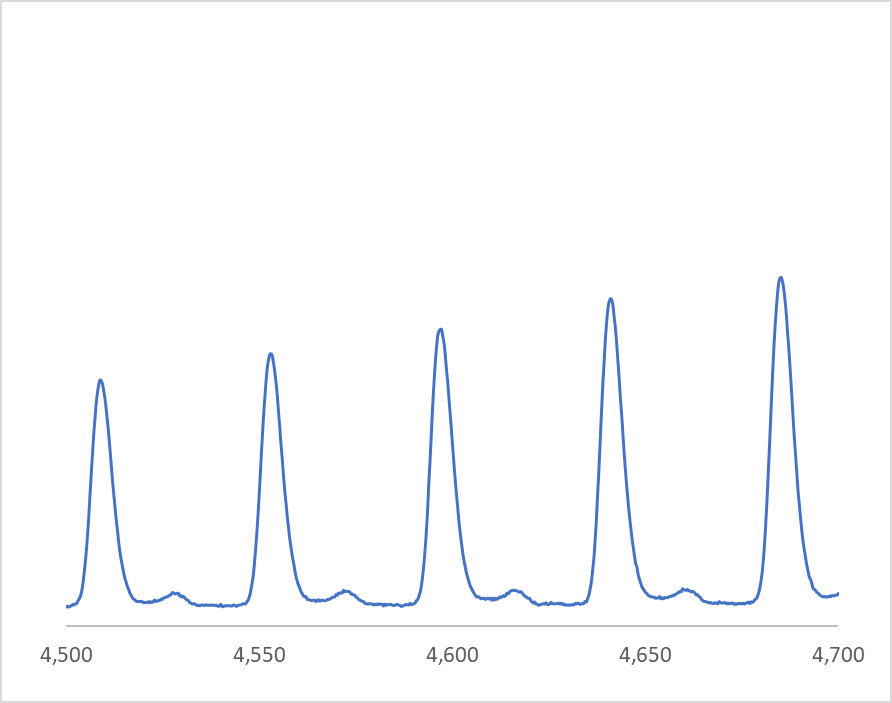

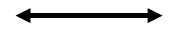


4597.15 [M + K]

4641.23 [M + K]

44.1 Da

*Figure S 39: MALDI-TOF mass spectrum of* $\text{rPEG}_{\text{98}}^{\text{0.17}}$*-azide.*


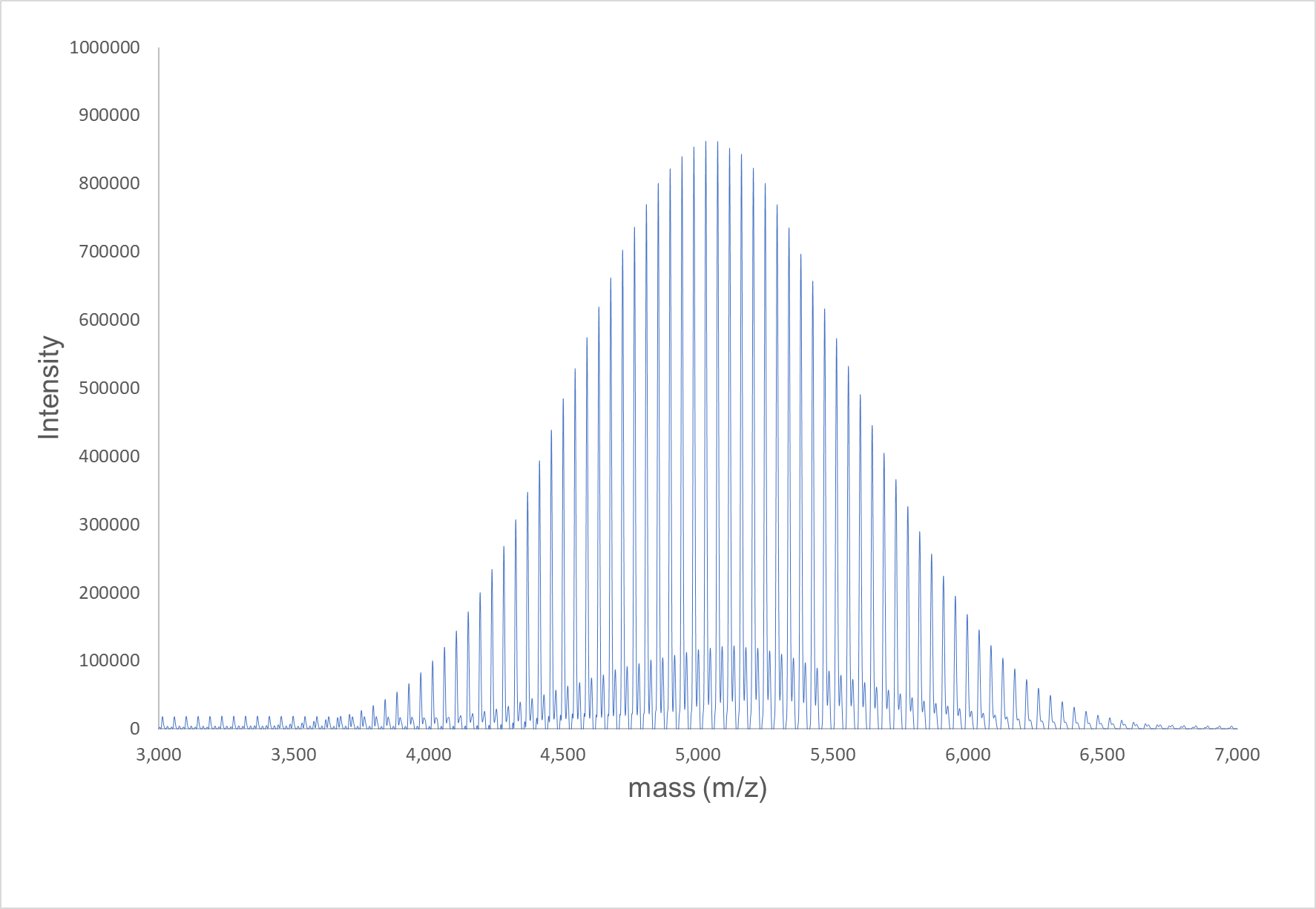

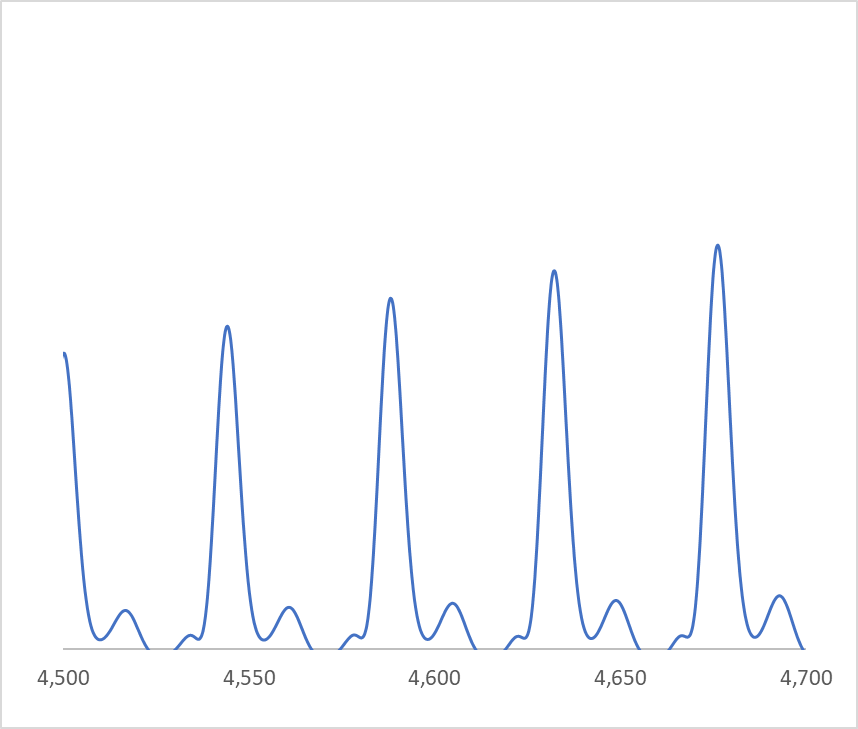


44.2 Da


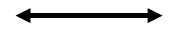


4632.07 [M + K]

4676.31 [M + K]

44.0 Da


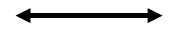


4516.74
[M - N_2_ + K]

4560.75
[M - N_2_ + K]

Figure S 40: MALDI-TOF mass spectrum of $\text{rPEG}_{\text{98}}^{\text{0.17}}$-amine.


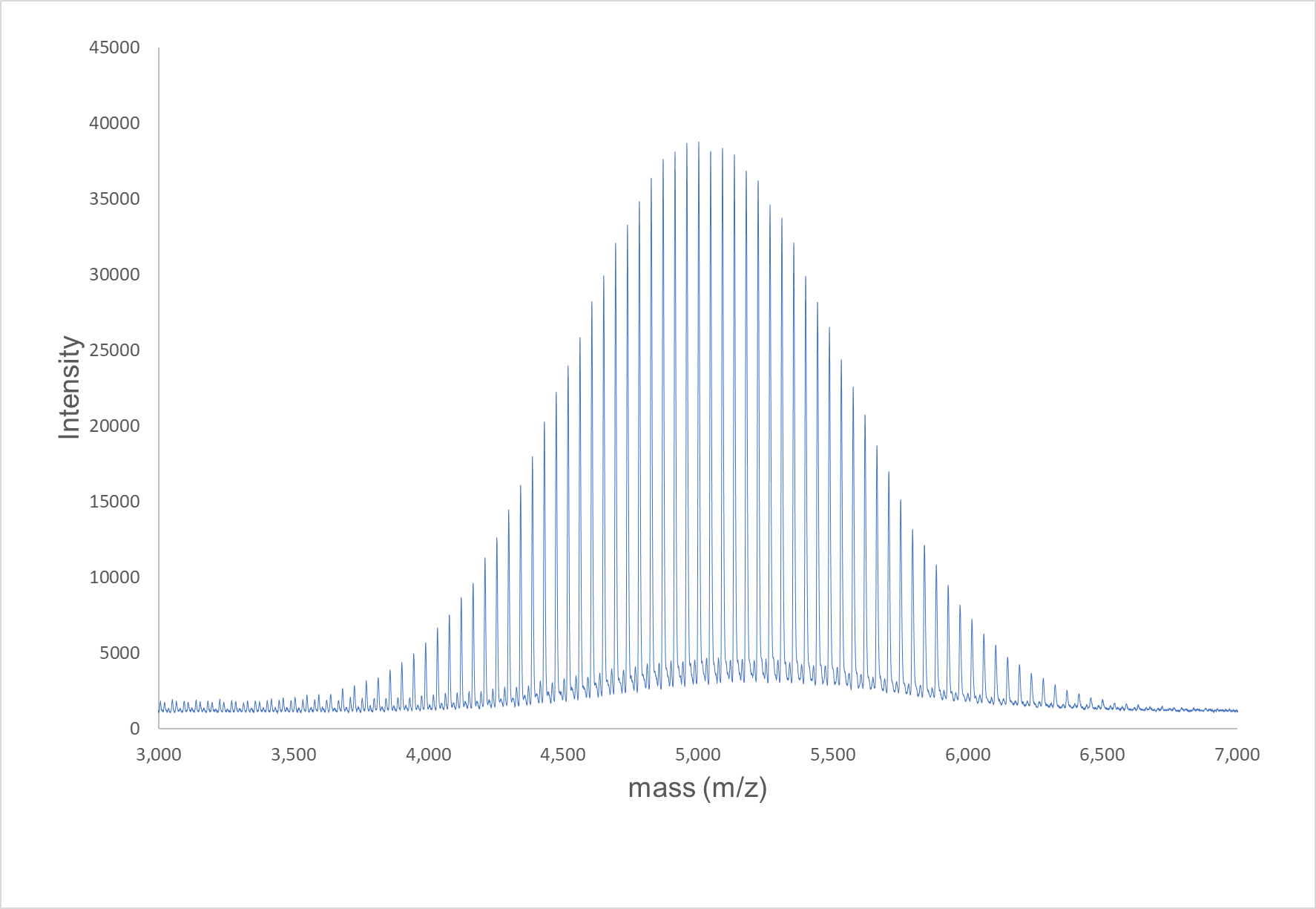

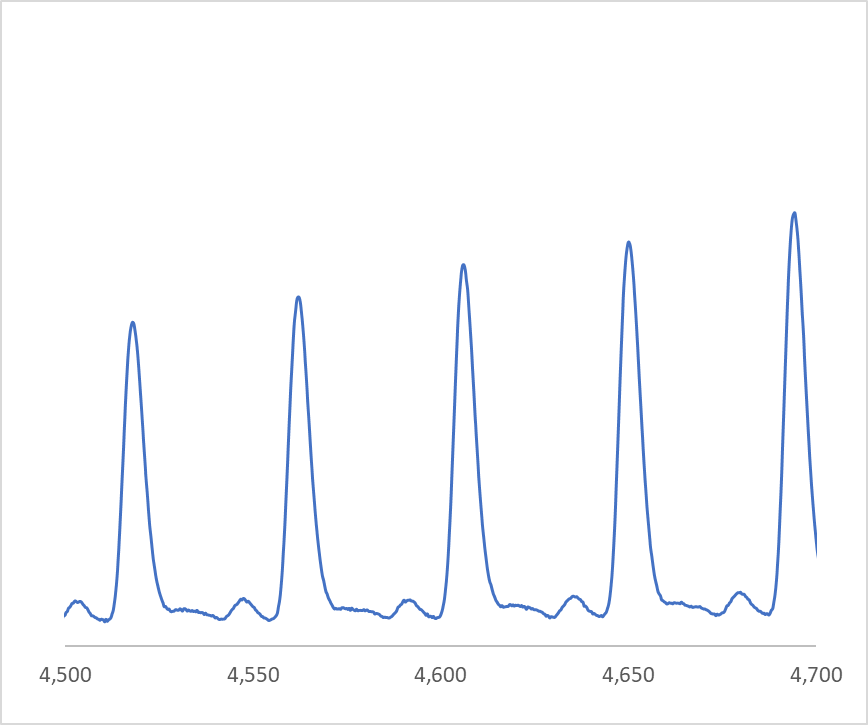


44.2 Da


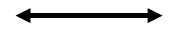


4650.07 [M + K]

4694.07 [M + K]

44.2 Da


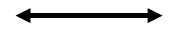


4645.49 [M + Na]

4590.64 [M + Na]

Figure S 41: MALDI-TOF mass spectrum of $\text{rPEG}_{\text{98}}^{\text{0.17}}$-FITC.


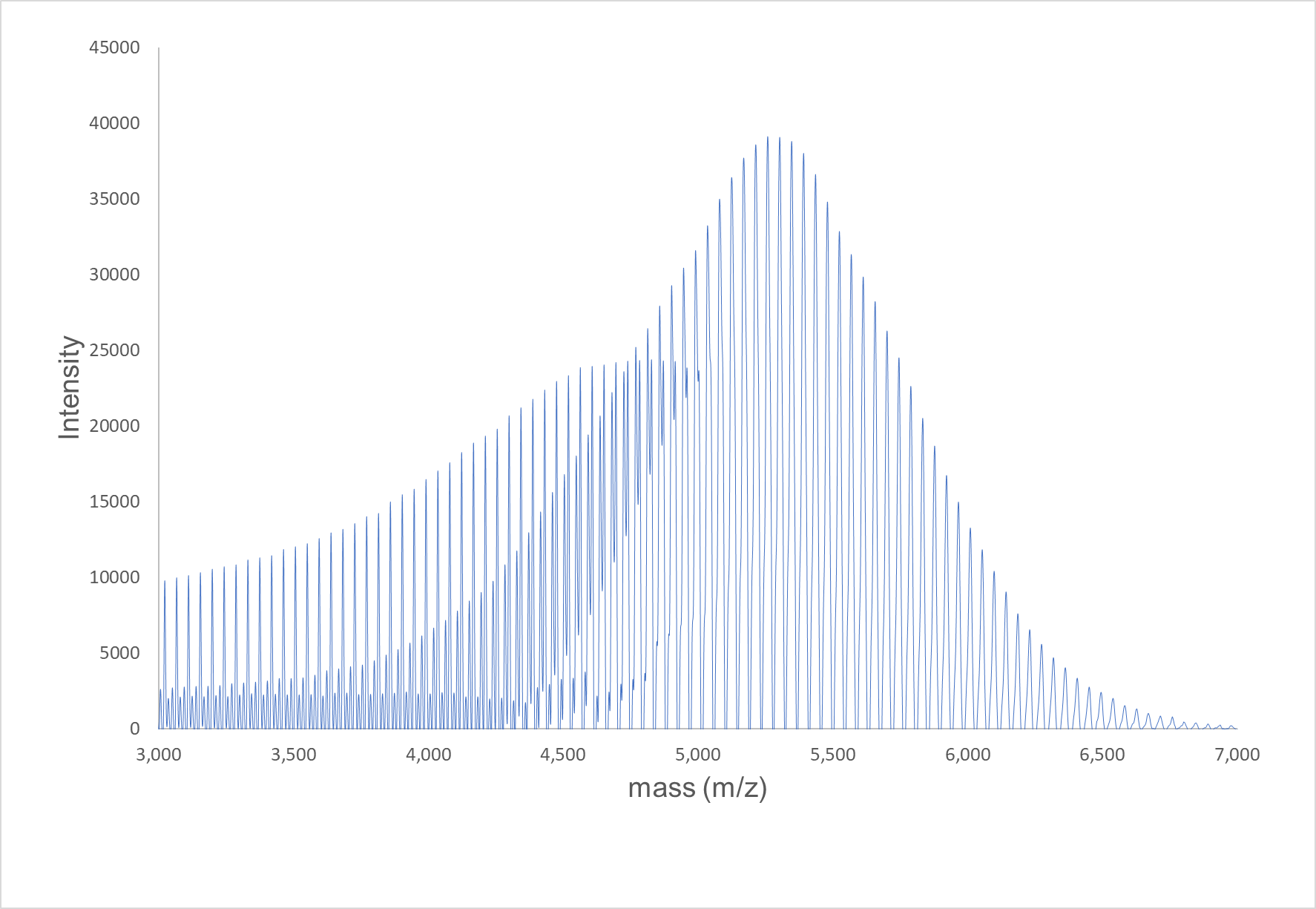

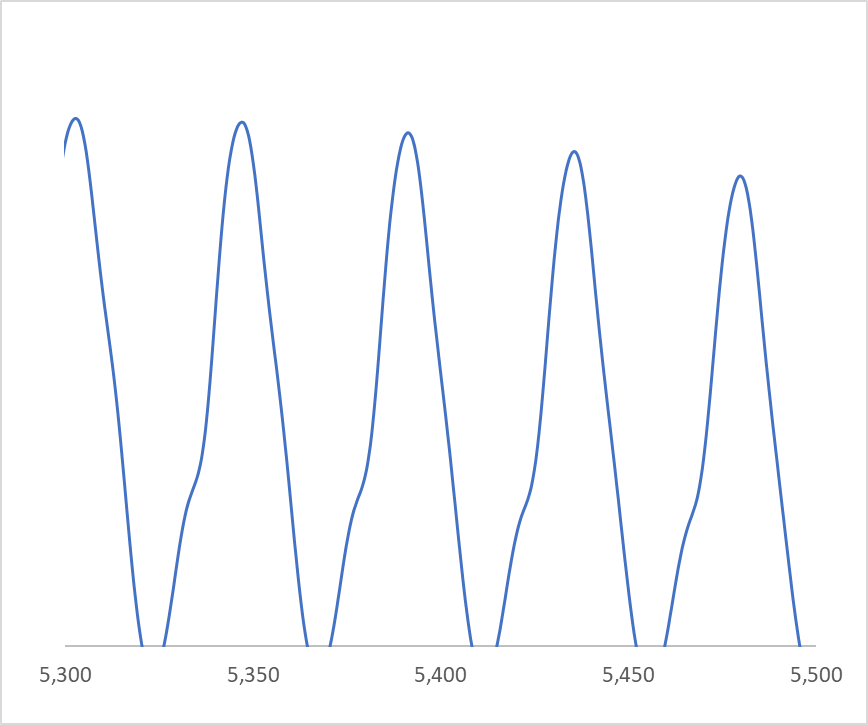


44.0 Da


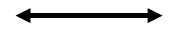


5436.35 [M + K]

5480.38 [M + K]

Figure S 42: MALDI-TOF mass spectrum of $\text{rPEG}_{\text{89}}^{\text{0.27}}$.


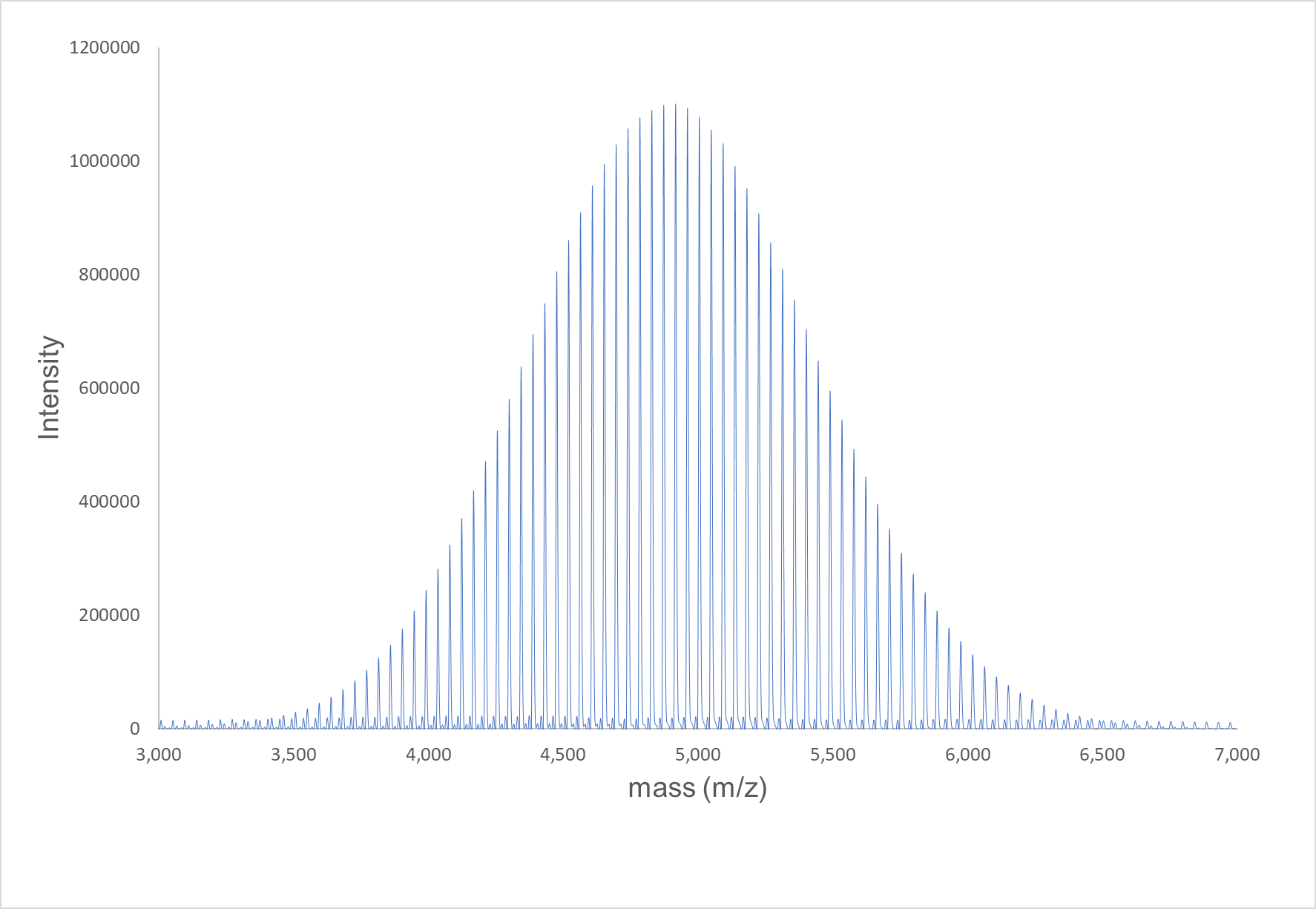

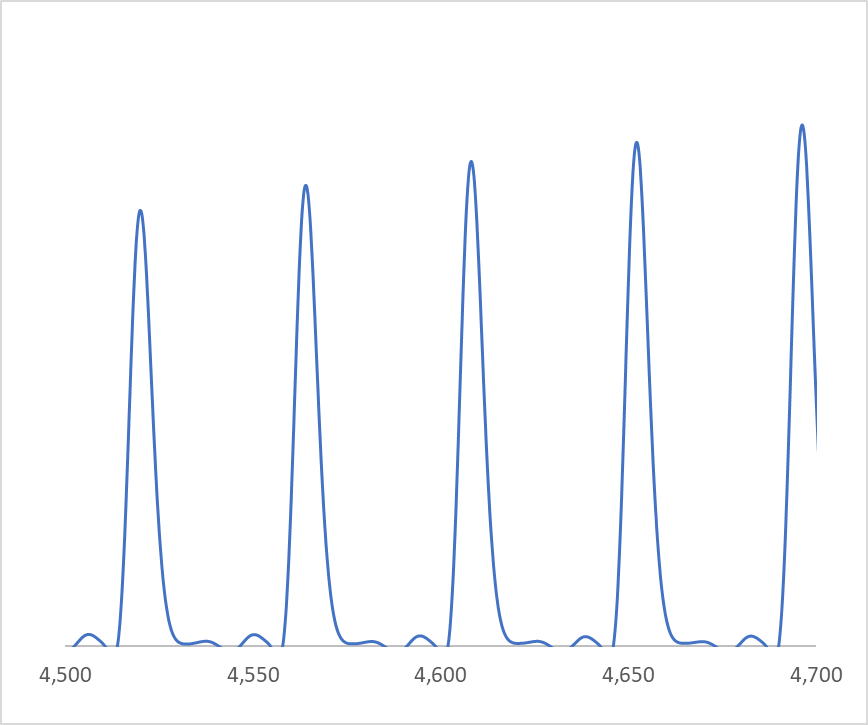


44.2 Da


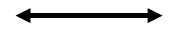


4563.35 [M + K]

4607.58 [M + K]

Figure S 43: MALDI-TOF mass spectrum of $\text{rPEG}_{\text{89}}^{\text{0.27}}$-mesylate.


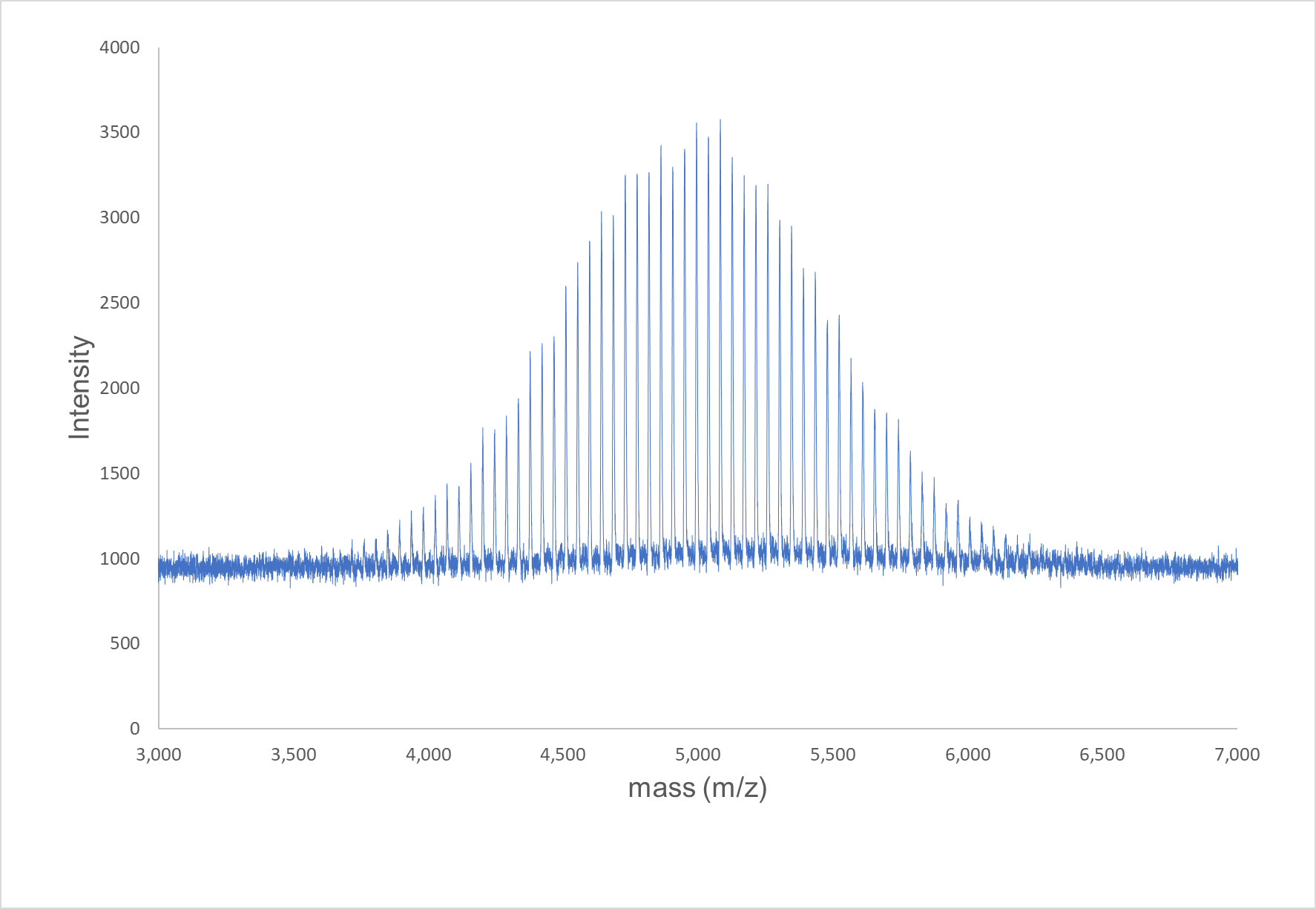

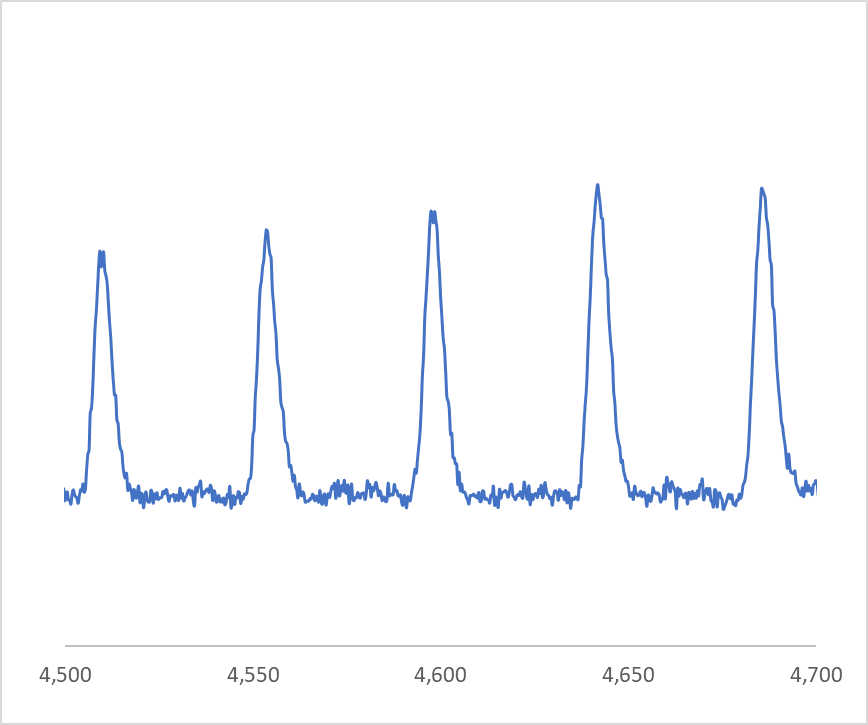


44.1 Da


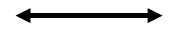


4597.48 [M + K]

4641.56 [M + K]

*Figure S 44: MALDI-TOF mass spectrum of* $\text{rPEG}_{\text{89}}^{\text{0.27}}$*-azide.*


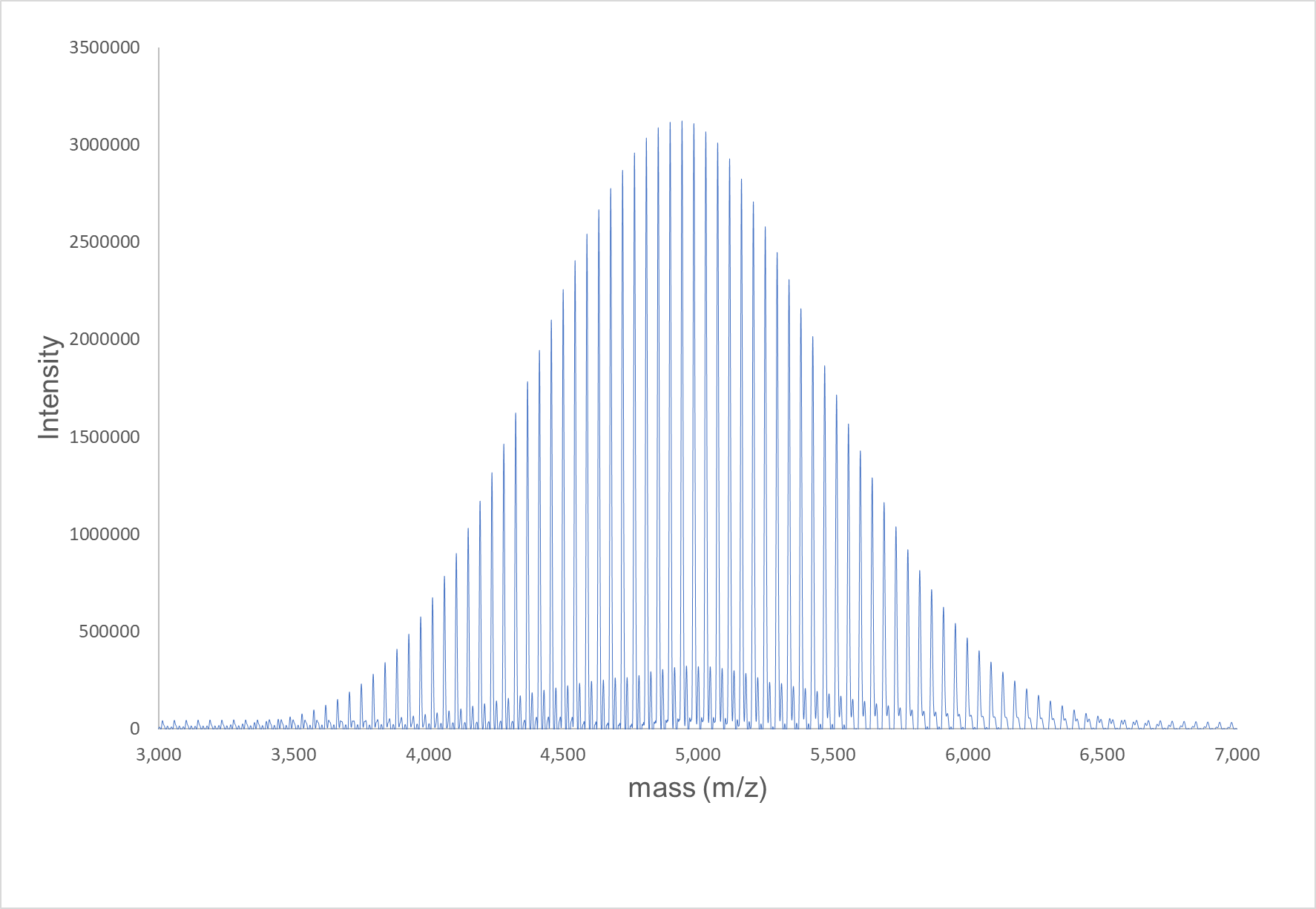

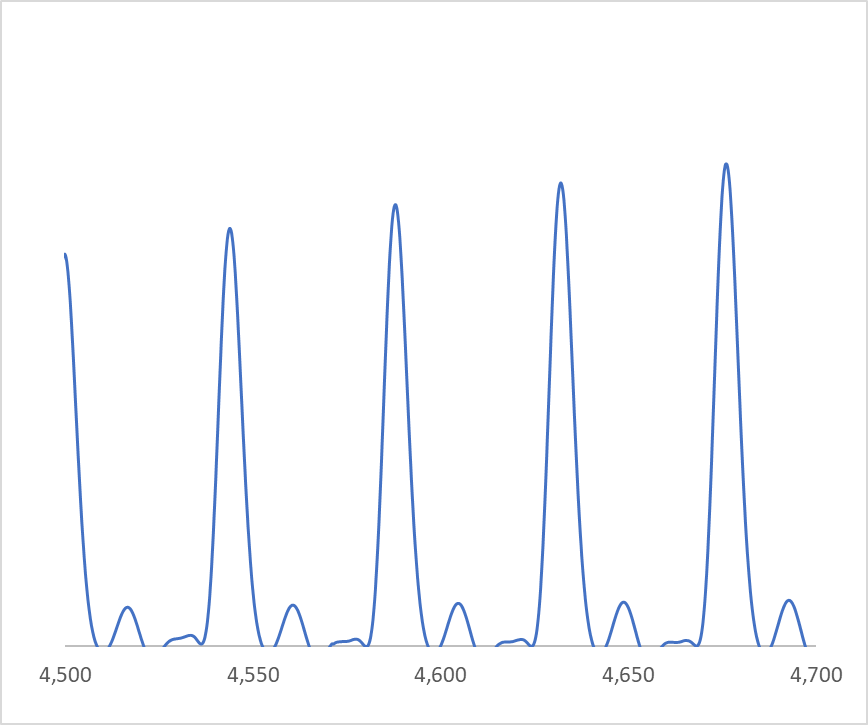


44.2 Da


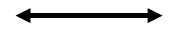


4632.07 [M + K]

4676.31 [M + K]

44.0 Da


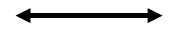


4516.74
[M - N_2_ + K]

4561.72
[M - N_2_ + K]

Figure S 45: MALDI-TOF mass spectrum of $\text{rPEG}_{\text{89}}^{\text{0.27}}$-amine.


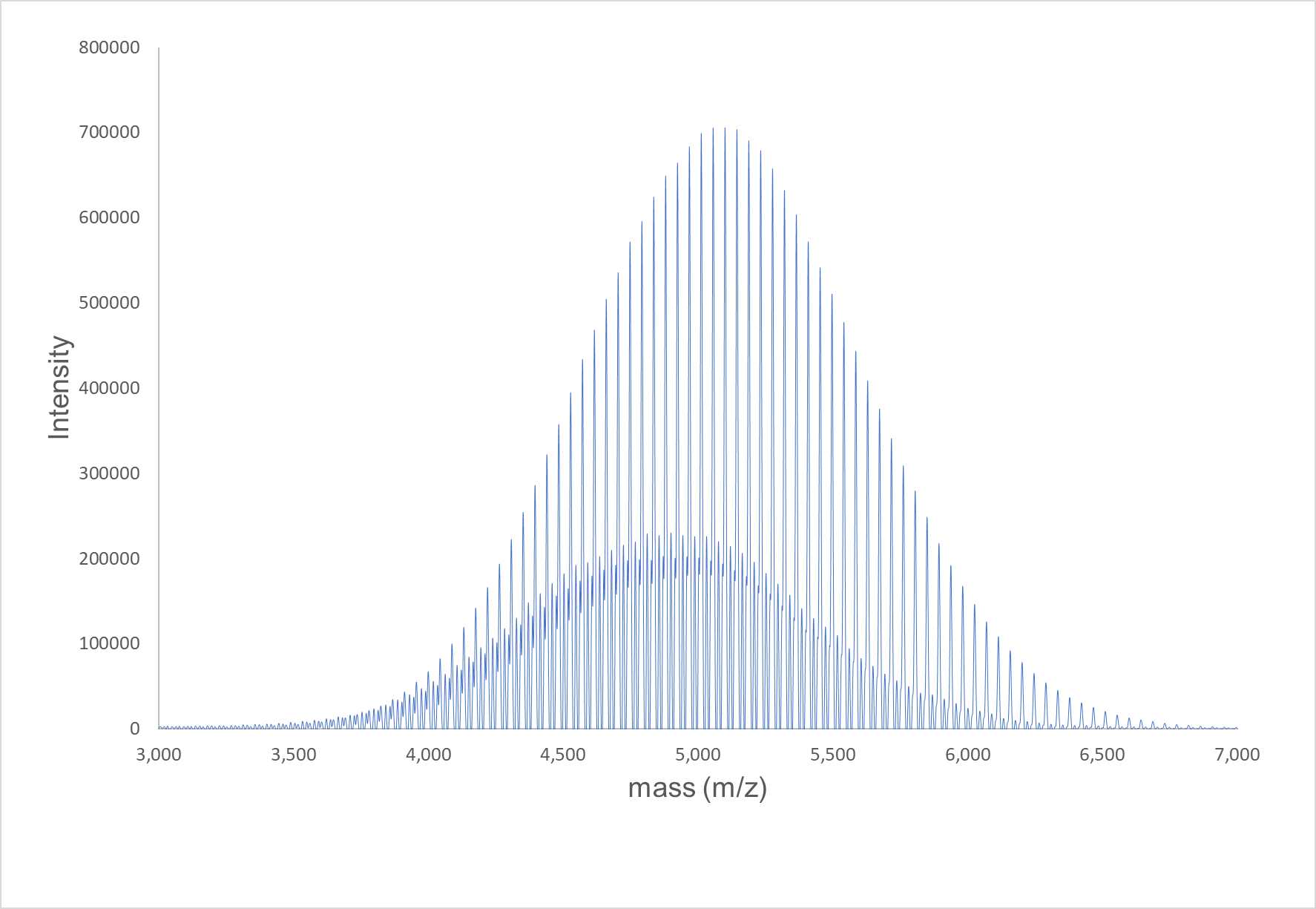

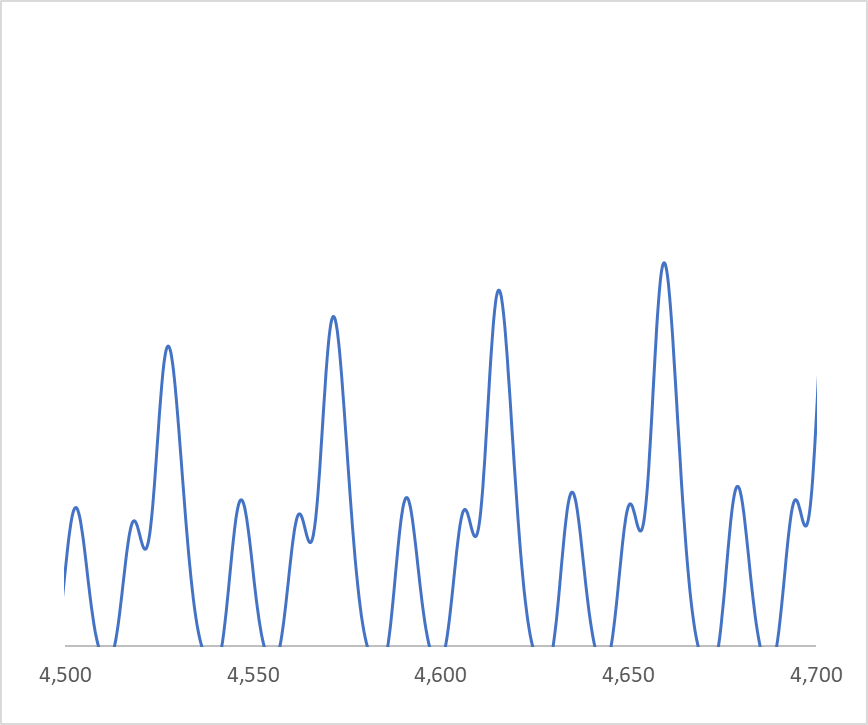


44.2 Da


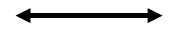


4613.45 [M + H]

4657.61 [M + H]


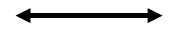

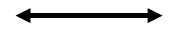


4546.49 [M + Na]

4562.37 [M + K]

Figure S 46: MALDI-TOF mass spectrum of $\text{rPEG}_{\text{89}}^{\text{0.27}}$-FITC.


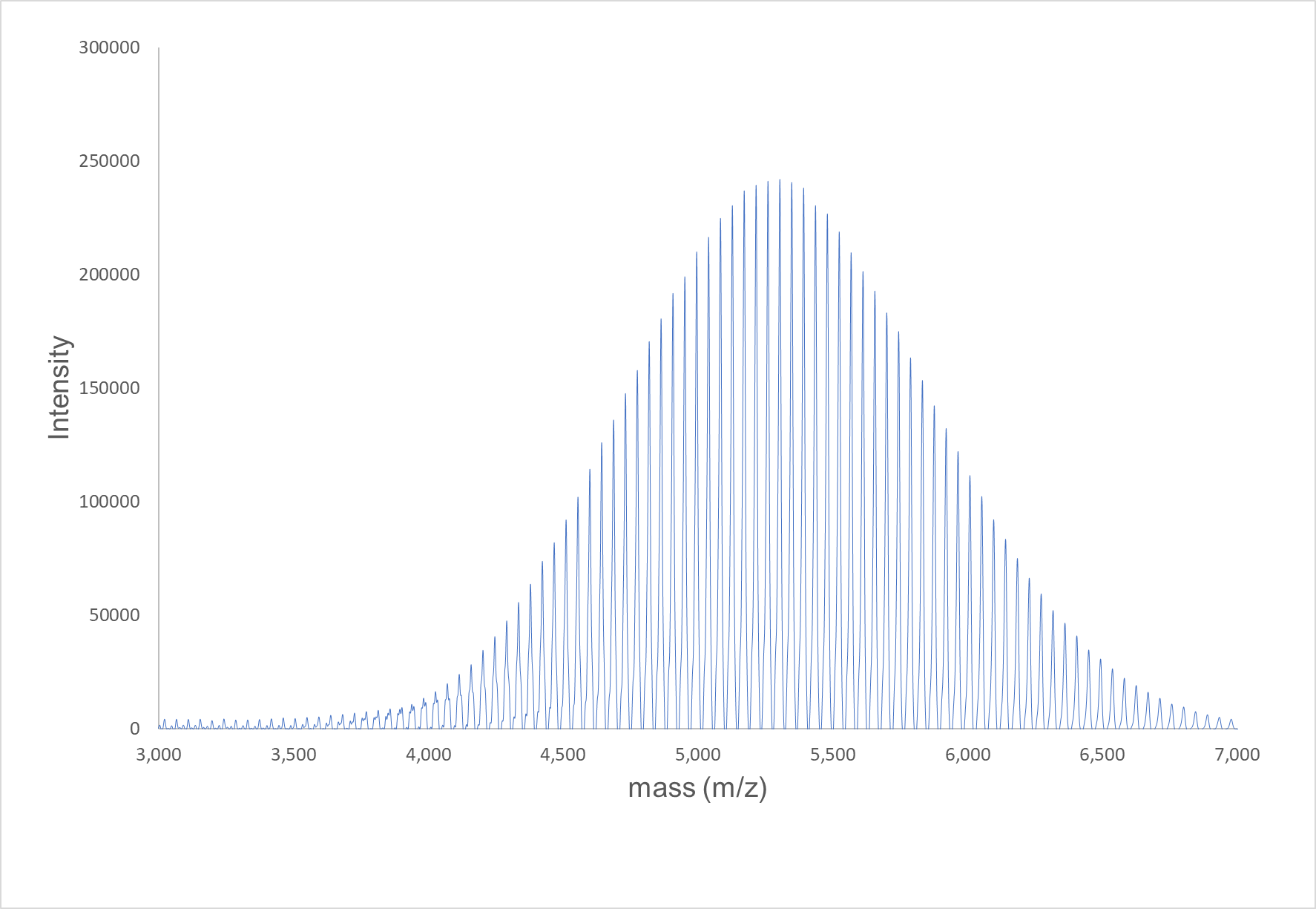

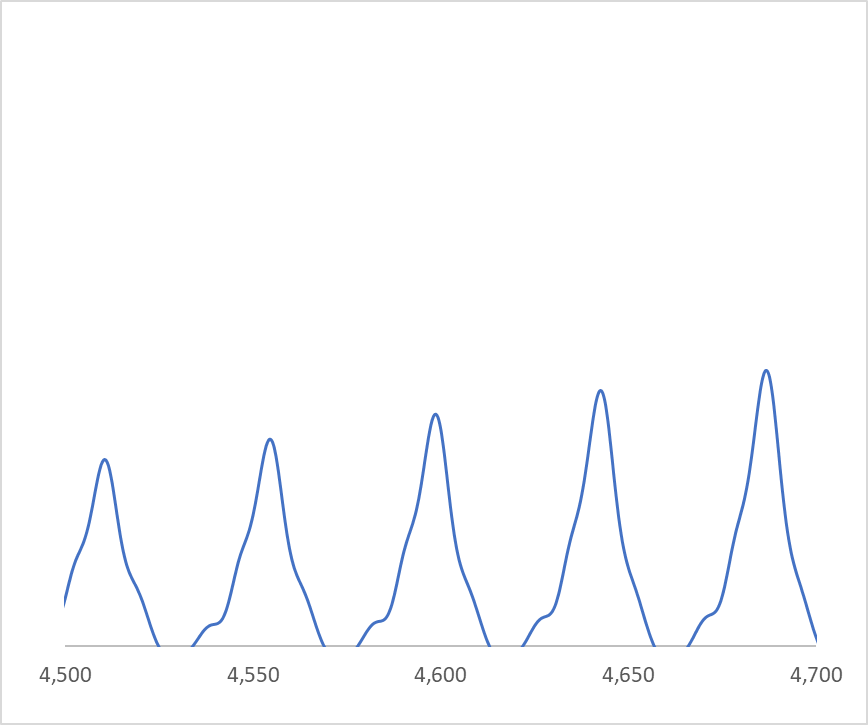


44.1 Da


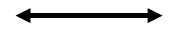


4598.78 [M + K]

4642.86 [M + K]

Figure S 47: MALDI-TOF mass spectrum of $\text{rPEG}_{\text{91}}^{\text{0.42}}$.


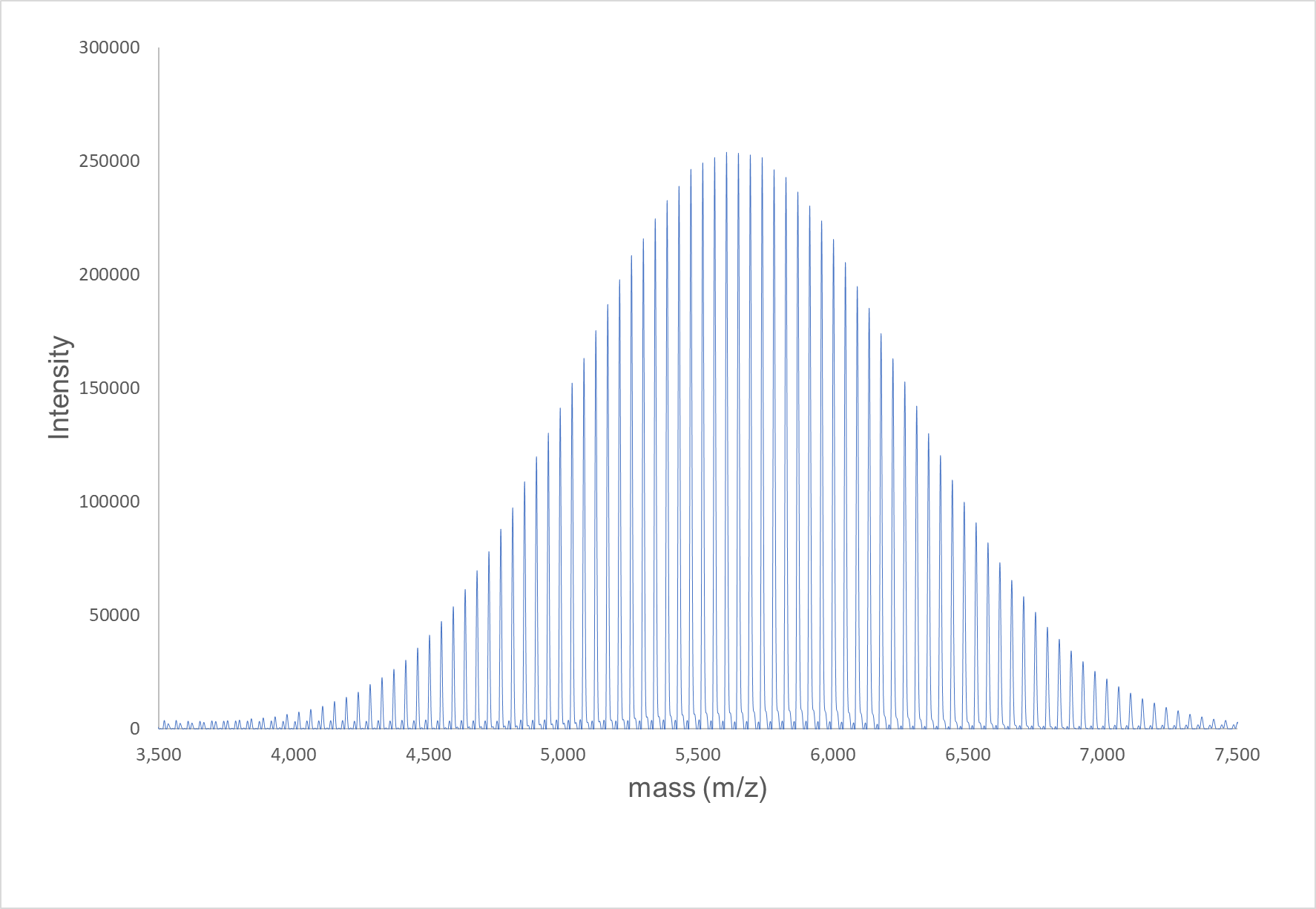

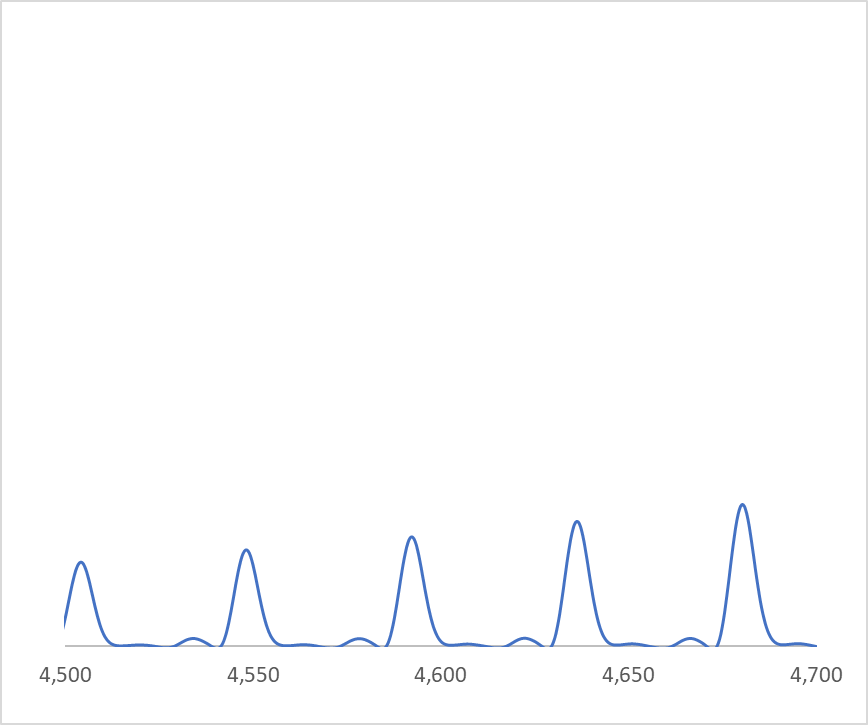


44.1 Da


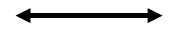


4591.62 [M + Na]

4635.67 [M + Na]

Figure S 48: MALDI-TOF mass spectrum of $\text{rPEG}_{\text{91}}^{\text{0.42}}$-mesylate.


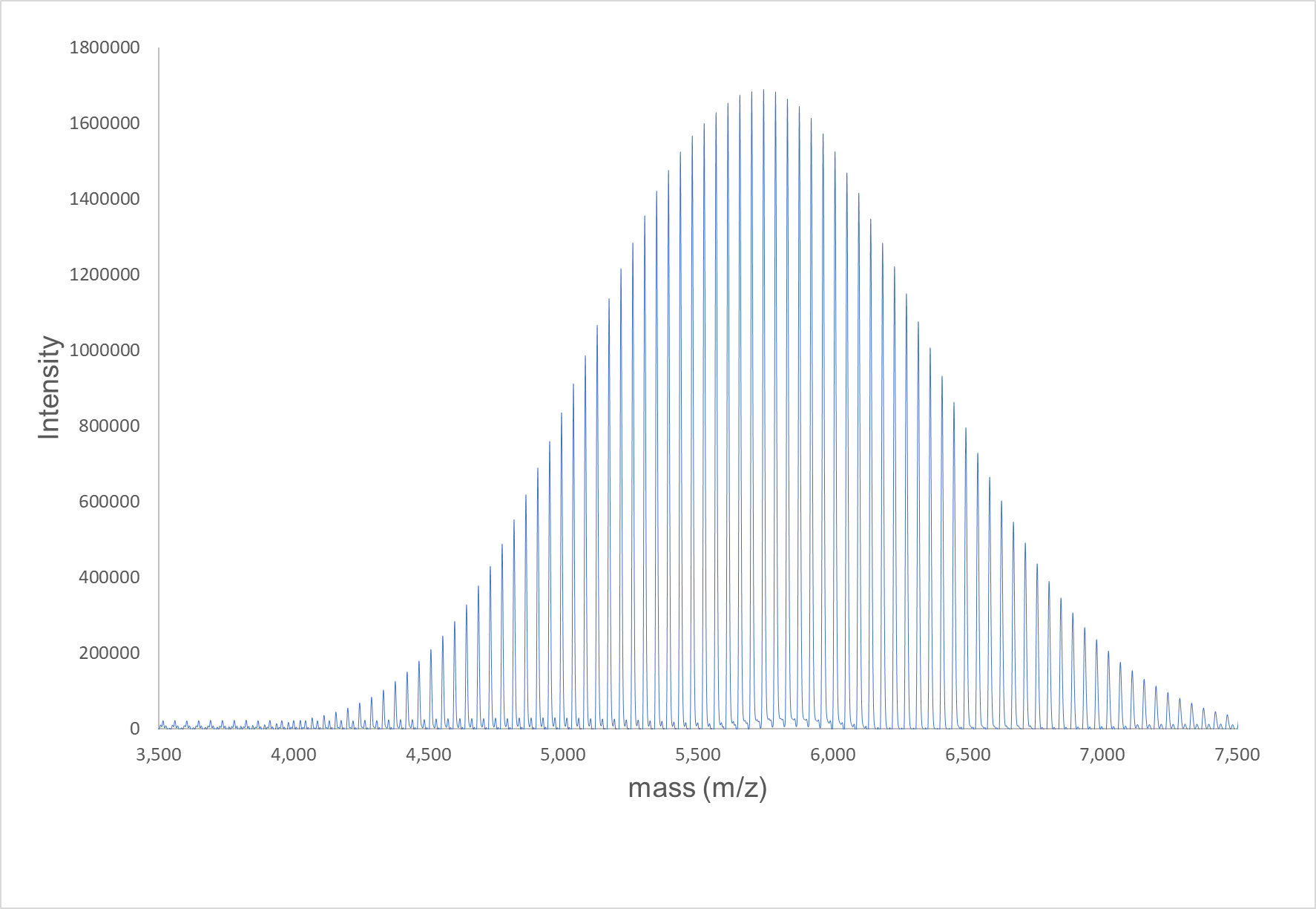

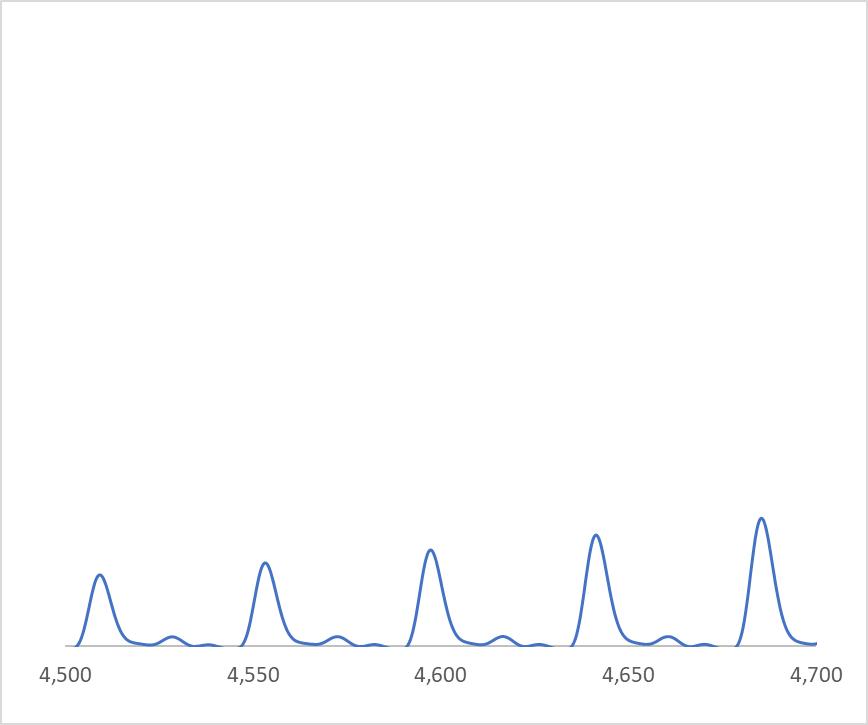


44.0 Da


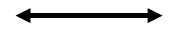


4597.48 [M + K]

4651.46 [M + K]

Figure S 49: MALDI-TOF mass spectrum of $\text{rPEG}_{\text{91}}^{\text{0.42}}$-azide.


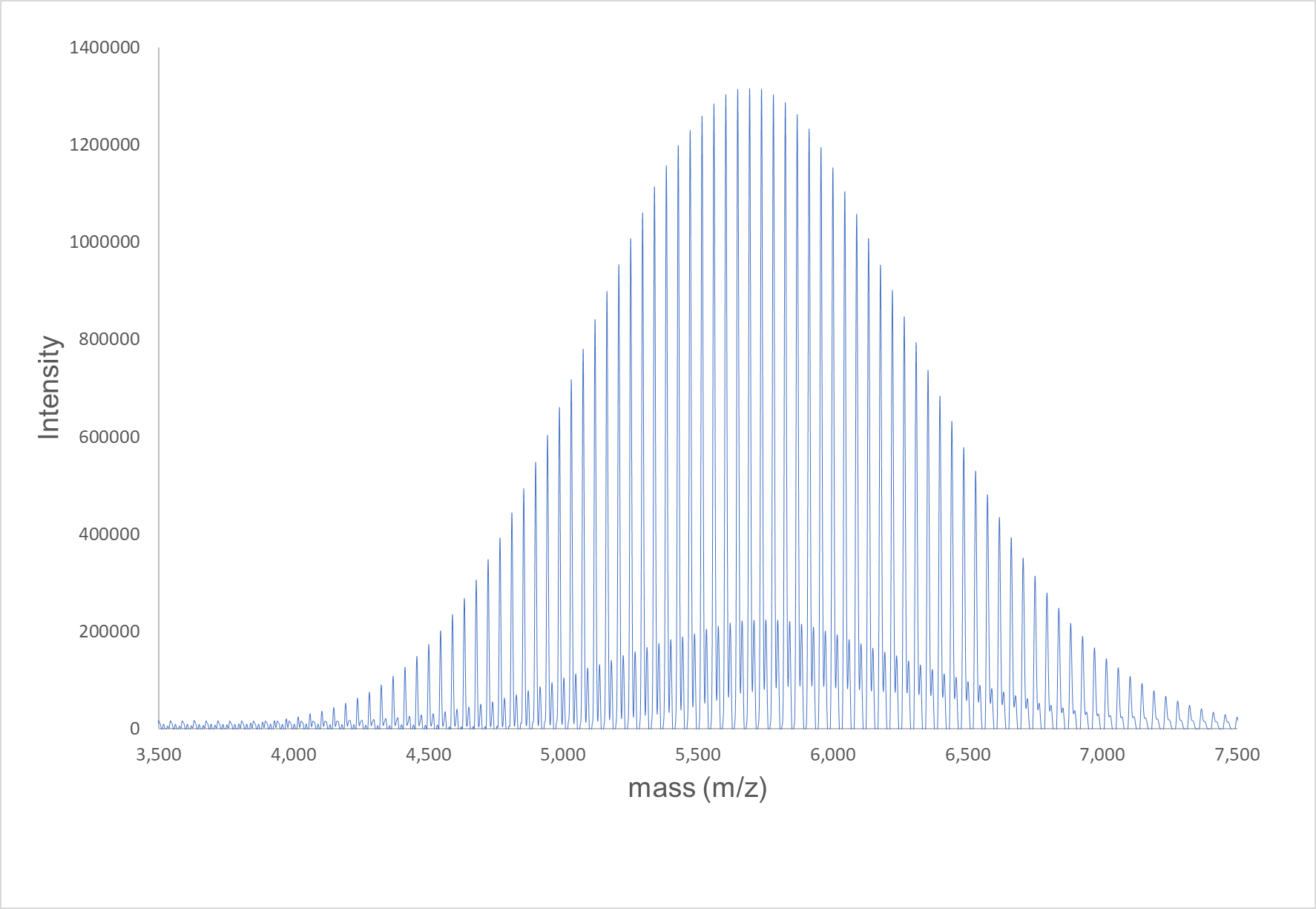

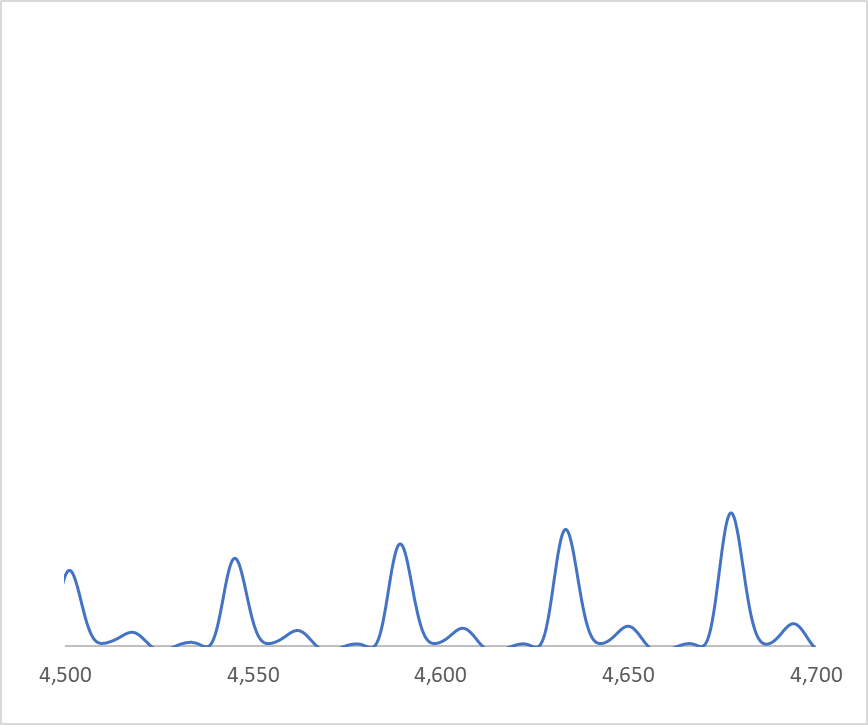


44.0 Da


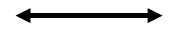


4632.72 [M + K]

4676.64 [M + K]

44.0 Da


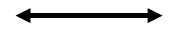


4516.74
[M - N_2_ + K]

4561.72
[M - N_2_ + K]

Figure S 50: MALDI-TOF mass spectrum of $\text{rPEG}_{\text{91}}^{\text{0.42}}$-amine.


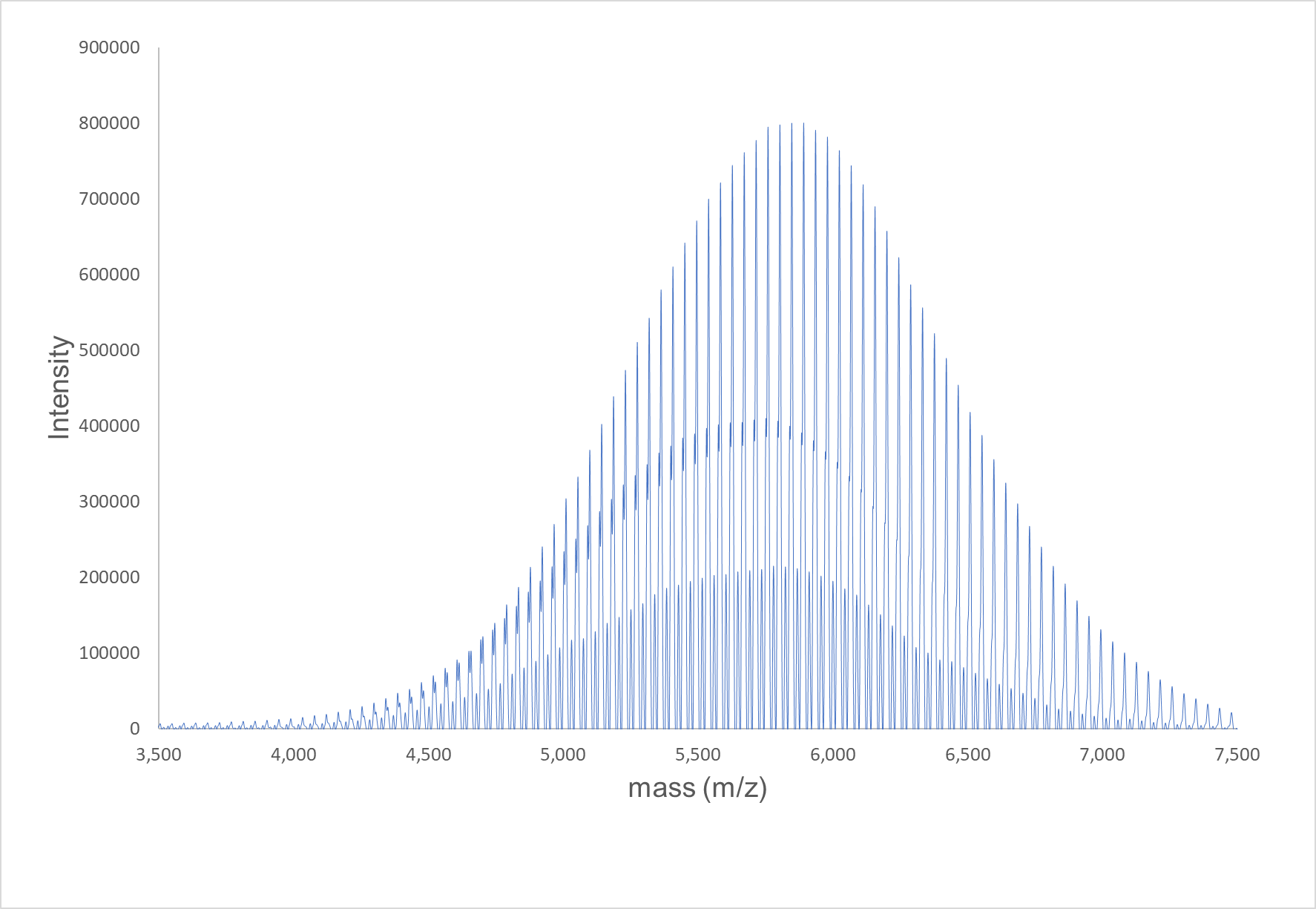

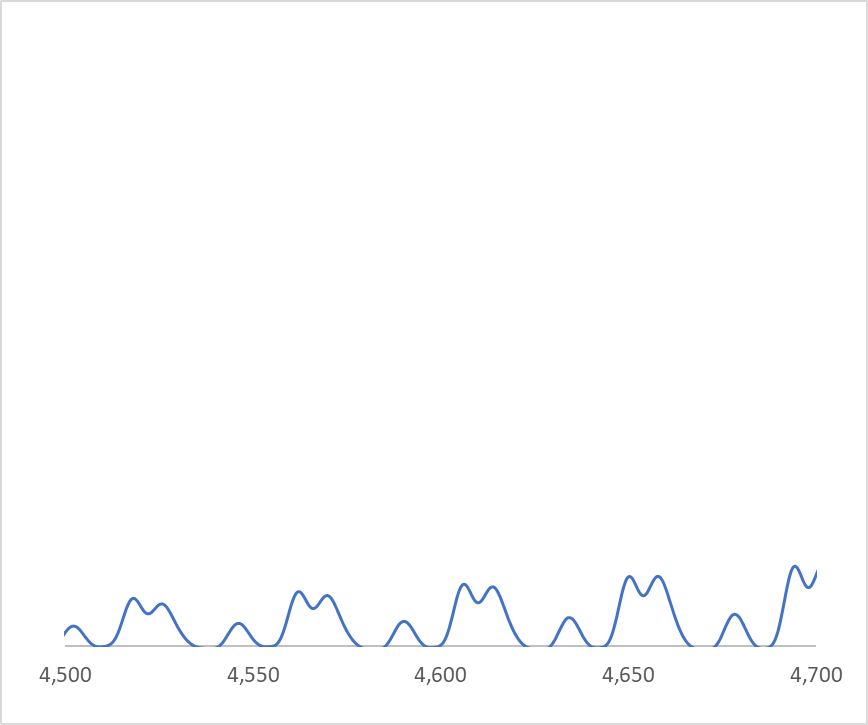


4546.16 [M + Na]

4562.37 [M + K]

44.1 Da


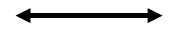


4612.47 [M + H]

4656.62 [M + H]


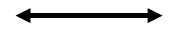

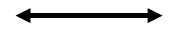


Figure S 51: MALDI-TOF mass spectrum of $\text{rPEG}_{\text{91}}^{\text{0.42}}$-FITC.


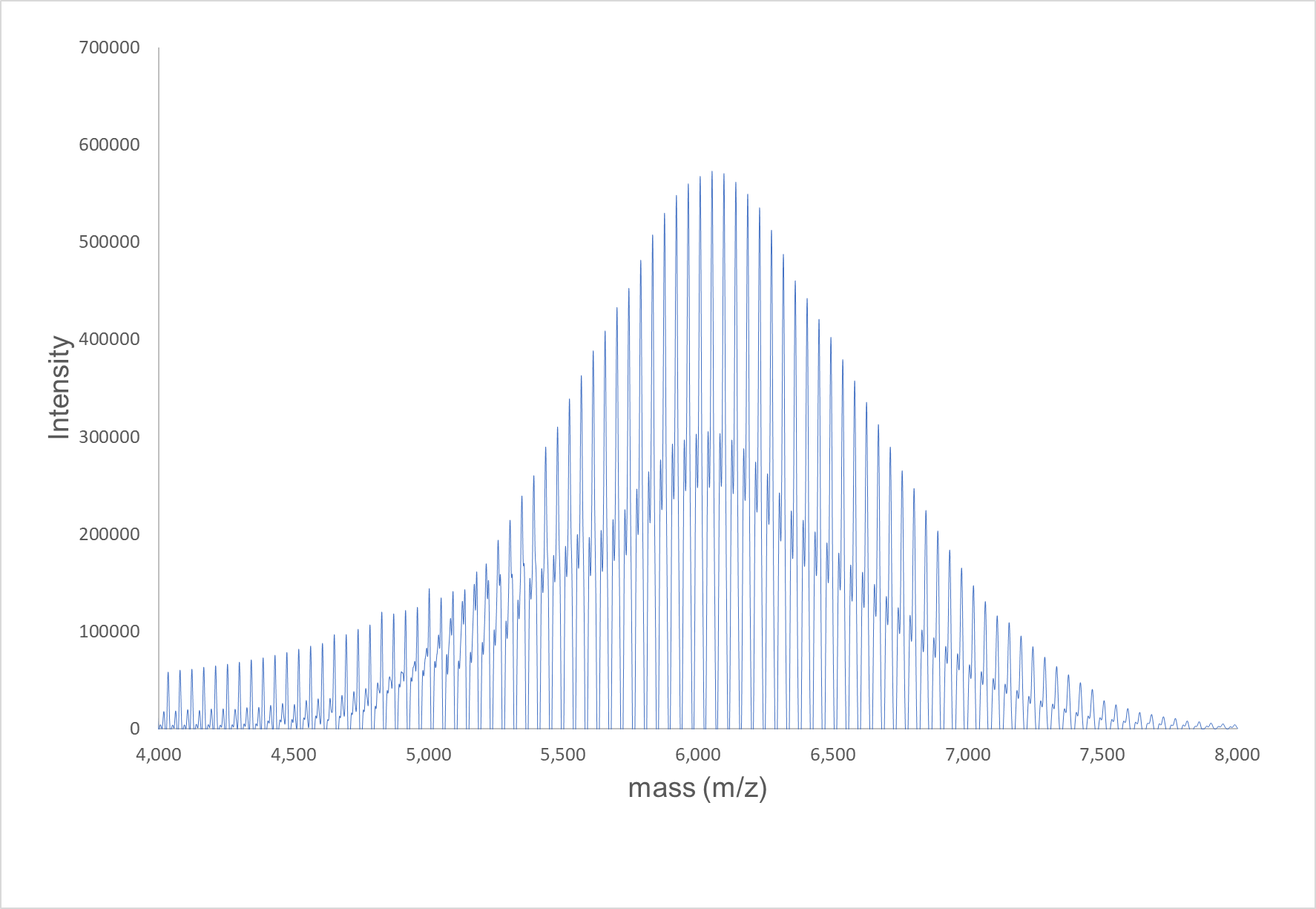


44.0 Da

5612.47 [M + K]

4656.49 [M + K]

44.0 Da

5508.52 [M + Na]

5552.49 [M + Na]

Figure S 52: MALDI-TOF mass spectrum of $\text{rPEG}_{\text{76}}^{\text{0.52}}$.

43.9 Da

4563.35 [M + K]

4607.26 [M + K]

Figure S 53: MALDI-TOF mass spectrum of $\text{rPEG}_{\text{76}}^{\text{0.52}}$-mesylate.

44.1 Da

4597.48 [M + K]

4641.55 [M + K]

Figure S 54: MALDI-TOF mass spectrum of $\text{rPEG}_{\text{76}}^{\text{0.52}}$-azide.

44.0 Da

4632.72 [M + K]

4676.64 [M + K]

44.0 Da

4516.74
[M - N_2_ + K]

4561.72
[M - N_2_ + K]

Figure S 55: MALDI-TOF mass spectrum of $\text{rPEG}_{\text{76}}^{\text{0.52}}$-amine.

44.2 Da

4612.80 [M + H]

4656.95 [M + H]

44.0 Da

4500.62
[M + Na]

4544.55
[M + Na]

Figure S 56: MALDI-TOF mass spectrum of $\text{rPEG}_{\text{76}}^{\text{0.52}}$-FITC.

44.0 Da

5083.33 [M + K]

5127.28 [M + K]

**5. Gel permeation chromatography (GPC) curves**

*Figure S 57: GPC curves of the unfunctionalized polymers (average molar mass M_n_ and dispersity Đ given).*

*Figure S 58: GPC curves of the mesylate-functionalized polymers (average molar mass M_n_ and dispersity Đ given).*

Figure S 59: GPC curves of the azide-functionalized polymers (average molar mass M_n_ and dispersity Đ given).

Figure S 60: GPC curves of the amine-functionalized polymers (average molar mass M_n_ and dispersity Đ given).

Figure S 61: GPC curves of the FITC-functionalized polymers (average molar mass M_n_ and dispersity Đ given).

**6. Microscale thermophoresis (MST) curves**

Figure S 62: Individual MST measurements

Figure S 62: MST data displaying individual data points of technical triplicates (measurement of three capillaries)

Figure S 63: MST measurement results plotted as fraction bound depending on ligand (polymer) concentration (mean $\pm$ S.D., n = 3 technical replicates).

**7. Fluorescence Correlation Spectroscopy (FCS) measurements in human plasma**

Figure S 64: Autocorrelation functions (ACFs) derived from FCS measurements of all polymers (c = 20 nM) in human citrate plasma, shown together with the corresponding fits (double-component diffusion models, where the small species refers to free dye and larger species refers to labeled polymer). The hydrodynamic radius is reported for the largest diffusing species in each sample. Error refers to the typical experimental error of ± 10% for fluorescence correlation spectroscopy. Each FCS experiment included ten technical replicates per sample, acquired at 10-second intervals.
